# Global analysis of lysine succinylation in patchouli plant leaves

**DOI:** 10.1038/s41438-019-0216-5

**Published:** 2019-12-01

**Authors:** Xiaobing Wang, Xiuzhen Chen, Junren Li, Xuanxuan Zhou, Yanting Liu, Liting Zhong, Yun Tang, Hai Zheng, Jiyun Liu, Ruoting Zhan, Likai Chen

**Affiliations:** 1 Research Center of Chinese Herbal Resource Science and Engineering, Guangzhou University of Chinese Medicine; Key Laboratory of Chinese Medicinal Resource from Lingnan (Guangzhou University of Chinese Medicine), Ministry of Education; Joint Laboratory of National Engineering Research Center for the Pharmaceutics of Traditional Chinese Medicines, Guangzhou, Guangdong, 510006 P. R. China; 2 Guangdong Institute of Traditional Chinese Medicine, Guangzhou, Guangdong, 510520 P. R. China; 3 Key Laboratory of the Ministry of Education for Coastal and Wetland Ecosystems, College of the Environment and Ecology, Xiamen University, Xiamen, Fujian 361005 P.R. China

**Keywords:** Proteomics, Plant development

## Abstract

Lysine succinylation is a novel, naturally occurring posttranslational modification (PTM) in living organisms. Global lysine succinylation identification has been performed at the proteomic level in various species; however, the study of lysine succinylation in plant species is relatively limited. Patchouli plant (*P. cablin* (Blanco) Benth., Lamiaceae) is a globally important industrial plant and medicinal herb. In the present study, lysine succinylome analysis was carried out in patchouli plants to determine the potential regulatory role of lysine succinylation in patchouli growth, development, and physiology. The global succinylation sites and proteins in patchouli plants were screened with an immunoprecipitation affinity enrichment technique and advanced mass spectrometry-based proteomics. Several bioinformatic analyses, such as function classification and enrichment, subcellular location predication, metabolic pathway enrichment and protein−protein interaction networking, were conducted to characterize the functions of the identified sites and proteins. In total, 1097 succinylation sites in 493 proteins were detected in patchouli plants, among which 466 succinylation sites in 241 proteins were repeatedly identified within three independent experiments. The functional characterization of these proteins indicated that the tricarboxylic acid (TCA) cycle, oxidative phosphorylation, photosynthesis processes, and amino acid biosynthesis may be regulated by lysine succinylation. In addition, these succinylated proteins showed a wide subcellular location distribution, although the chloroplast and cytoplasm were the top two preferred cellular components. Our study suggested the important role of lysine succinylation in patchouli plant physiology and biology and could serve as a useful reference for succinylation studies in other medicinal plants.

## Introduction

Protein posttranslational modifications (PTMs) participate in diverse cellular events and biological processes in living organisms through various mechanisms^1–3^. With the development of mass spectrometry (MS) and bioinformatics, increasing novel PTMs have been identified, and their roles in cellular and metabolic processes have been studied^4^. More than 461 distinct PTMs have been reported in living organisms^5^, among which lysine succinylation is a novel naturally occurring PTM in eukaryotes and prokaryotes affecting various cellular events and metabolism processes^6,7^.

The identification of global lysine succinylation sites at the proteomic level, namely, protein succinylome, was performed in diverse organisms, including microorganisms, animals, and plants^7,8^. In microorganisms, the succinylome of various bacteria and fungi, such as *Escherichia coli*, *Saccharomyces cerevisiae*, *Candida albicans*, *Aspergillus flavus*, and *Trichophyton rubrum*, has been reported^7,9–11^. Hundreds and even thousands of succinylated sites and proteins have been identified in these species. Moreover, almost all key biological processes and metabolic pathways are influenced by lysine succinylation modification in these microorganisms. In animals, especially mammals, relatively extensive succinylation research was performed. A total of 2565 succinylation sites corresponding to 779 proteins have been reported in mouse cells, which affect diverse metabolic pathways including amino acid degradation, citrate cycle (TCA cycle), and fatty acid metabolism^12^. In humans, quantitative succinylome analyses were conducted in various clinical tissues and cell lines to reveal the role of protein succinylation under physiological or pathological conditions, and the results showed that the progression of some diseases, such as fat liver, renal cell carcinoma, and gastric cancer, was related to protein succinylation^13–15^.

In plants, the succinylome in Gramineae plants, including some model plants and food crops, has been most commonly studied. A study on the rice succinylome indicated that lysine succinylation participated in rice seed germination, seedling growth and responses to oxidative stress^8,16^. In addition, 330 succinylated sites in 173 proteins in wheat and 605 succinylated sites in 262 proteins in *Brachypodium distachyon* L. have been reported^17,18^. A systematic lysine succinylation analysis in horticultural specifies, including strawberry, tomato, tea, *Dendrobium officinale* (*D. officinale*), and yew, was also reported^19–23^. Even with these studies, the study of lysine succinylation in plants is relatively limited compared with that in mammals and microorganisms and still requires further exploration.

Patchouli plant [*Pogostemon cablin* (Blanco) Benth. (Lamiaceae)] is an important industrial plant and medicinal herb that has been widely cultivated in tropical and subtropical areas of Asia^24^. Patchouli oil is widely used in the perfume industry because of the basic and lasting fragrances of this natural essential oil^25^. In addition, patchouli plants are recorded in the Chinese Pharmacopoeia as a traditional Chinese medicine because of their therapeutic role. It exhibits a wide range of medicinal effects, including anti-inflammatory, antibacterial, anti-influenza virus, cytotoxic, platelet aggregation inhibition, hepatoprotective, antidepressant, aphrodisiac, febrifuge, astringent, carminative, diuretic, and sedative properties^25,26^.

Previous researchers reconstructed the patchouli alcohol and pogostone metabolic pathway through analysis of the differentially expressed genes in patchouli leaves and stems with transcriptome data^27^. A proteomic study showed that energy metabolism was critical for patchouli plants to resist the stress of the continuous cropping^26^. Lysine succinylation is an important PTM in cellular events and metabolic pathway regulation. However, the lysine succinylation study in patchouli plants at the proteomic level has not been reported, and the role of lysine succinylation in patchouli plant physiology and biology needs to be elucidated.

We performed succinylome analysis in patchouli plant seedlings with antibody-based immunoprecipitation affinity enrichment, advanced mass spectrometry-based proteomics and powerful bioinformatics in the present study. We aim to identify potential succinylation substrates and describe the functional characteristics of lysine succinylome in patchouli plants. To date, this is the first lysine succinylome study in patchouli plants. Our study may accelerate and benefit the detailed elucidation of the potential regulatory role of lysine succinylation in plant physiology and biology.

## Results

### Identification of lysine succinylation in patchouli

**Fig. 1:**
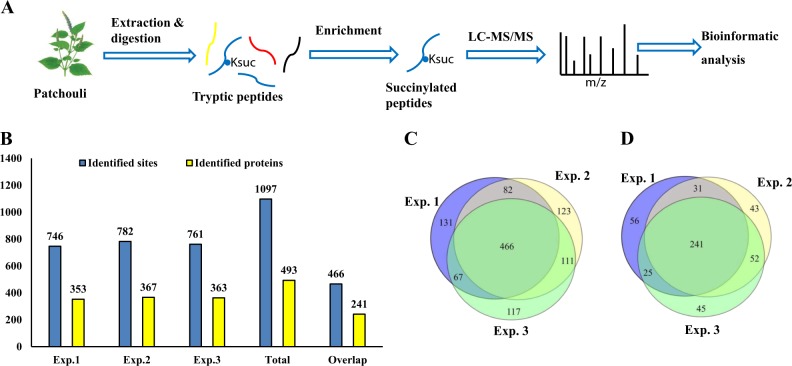
Identification of the global succinylation sites and proteins in patchouli plant leaves. **a** Workflow for global lysine succinylation identification in patchouli plant leaves. **b** Statistical analysis of identified succinylated sites and proteins in three replicate experiments. **c** Venn diagram of the identified succinylated sites. **d** Venn diagram of the identified succinylated proteins.

Patchouli is a well-known medicinal plant in traditional Chinese medicine. A qualitative lysine succinylome analysis was conducted in patchouli plants in this study, with the purpose of uncovering the role of lysine succinylation in patchouli physiology and biology. The general experimental design and workflow are shown in Fig. 1a. Briefly, after 4 weeks of cultivation in a greenhouse, the leaf tissues were cut and washed carefully. Then, the succinylomic sample preparation procedure was carried out, which included, protein extraction, trypsin digestion, and antibody-based succinylated peptide affinity enrichment. The succinylated peptides and proteins were identified via liquid chromatography tandem mass spectrometry (LC-MS/MS). All the MS data were deposited to ProteomeXchange Consortium^28^ via the PRIDE partner repository with the accession number PXD015567. Finally, bioinformatics analysis was performed to systematically interpret these identified sites and proteins. Three independent replicate experiments were performed. Only the succinylated sites and proteins repeatedly identified in the three independent experiments were selected for the bioinformatics analysis to characterize the succinylome in patchouli plants.

The detailed identification results from the three replicates are provided in Fig. 1 and Supporting Table S1. In total, 1097 succinylation sites corresponding to 493 proteins were detected, among which 466 succinylation sites corresponding to 241 proteins were repeatedly identified in the three independent experiments (Fig. 1), indicating that lysine succinylation was a widespread PTM in various patchouli proteins. We summarized and compared the number of succinylation sites and succinylation proteins in various horticulture plant species, combining our study and previous reports (Supporting Table S2) on species including strawberry, tomato, tea, *Taxus* and *D. officinale*^19–23^. Undoubtedly, there were some variations in the identified succinylation sites and proteins in different species, which may be related to multiple reasons.

### Site properties of the succinylated peptides

**Fig. 2:**
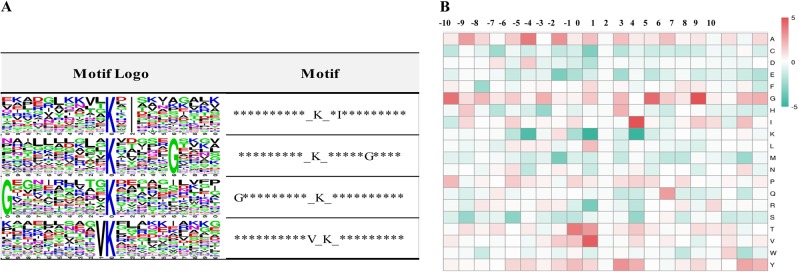
Motif analysis of the detected succinylation sites. **a** Succinylation sequence motifs for amino acids around the lysine succinylated sites (−10 to +10). The letter height represents the frequency of that amino acid residue at that position. The K in the middle position corresponds to the succinylated lysine. **b** Heat map analysis of the amino acid compositions around the succinylated sites. Red indicates an amino acid that is significantly enriched, while green indicates an amino acid that is significantly reduced.

To show the features of the succinylated peptides in patchouli plants, motif analysis was conducted with the Motif-X program. The program computes the probability analysis of the amino acids that are overrepresented or underrepresented at the positions adjacent to succinylation sites. All repeatedly identified 466 succinylation sites were used for this analysis. Four consensus sequence motifs were extracted, Ksuc*I, Ksuc*****G, G********* Ksuc, and V Ksuc (Ksuc indicates a succinylated lysine, while * indicates a random amino acid residue, Fig. 2a). The two glycine (G)-containing consensus sequences suggested that G may be a common amino acid either upstream or downstream of the succinylated sites.

The amino acid sequences around the succinylated lysine sites were examined via heat map analysis to detect the specific amino acids adjacent to lysine succinylation sites (Fig. 2b). Consistent with the motif result, we noticed that the overall frequency of the presence of G at every position (−10 to +10) surrounding a succinylated lysine showed a slightly increased trend. Moreover, its frequency in the −10, +3, and +6 positions was significantly higher. The heap map results also showed that alanine (A) was another common amino acid surrounding succinylated lysine sites and was significantly overrepresented in the −9, −5, and −3 positions. In addition, isoleucine (I) was overrepresented in the +2 position, while valine (V) was overrepresented in the −1 position, which was consistent with the motif analysis result.

### Function classification and subcellular location analysis

**Fig. 3:**
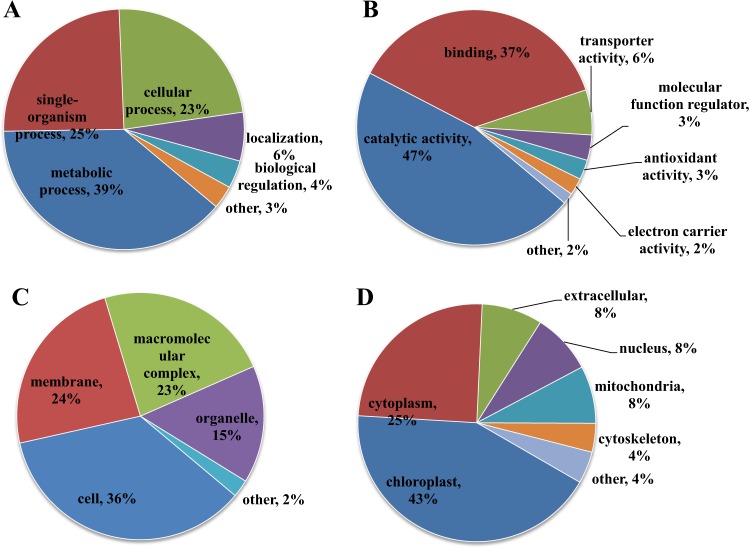
Classification and subcellular location analysis of the identified succinylation proteins. Gene ontology-based classification analysis of the identified succinylation proteins in the categories biological process (**a**), molecular function (**b**), and cellular component (**c**) and subcellular localization from WoLF PSORT (**d**).

Gene ontology (GO)-based classification in the categories biological process, cellular component and molecular function and WoLF PSORT-based subcellular prediction analysis were carried out to demonstrate the potential roles of lysine succinylation in patchouli plants (Fig. 3).

The classification analysis in the category biological process showed that most of the succinylated proteins were involved in metabolic processes (39%), single-organism processes (25%) and cellular processes (23%). Biological processes related to localization (6%) and biological regulation (4%) were also observed (Fig. 3a). Consistently, the molecular function results showed that proteins related to catalytic and binding activities were the major succinylated proteins in patchouli plants, accounting for 47 and 37% of all the identified proteins, respectively (Fig. 3b). For cellular component analysis, the succinylated proteins were distributed at the cell (36%), membrane (24%), macromolecular complex (23%), organelle (15%) and other (2%) (Fig. 3c). Subcellular location analysis showed that chloroplast (43%) and cytoplasm (25%) proteins were the dominant succinylated proteins, followed by extracellular (8%), nucleus (8%) and mitochondria (8%). A very small proportion of proteins were predicted to localize to the cytoskeleton (4%) or others (4%) (Fig. 3d). GO functional classification and subcellular location results suggested that the succinylated proteins participated in various biological processes and were located in diverse cellular compartments in patchouli plants.

We compared the function classification and the subcellular location distribution of the identified succinylated proteins between patchouli plants and other various horticulture plant species (Supporting Fig. S1)^19–23^. As shown in Fig. S1, most species in the figure showed a similar trend in function classification and subcellular location distribution except for strawberry, even though the detailed percentage of each item was different. In most species, the top three items at the biological process level were metabolic processes, single-organism processes, and cellular processes. Catalytic and binding activities were the two highest terms at the molecular function level. In addition, the majority of succinylated proteins were located in the chloroplast and cytoplasm in most horticulture species. Strawberry showed some differences in the functional distribution and subcellular location of succinylated proteins. Notably, it has relatively more transport activity-related succinylation proteins and a larger proportion of nucleus-localized succinylation proteins. Moreover, no chloroplast-localized succinylation protein was found. These phenomena may be highly related to the plant organs and tissues used in the analysis of strawberry. The researcher selected stigma, which is mainly responsible for the pollination process and plant reproduction in plant physiology, as a material^19^. In other horticulture species, four research teams selected leaves or leaves in addition to roots and stems as materials, while one used bark as materials^20–23^.

### Functional enrichment analysis of succinylated proteins

Functional enrichment analysis based on GO, Kyoto Encyclopedia of Genes and Genomes (KEGG) pathway and protein domain were carried out to reveal the characteristics of succinylated proteins in patchouli plants in greater detail.

**Fig. 4:**
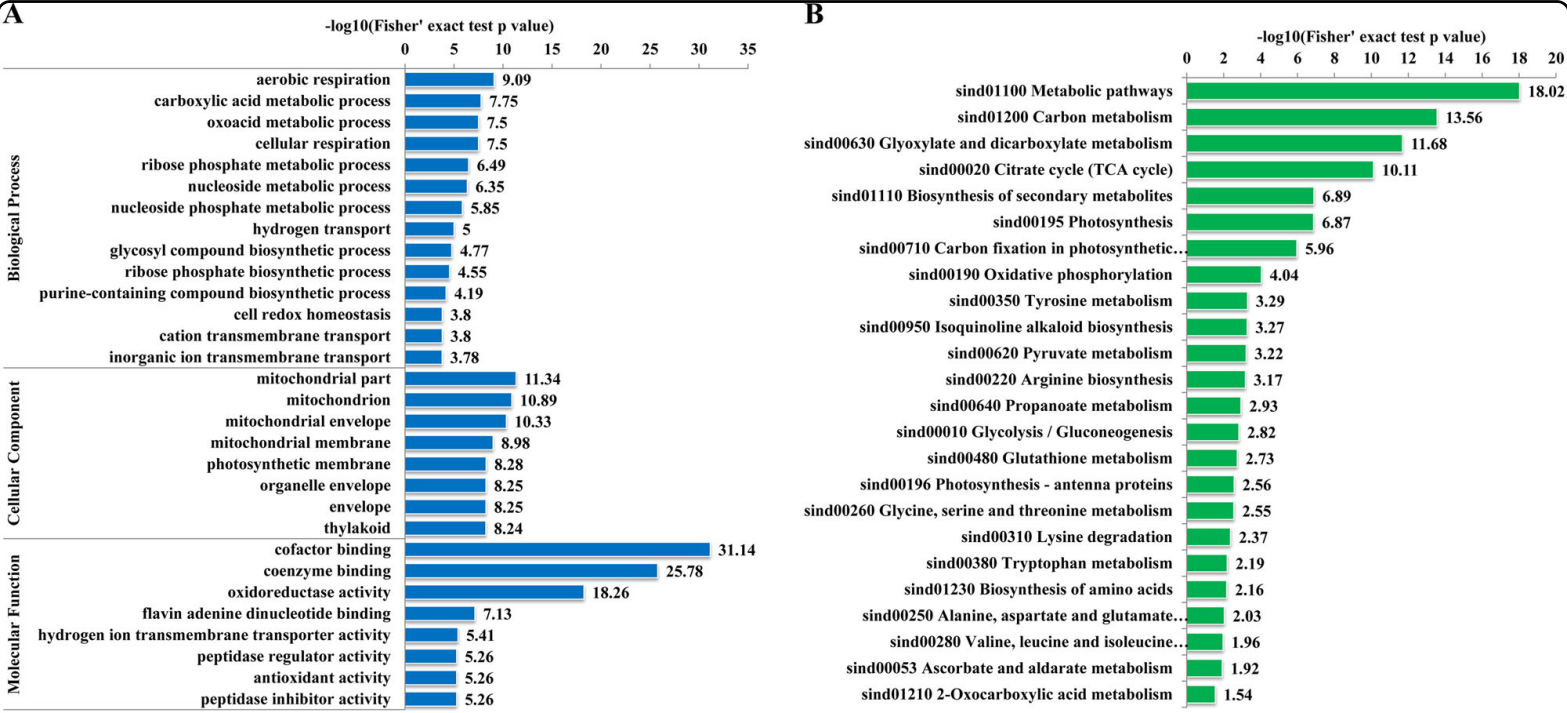
GO annotation and KEGG pathway based enrichment analysis. Enrichment analyses of the identified lysine succinylated proteins in the GO annotation (**a**) and KEGG pathway (**b**) categories.

As shown in Fig. 4a, in the biological process ontology, the significantly enriched GO terms were mainly related to energy production, material metabolism, and biosynthesis processes. Aerobic respiration, carboxylic acid metabolic process, oxoacid metabolism process, and cellular respiration were the top four significantly enriched biological processes, implying that the glycometabolism and respiration biological processes may be influenced by lysine succinylation. Several ribose phosphate, nucleoside and nucleoside phosphate metabolism or biosynthesis-related terms were also highly enriched. In the category cellular component, the top four significantly enriched GO terms were all related to mitochondria, suggesting that mitochondrial metabolism-related enzymes may be the targets and substrates of lysine succinylation modifications. In addition, two terms directly related to photosynthesis components were significantly enriched, namely, photosynthesis membrane and thylakoid. Regarding the molecular function category, cofactor binding, coenzyme binding, and oxidoreductase activity were the top three enriched terms.

By KEGG pathway enrichment analysis, we identified a total of 25 significantly enriched pathways (Fig. 4b and Supporting Table S3). Consistent with the GO enrichment analysis, the metabolic pathway was the most significantly enriched pathway, suggesting the regulatory role of succinylation in diverse material metabolism. Several energy production- and glycometabolism-related pathways, including carbon metabolism, the TCA cycle, oxidative phosphorylation, pyruvate metabolism, and glycolysis/gluconeogenesis, were highly enriched. Furthermore, we noticed that three photosynthesis-related pathways, photosynthesis, carbon fixation in photosynthesis, and photosynthesis-antenna proteins, were significantly enriched, implicating the underlying regulatory roles of lysine succinylation in patchouli plant photosynthesis. The majority of the remaining dramatically enriched pathways were related to the metabolism or biosynthesis of diverse secondary metabolites, such as glyoxylate, dicarboxylate, isoquinoline alkaloid, propanoate, glutathione, ascorbate and various amino acids (tyrosine, arginine, glycine, serine, threonine, lysine, tryptophan, alanine, aspartate, glutamate, valine, leucine and isoleucine). These markedly enriched diverse amino acid metabolism-related pathways were a noticeable phenomenon.

Domain analysis indicated that both of the top two significantly enriched domains were related to ATPase subunit structure (Supporting Figure S2). Moreover, ATP synthase and ATP-citrate lyase structure-related domains were also observed, which was consistent with the significantly enriched energy metabolism-related GO terms and KEGG pathways (Fig. 4).

### Protein–protein interaction (PPI) network construction

**Fig. 5:**
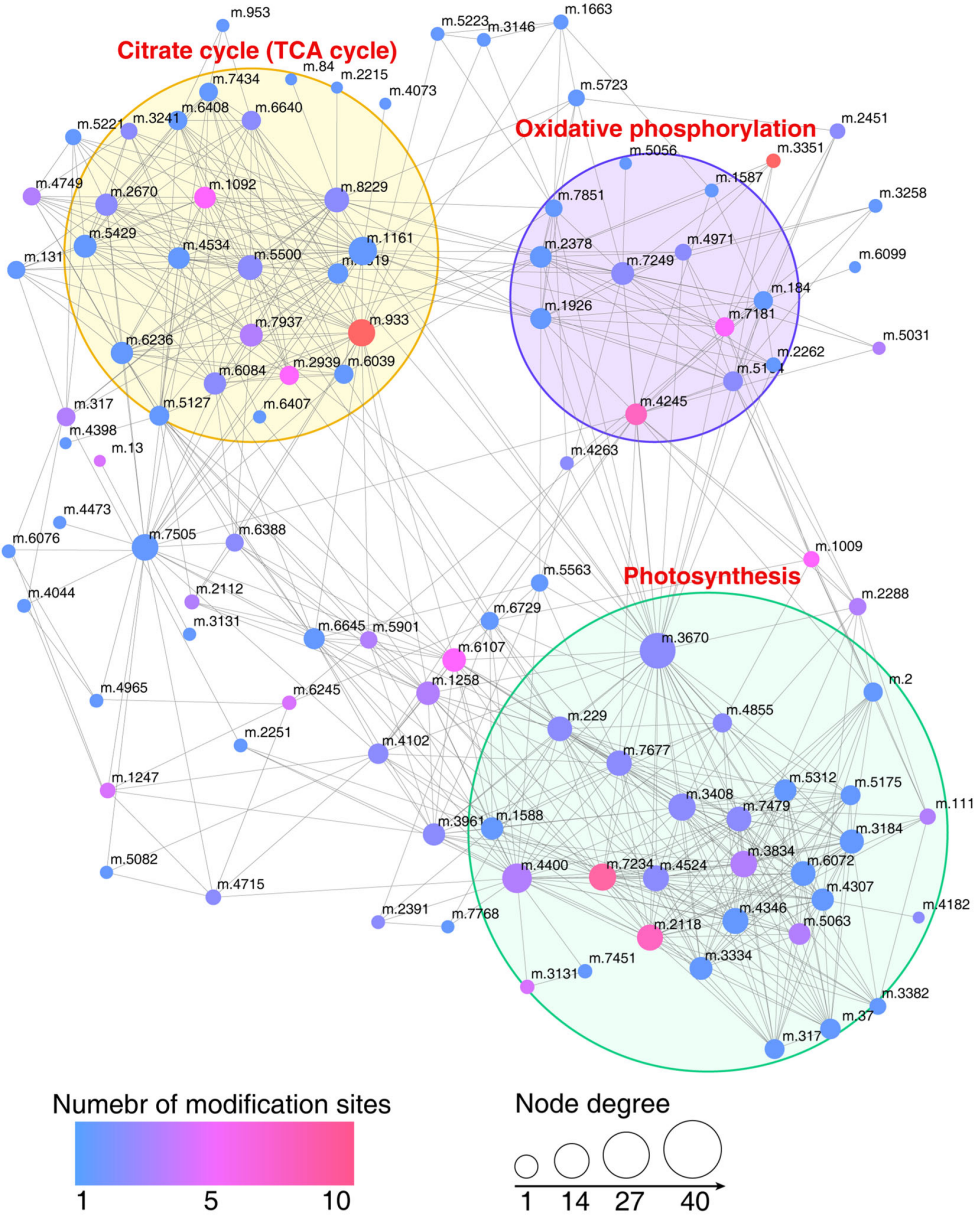
PPI network analysis of the succinylated proteins. The circle size represents mode degree and the color represents succinylation site number on the nodes. The node codes in the network correspond to the gene ID in the supporting information Table S4.

To investigate the interactions among various succinylated proteins and their involvement in diverse interacting pathways, we carried out protein interaction network (PPI) analysis with all the identified succinylation proteins. In total, 106 proteins carrying various succinylation sites were matched to the PPI network (Fig. 5, Supporting Table S4). The majority of the succinylated proteins were clustered into three highly interconnected networks, namely, citrate cycle (TCA cycle), oxidative phosphorylation and photosynthesis, suggesting the role of protein succinylation in modulating these biological processes. In addition, we noticed that the three internetworks were linked to each other through some succinylated proteins, implying that crosslinks were present among these biological processes and that succinylation may regulate these crosslinks.

The node degree is a key parameter to assess the importance and correlation of proteins in the network. The computed degree information for all succinylated proteins can be seen in Supporting Table S4, and the result showed that there were 20 succinylated proteins with a degree over 40 in the network, among which Cytochrome b6-f complex iron-sulfur subunit, chloroplastic had the highest degree. Moreover, some important nodes with a relatively higher degree displayed more than one succinylation site in the network, such as Dihydrolipoyl dehydrogenase 1, mitochondrial (12 sites), ADP, ATP carrier protein 1, mitochondrial (10 sites), Oxygen-evolving enhancer protein 3-1, chloroplastic (8 sites) and oxygen-evolving enhancer protein 1, chloroplastic (7 sites). The constructed PPI network information indicated that lysine succinylation was relatively active in the TCA cycle, oxidative phosphorylation and photosynthesis processes, and participated in protein–protein interactions that likely influenced the coordination of these processes in this traditional Chinese medicine plant.

## Discussion

In the present study, with an immunoprecipitation affinity enrichment technique combined with advanced mass spectrometry-based proteomics, we first obtained data of the lysine succinylome in patchouli plants. A previous study on plant succinylomes was mostly concentrated on Gramineae plants, among which the model plant rice has the most succinylation data available (2593 succinylation sites in 1024 proteins)^8,16–18^. In some horticultural species, including strawberry, tomato, tea, *Taxus* and *D. officinale*, global succinylation analysis has also been performed (Supporting Table S2), and varying succinylation sites and succinylation proteins have been identified^19–23^. Among medicinal herbs, only *D. officinale* has been studied, and 314 succinylation sites corresponding to 207 proteins have been reported in this herb^21^. In our study, a total of 1097 succinylation sites corresponding to 493 proteins were identified in patchouli plants, among which 466 succinylation sites corresponding to 241 proteins were repeatedly identified in three repeated experiments (Fig. 1b and Supporting Table S1). Obviously, there is some variation in succinylation site numbers and succinylation protein numbers in different reported plant species, which may be related to the differential intrinsic succinylation levels of proteins in various plant species. In addition, variation in plant growth stages, tissues selected for experiments and culture conditions may also contribute to this difference. Our study expanded the species and scope of lysine succinylation in plants, especially in medicinal plants. It may help uncover the potential important role of lysine succinylation in herb growth, development, and physiology.

To demonstrate the nature of the succinylated lysines in patchouli plants, motif and heat map analyses were conducted, and four consensus sequence motifs were obtained (Fig. 2a). Compared with the conserved motifs in other plant species, the motifs G*********Ksuc and V Ksuc were also significantly enriched in the succinylated peptides in rice^8,16^, suggesting that different plant species may share some common conserved motifs around succinylated lysine sites. In addition, two new conserved motifs, namely, Ksuc*I and Ksuc*****G, were identified in patchouli plants. It seemed that succinylation was preferred at lysine residues adjacent to residues bearing relatively small side chain groups, such as G and V. The overrepresented G in the −10, +3 and +6 positions, alanine A in the −9, −5 and −3 positions, and valine V in the −1 position in the heat map analysis further confirmed this concept. Moreover, in the succinylome analysis of silkworm and *Candida albicans*, the significantly enriched conserved motifs were all G or A-containing motifs. Furthermore, G and A were overrepresented in the heat map analysis of the amino acid compositions surrounding the succinylated lysine sites^9,29^. Succinylation introduces a relatively larger structural moiety to the lysine sites than acetylation and methylation, whose steric hindrance effect would be more significant^6^. We speculate that amino acids with small side chain groups may be more compatible with succinyl groups because their steric hindrance effects are relatively weak. Thus G and A were overrepresented surrounding the succinylated lysine sites.

Functional characterization and subcellular location analysis of lysine succinylated proteins in patchouli plants indicated that protein succinylation was involved in various metabolic and biological processes and occurred in diverse cellular compartments (Fig. 2), which is consistent with previous studies in other horticulture plant species (Supporting Fig. S1) and some model plants, such as rice and *Brachypodium distachyon* L.^16,18^. By comparing the succinylome data from this study and in studies on other species (Supporting Fig. S1) at the functional and subcellular location levels, we determined that tissue and organ differences may result in relatively higher variation in succinylation protein functions and subcellular distributions than plant species differences. In addition, the same tissues and organs (leaves) among various plant species showed similar succinylation protein function and subcellular location distribution, which is an interesting phenomenon.

**Fig. 6:**
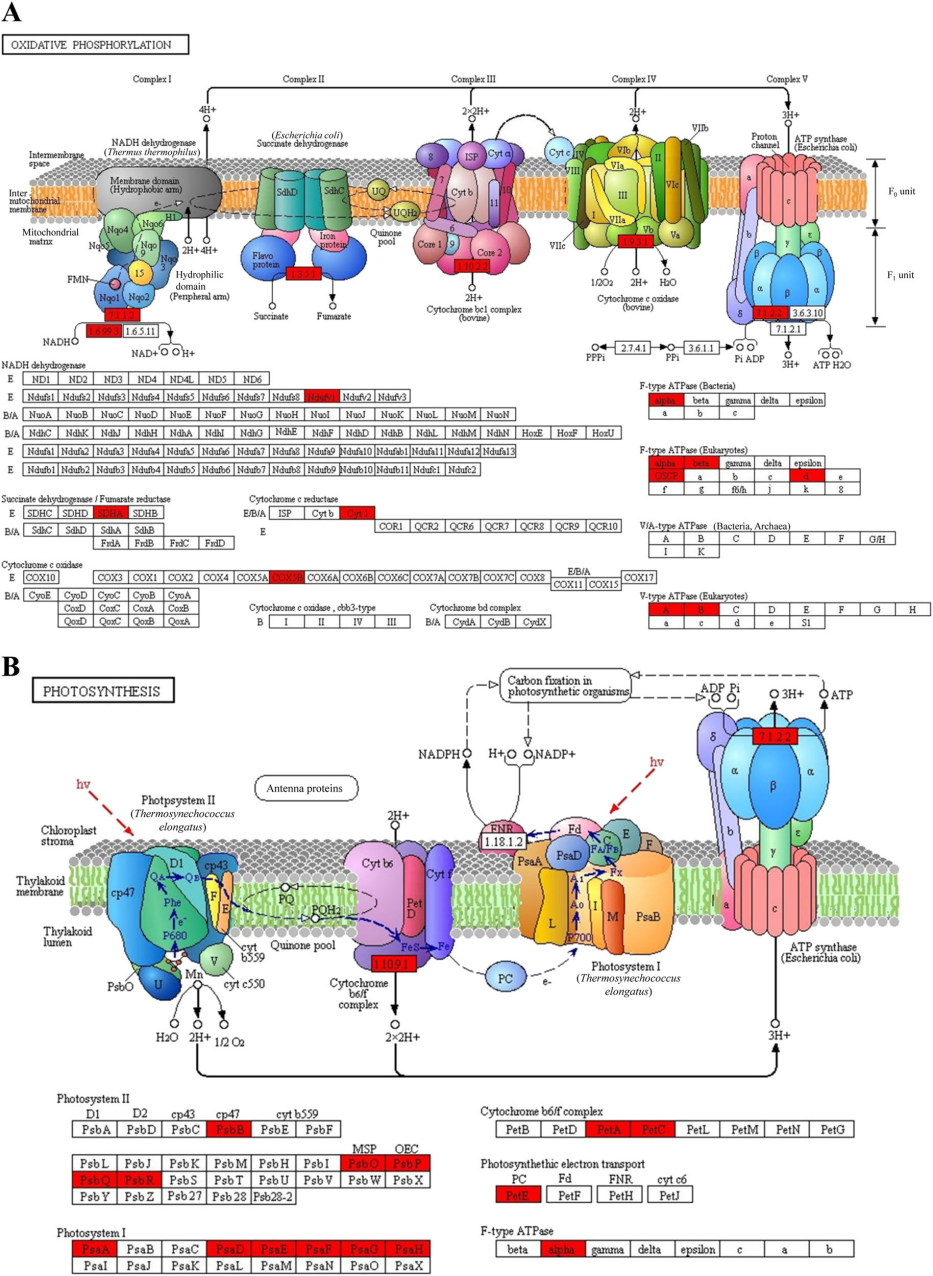
Representative significantly enriched KEGG pathways. **a** Oxidative phosphorylation and **b** photosynthesis. The succinylated proteins are labeled in red.

The TCA cycle and oxidative phosphorylation are critical in living organisms because they are major energy production processes^30^. In addition, the TCA cycle results in various substrates and intermediates for diverse primary metabolism and secondary metabolism pathways^31^. In our study, KEGG enrichment (Fig. 4b) and PPI network analyses (Fig. 5) indicated that many TCA cycle-related enzymes and oxidative phosphorylation-related protein complexes were succinylated in patchouli plants, especially during the oxidative phosphorylation process, and all five major protein complexes in the photosynthetic electron transfer chain underwent succinylation at some subunits (Fig. 6a).

Previous studies in bacteria (*E. coli*), yeast (*S. cerevisiae*), human cells and mouse liver tissue found lysine succinylation on TCA cycle-related enzymes and revealed the potential regulatory role of lysine succinylation in the TCA cycle^7^. Consistent with this previous study, 15 protein components or subunits belonging to TCA cycle-related enzymes or enzyme complexes were succinylation modified succinylated with variation in succinylation site number in patchouli plants (Supporting Table S3), suggesting that lysine succinylation may participate in TCA cycle regulation in this herb. Notably, some enzymes and enzyme complexes involved in the TCA cycle were succinylated at different components or subunits, such as in the pyruvate dehydrogenase complex, 3 sites in the dihydrolipoyllysine-residue acetyltransferase, 12 sites in dihydrolipoyl dehydrogenase 1 and 6 sites at pyruvate dehydrogenase E1. In addition, a similar phenomenon was observed in the pyruvate dehydrogenase complex in rice and in the *Brachypodium distachyon* L. complex^8,18^. We infer that the succinylation on these subunits or components possibly influenced the gathering and/or assembly of these enzymes or complexes and subsequently affected overall enzyme activity. However, more biochemical experiments are necessary to verify this assumption.

Oxidative phosphorylation results in ATP production through aerobic respiration in higher animals and higher plants are of vital importance for biological activities. The various lysine succinylation sites at the submits of complex I (NADH dehydrogenase), complex II (succinate dehydrogenase), complex III (cytochrome bc1 complex), complex IV (cytochrome c oxidase) and complex V (ATP synthase) may regulate the protein–protein interaction among these subunits and further influence ATP production, as a significantly enriched internetwork was obtained in the PPI analysis, and several proteins with a relatively high degree exhibited multisite succinylation (Fig. 5). Previous studies in other plant species, including rice, *Brachypodium distachyon* L. and strawberry, also reported succinylation on mitochondrial electron transport/ATP synthesis metabolism-related proteins and the ATP synthase^8,18,19^. Our study demonstrated lysine succinylation on other electron transport chain complexes and expanded the study of succinylation-regulated oxidative phosphorylation.

Photosynthesis in chloroplasts is an important biochemical reaction in plants because it converts light energy into chemical energy to support plant life. Previous global lysine succinylation analyses in *Taxus*, tea plant, *Brachypodium distachyon* L. and rice identified diverse photosynthesis-related succinylated sites and proteins and showed the potential functional regulatory role of lysine succinylation in some photosynthesis-related proteins^16,18,22,23^. Protein succinylation may be a conserved approach in photosynthesis regulation among different plant species.

Consistent with previous studies, our study of patchouli plants also identified many photosynthesis-related lysine succinylated proteins. Chloroplast-localized proteins with various succinylated sites accounted for the largest group of proteins in the subcellular location analysis (43%, Fig. 3d). Moreover, three photosynthesis-related pathways, photosynthesis, photosynthesis-antenna proteins and carbon fixation in photosynthetic organisms, were significantly enriched in the KEGG pathway analysis (Fig. 4b, Supporting Table S3), implying that both the light reaction and dark reaction may be influenced by protein succinylation. The PPI analysis further validated this hypothesis because a significantly enriched photosynthesis-related subnetwork was obtained in the contrasting network (Fig. 5). In the light reaction, chlorophyll a/b-binding protein (light-harvesting complex, LHC) captures light. Then, water is split by an oxygen-evolving complex (OEC) through the light-induced oxidation of water, resulting in the generation of molecular oxygen, reduced plastoquinone, and a transmembrane proton gradient^32^. As shown in Fig. 6b and Supporting Table S3, almost all major complexes of the photosynthetic electron transport system in light reactions, including photosystem II, photosystem I, cytochrome b6f complex, and ATP synthase, were succinylated at some subunits or constitute proteins, such as the chlorophyll a/b-binding protein, photosystem I reaction center subunits (II, III, VI, V), photosystem II reaction center subunit CP47 reaction center protein, oxygen-evolving enhancer protein 1, oxygen-evolving enhancer protein 2, cytochrome b6-f complex iron-sulfur subunit, plastocyanin and ATP synthase subunit alpha. Electron and hydrogen proton transport is an active event in the light reaction, which could be influenced by the charge states of the involved proteins. We hypothesize that the molecular mechanisms of succinylation-regulated light reaction processes may be related to succinylation-induced charge state alterations in addition to succinylation-induced steric hindrance effects^6^.

Carbon fixation in the Calvin–Benson cycle is the primary carbon assimilation pathway in plants^33^. In our study, many Calvin–Benson cycle-related enzymes harbored succinylation sites, such as ribulose bisphosphate carboxylase (Rubisco), phosphoglycerate kinase, fructose-bisphosphate aldolase, glyceraldehyde-3-phosphate dehydrogenase, sedoheptulose-1,7-bisphosphatase, and glutamate-glyoxylate aminotransferase (Supporting Table S3). A previous study also reported lysine succinylation in carbon fixation-related proteins, among which Rubisco exhibited the most succinylation sites^16–18,23^. Our study revealed a similar phenomenon and identified seven succinylation sites in Rubisco, of which five were in the Rubisco large subunit and two were in the Rubisco small subunit. The increased identification of succinylation sites in Rubisco may be related to the large size of this enzyme and the high abundance of Rubisco in plant leaf tissues. Rubisco, with a molecular weight of 560 kDa, is one of the largest enzymes in nature^33^. In addition, it is the most abundant leaf tissue-distributed protein, accounting for approximately half of the soluble protein in plant leaves^33^. Its large size is indicative of more potential lysine sites, which are the preferred sites of succinylation modification, while its high abundance is indicative of increased chances of interacting with enzymes or cofactors responsible for intracellular succinylation modifications^6,34^. Both factors resulted in more Rubisco-related peptides being enriched in immunoprecipitation affinity in the sample preparation stage. The identified succinylation sites in Rubisco and other carbon fixation-related enzymes indicate that succinylation is a crucial PTM in the photosynthetic carbon assimilation regulation in patchouli plants.

Amino acids are structural units of proteins in living organisms. In the pathway enrichment analyses, we noticed as many as ten amino acid metabolism pathways, especially amino acid biosynthesis-related pathways, were significantly enriched, and the metabolism of at least 13 amino acids was affected by these pathways (Fig. 4b and Supporting Table S3). In addition, some significantly enriched glycometabolism-related pathways, such as the TCA cycle, pyruvate metabolism, and glycolysis/gluconeogenesis, may also be involved in amino acid biosynthesis because these metabolism pathways produce substrates and intermediates for amino acid biosynthesis^30,31^. We propose that lysine succinylation may play a role in amino acid metabolism in patchouli plants. The mechanism of succinylation-regulated amino acid biosynthesis in patchouli plants may be related to lysine succinylation-altered activities of various enzymes in amino acid biosynthesis pathways. The obtained amino acid biosynthesis-related enzymes in the enriched pathways, such as glutamine synthetase, aspartate aminotransferase, serine hydroxymethyltransferase, and aminomethyltransferase, have validated this regulatory mechanism to some extent. A previous study in *Brachypodium distachyon* L. and rice also reported lysine succinylation upon amino acid biosynthesis and protein synthesis-related proteins, such as ribosomal subunits and aminoacyl-tRNA synthetase^16,18^. Our study expanded the understanding of lysine succinylation-regulated amino acid biosynthesis in plants.

By comparing other succinylomes in the aforementioned various species, we noticed that some proteins had succinylation modifications in various plant species, indicating reduced species preference, especially some critical enzymes and proteins in central metabolic and biological processes, such as pyruvate dehydrogenase, NADH dehydrogenase, ATP synthase, light-harvesting complex, Rubisco, and aminoacyl-tRNA synthetase. Succinylation modification may influence the activity and/or structure stability of these critical enzymes and proteins and further regulate diverse metabolic pathways and biological activities among various plant species. Various plant species may share some common mechanisms in succinylated-regulated metabolic processes and biological activities.

## Conclusion

In the present study, the global succinylation in patchouli plants, an important traditional Chinese medicine herb, was first identified. In total, 1097 succinylation sites corresponding to 493 proteins were identified, among which 466 succinylation sites corresponding to 241 proteins were repeatedly identified in three independent experiments. Our study expanded the scope of lysine succinylation in plants. Motif analysis of these succinylated peptides revealed four conserved sequence motifs. These succinylated proteins showed a wide subcellular location distribution, although the chloroplast and cytoplasm were the top two preferred cellular components. Functional characterization of these proteins with bioinformatic tools showed that lysine succinylation was involved in diverse biological processes and metabolic pathways. The TCA cycle and oxidative phosphorylation-related enzymes and protein complexes are preferred substrates of succinylation in patchouli plants. Photosynthesis processes, including light reactions and carbon fixation, may be regulated by lysine succinylation modifications. Amino acid biosynthesis may be adjusted by succinylation by affecting the activities of enzymes within amino acid metabolism pathways. Our work may serve as a useful resource for the functional demonstration of lysine succinylation in important medicinal herbs and a useful reference for succinylation studies in other medicinal plants.

## Materials and methods

### Reagents

The anti-succinyl lysine antibody was purchased from Cell Signal Technology (CST, Massachusetts, USA). All the other reagents unless otherwise stated were purchased from Sigma (St. Louis, USA).

### Plant materials

Patchouli plants *(P. cablin* (Blanco) Beth, Lamiaceae) were obtained from ‘Shizhen’ Mountain, Guangzhou University of Traditional Chinese Medicine (23.03°N, 113.23°E), Guangzhou, China. Cuttings of *P. cablin* were transplanted into flower pots and grown in a chamber at 22–25 °C with a 16/8 h photoperiod and 10,000 lux light intensity for 4 weeks. Leaf tissues from three individual plants were collected for three biological replicates. All samples were frozen and stored at −80 °C until use.

### Proteomic sample preparation, HPLC fractionation, and succinylated enrichment

Protein extraction was conducted according to a previous report with some modifications^35^. Briefly, the patchouli leaf tissues were fully ground in liquid nitrogen with a mortar and pestle for 10 min. The resulting powder was resuspended in ice-cold extraction buffer (250 mM sucrose, 10 mM EDTA, 50 mM Tris-HCl, pH 7.5, 1% Triton X-100, 1 mM PMSF, 1 mM DTT and histone deacetylase inhibitors). The extraction buffer was mixed with ice-cold Tris buffer phenol (pH 8.0) and shaken for 10 min. After centrifugation at 15,000 × *g* for 15 min, the phenolic phase was transferred to a new tube and precipitated with four volumes of 100 mM ammonium acetate in methanol at −20 °C overnight. Following centrifugation (30 min, 15,000 × *g*), the resulting pellet was rinsed twice with ice-cold acetone, vacuum-dried and stored at −80 °C.

In the following trypsin digestion, the dried pellets were dissolved in buffer (8 M urea, 10 mM DTT), and the protein concentration was quantified with a 2-D Quant kit (GE Healthcare, America) according to the manufacturer’s instructions. Subsequently, 10 mM DTT was added to the protein solution and incubated for 1 h at 37 °C. Then, alkylate with 20 mM iodoacetamide (IAA) was added and incubated for 45 min at room temperature in the dark. The protein solution was diluted with quintuple 100 mM NH_4_CO_3_. Finally, trypsin (Promega) was added to the sample (enzyme-to-substrate ratio 1:50) for 12 h at 37 °C, and the second digestion was conducted to complete digestion^36^. Following trypsin digestion, the peptide was desalted with a Strata X C18 SPE column (Phenomenex) and vacuum-dried.

The tryptic peptides were fractionated through reverse-phase HPLC with a C18 column (Phenomenex Gemini NX-C18 RP column, 5 μm particles, 10 × 250 mm size) in Shimadzu LC20AD. The mobile phase buffer was solvent A (100% H_2_O, 5 mM NH_4_OH) and solvent B (80% ACN, 5 mM NH_4_OH). The LC gradient was run with 5–30% solvent B for 13 min and 30–80% solvent B for 7 min at a flow rate of 4.8 ml/min to generate 96 fractions at 0.25 min/fraction. The resulting peptides were combined into 4 fractions for succinylome analysis.

The immunoprecipitation process was carried out to enrich the succinylated peptides^37^. First, the peptides were resuspended in NETN buffer (100 mM NaCl, 1 mM EDTA, 50 mM Tris-HCl, 0.5% NP-40, pH 8.0). Then, the peptides were incubated with pan-anti-succinyllysine agarose beads at 4 °C for 15 h with gentle shaking at a ratio of 15 μL beads/mg proteins. After incubation, the beads were washed four times with NETN buffer and twice with purified water. Finally, the captured peptides were eluted with 0.1% trifluoroacetic acid, and the peptides were vacuumed.

### LC-MS/MS analysis

LC-MS/MS analysis was carried out following the previously reported method with some modifications^22^. The dissolved peptides in buffer A (0.1% FA, 100% H_2_O) after centrifugation were injected into a reversed-phase analytical column (Thermo Acclaim PepMap RSLC C18 column, 2 μm, 75 μm × 50 mm) on an EASY-nLC UPLC system (Ultimate RSLCnano 3000). The elution linear gradient to solvent B (0.1% FA in 90% ACN) was 5% to 20% for 40 min, 20% to 35% for 8 min, increased to 80% within 5 min and then held at 80% for the last 5 min at a constant flow rate of 250 nl/min.

The eluted peptides were subjected to a NanoSpray Ionization (NSI) source followed by MS/MS in Q Exactive (Thermo Scientific) coupled online to the UPLC in data-dependent acquisition (DDA) mode. The precursor ions were detected at a resolution of 60,000, and the m/z mass scan range was 350–1800 Da. For MS/MS analysis, the NCE was set as 26 in HCD mode, and the resolution was 30,000. One MS scan followed by 15 MS/MS scans was applied for the top 15 precursor ion acquisitions above a threshold ion count of 1E5 in the MS survey scan with 6.0s dynamic exclusion. The applied electrospray voltage was set to 2.0 kV, and 5E4 ions were accumulated for the generation of MS/MS. LC-MS/MS analysis was performed blindly by Micrometer Biotech Company (Hangzhou, China).

### Database searching

The MS data were searched against the *Pogostemon cablin* (Blanco) Benth. transcriptome data (NCBI BioProject: PRJNA528262; SRA: SRR8761881 and SRR8761882). The transcriptome data were concatenated using the reverse decoy database using the MaxQuant search engine (version 1.4.1.2). The database search parameters were as follows. Trypsin/P was specified as the cleavage enzyme. Missing cleavage sites within four sites were allowed. Carbamidomethylation on Cys was defined as a fixed modification, while oxidation on Met, succinylation on Lys and succinylation on the N-terminus were set as variable modifications. For the precursor ions, mass error was tolerated within 10 ppm. For fragment ions, 0.02 Da was accepted. The false discovery rate was specified at 1%. The length of the shortest peptide for database search was set to seven amino acid residues. All of the other parameters used in MaxQuant were default settings.

### Bioinformatic analysis

Motif-x software was used for the motif analysis. The software computes the probability analysis of the amino acids are overrepresented or underrepresented at the positions adjacent to succinylation sites (−10 to +10 position)^38^. The Gene Ontology (GO) database was used to annotate all the identified succinylated proteins in three ontologies (biological process, molecular function, and cellular compartment)^39^. The Kyoto Encyclopedia of Genes and Genomes (KEGG) database was used to annotate protein pathway^40^. WoLF PSORT (subcellular localization predication software) was selected to predict the subcellular localization of the succinylated proteins^41^. The tool for enrichment analysis was DAVID, and an adjusted *p*-value less than 0.05 was chosen as the cut-off criterion^42^. For protein–protein interaction analysis, the Search Tool for the Retrieval of Interacting Genes/Proteins (STRING) database^43^ and Cytoscape software (version 3.0.1)^44^ were used.

## Supplementary information

Supporting information

## References

[1] Mann M, Jensen ON (2003). Proteomic analysis of post-translational modifications. Nat. Biotechnol..

[2] Nørregaard Jensen O (2004). Modification-specific proteomics: characterization of post-translational modifications by mass spectrometry. Curr. Opin. Chem. Biol..

[3] Fan J, Krautkramer KA, Feldman JL, Denu JM (2015). Metabolic regulation of histone post-translational modifications. ACS Chem. Biol..

[4] Boutureira O, Bernardes GJL (2015). Advances in chemical protein modification. Chem. Rev..

[5] Khoury GA, Baliban RC, Floudas CA (2011). Proteome-wide post-translational modification statistics: frequency analysis and curation of the swiss-prot database. Sci. Rep..

[6] Zhang Z (2011). Identification of lysine succinylation as a new post-translational modification. Nat. Chem. Biol..

[7] Weinert BT (2013). Lysine succinylation is a frequently occurring modification in prokaryotes and eukaryotes and extensively overlaps with acetylation. Cell Rep..

[8] He D (2016). Global proteome analyses of lysine acetylation and succinylation reveal the widespread involvement of both modification in metabolism in the embryo of germinating rice seed. J. Proteome Res..

[9] Zheng H (2016). Systematic Analysis of the Lysine Succinylome in Candida albicans. J. Proteome Res.

[10] Ren S (2018). Lysine succinylation contributes to aflatoxin production and pathogenicity in aspergillus flavus. Mol. Cell Proteom..

[11] Xu X (2017). The first succinylome profile of Trichophyton rubrum reveals lysine succinylation on proteins involved in various key cellular processes. BMC Genomics.

[12] Park J (2013). SIRT5-mediated lysine desuccinylation impacts diverse metabolic pathways. Mol. Cell.

[13] Cheng Y, Hou T, Ping J, Chen G, Chen J (2016). Quantitative succinylome analysis in the liver of non-alcoholic fatty liver disease rat model. Proteome Sci..

[14] Zhang N (2018). Quantitative global proteome and lysine succinylome analyses reveal the effects of energy metabolism in renal cell carcinoma. Proteomics.

[15] Song Y (2017). Quantitative global proteome and lysine succinylome analyses provide insights into metabolic regulation and lymph node metastasis in gastric cancer. Sci. Rep..

[16] Zhou H (2018). Oxidative stress-triggered interactions between the succinyl- and acetyl-proteomes of rice leaves. Plant Cell Environ..

[17] Zhang Y (2017). Global analysis of protein lysine succinylation profiles in common wheat. BMC Genomics.

[18] Zhen S (2016). First comprehensive proteome analyses of lysine acetylation and succinylation in seedling leaves of *Brachypodium distachyon* L. Sci. Rep..

[19] Fang X (2018). Systematic identification and analysis of lysine succinylation in strawberry stigmata. J. Agric Food Chem..

[20] Jin W, Wu F (2016). Proteome-wide identification of lysine succinylation in the proteins of tomato (Solanum lycopersicum). PloS One.

[21] Feng S (2017). Succinyl-proteome profiling of Dendrobium officinale, an important traditional Chinese orchid herb, revealed involvement of succinylation in the glycolysis pathway. BMC Genomics.

[22] Shen C (2016). Succinyl-proteome profiling of a high taxol containing hybrid Taxus species (Taxus × media) revealed involvement of succinylation in multiple metabolic pathways. Sci. Rep..

[23] Xu YX (2017). Quantitative succinyl-proteome profiling of Camellia sinensis cv. ‘Anji Baicha’ during periodic albinism. Sci. Rep..

[24] Swamy MK, Sinniah UR (2016). Patchouli (Pogostemon cablin Benth.): botany, agrotechnology and biotechnological aspects. Ind. Crops Products.

[25] He Y (2018). Building an octaploid genome and transcriptome of the medicinal plant Pogostemon cablin from Lamiales. Sci. Data.

[26] Zhang J (2018). Comparative proteomic analysis of Pogostemon cablin leaves after continuous cropping. Protein Expr. Purif..

[27] He Y, Deng C, Xiong L, Qin S, Peng C (2016). Transcriptome sequencing provides insights into the metabolic pathways of patchouli alcohol and pogostone in Pogostemon cablin (Blanco) Benth. *Genes &*. Genomics.

[28] Deutsch EW (2017). The ProteomeXchange consortium in 2017: supporting the cultural change in proteomics public data deposition. Nucleic acids Res..

[29] Chen J (2018). Systematic identification of mitochondrial lysine succinylome in silkworm (Bombyx mori) midgut during the larval gluttonous stage. J. Proteom..

[30] Fernie AR, Carrari F, Sweetlove LJ (2004). Respiratory metabolism: glycolysis, the TCA cycle and mitochondrial electron transport. Curr. Opin. Plant Biol..

[31] Sweetlove LJ, Beard KFM, Nunes-Nesi A, Fernie AR, Ratcliffe RG (2010). Not just a circle: flux modes in the plant TCA cycle. Trends Plant Sci..

[32] Zhang H (2012). Mechanisms of plant salt response: insights from proteomics. J. Proteome Res..

[33] Spreitzer RJ, Salvucci ME (2002). RUBISCO: structure, regulatory interactions, and possibilities for a better enzyme. Annu. Rev. Plant Biol..

[34] Wang, Y., Zhang, J., Li, B. & He, Q.-Y. Advances of proteomics in novel PTM discovery: applications in cancer therapy. *Small Methods* **3**, 1900041 (2019).

[35] Berger SL (2007). The complex language of chromatin regulation during transcription. Nature.

[36] Chen Y (2007). Lysine propionylation and butyrylation are novel post-translational modifications in histones. Mol. Cell. Proteom..

[37] Hershberger KA (2018). Ablation of Sirtuin5 in the postnatal mouse heart results in protein succinylation and normal survival in response to chronic pressure overload. J. Biol. Chem..

[38] Chou, M. F. & Schwartz, D. Biological sequence motif discovery using motif-x. *Curr. Protoc. Bioinformatics* **35**, Unit 13.15–24 (2011). 10.1002/0471250953.bi1315s35 21901740

[39] Hulsegge I, Kommadath A, Smits MA (2009). Globaltest and GOEAST: two different approaches for Gene Ontology analysis. BMC Proc..

[40] Moriya Y, Itoh M, Okuda S, Yoshizawa AC, Kanehisa M (2007). KAAS: an automatic genome annotation and pathway reconstruction server. Nucleic Acids Res..

[41] Horton P (2007). WoLF PSORT: protein localization predictor. Nucleic acids Res..

[42] Bradel-Tretheway BG (2011). Comprehensive proteomic analysis of influenza virus polymerase complex reveals a novel association with mitochondrial proteins and RNA polymerase accessory factors. J. Virol..

[43] Szklarczyk D (2011). The STRING database in 2011: functional interaction networks of proteins, globally integrated and scored. Nucleic Acids Res..

[44] Shannon P (2003). Cytoscape: a software environment for integrated models of biomolecular interaction networks. Genome Res..

