# Supplementary material for: Global analysis of lysine succinylation in patchouli plant leaves
Source: Hortic Res. 2019 Dec 1;6:133. doi: 10.1038/s41438-019-0216-5 (PMC6885049; doi:10.1038/s41438-019-0216-5)
Supplement: Supplementary file 1 — Supporting information [file 41438_2019_216_MOESM1_ESM.docx]

**Supporting information**

**Supporting Figures**

**
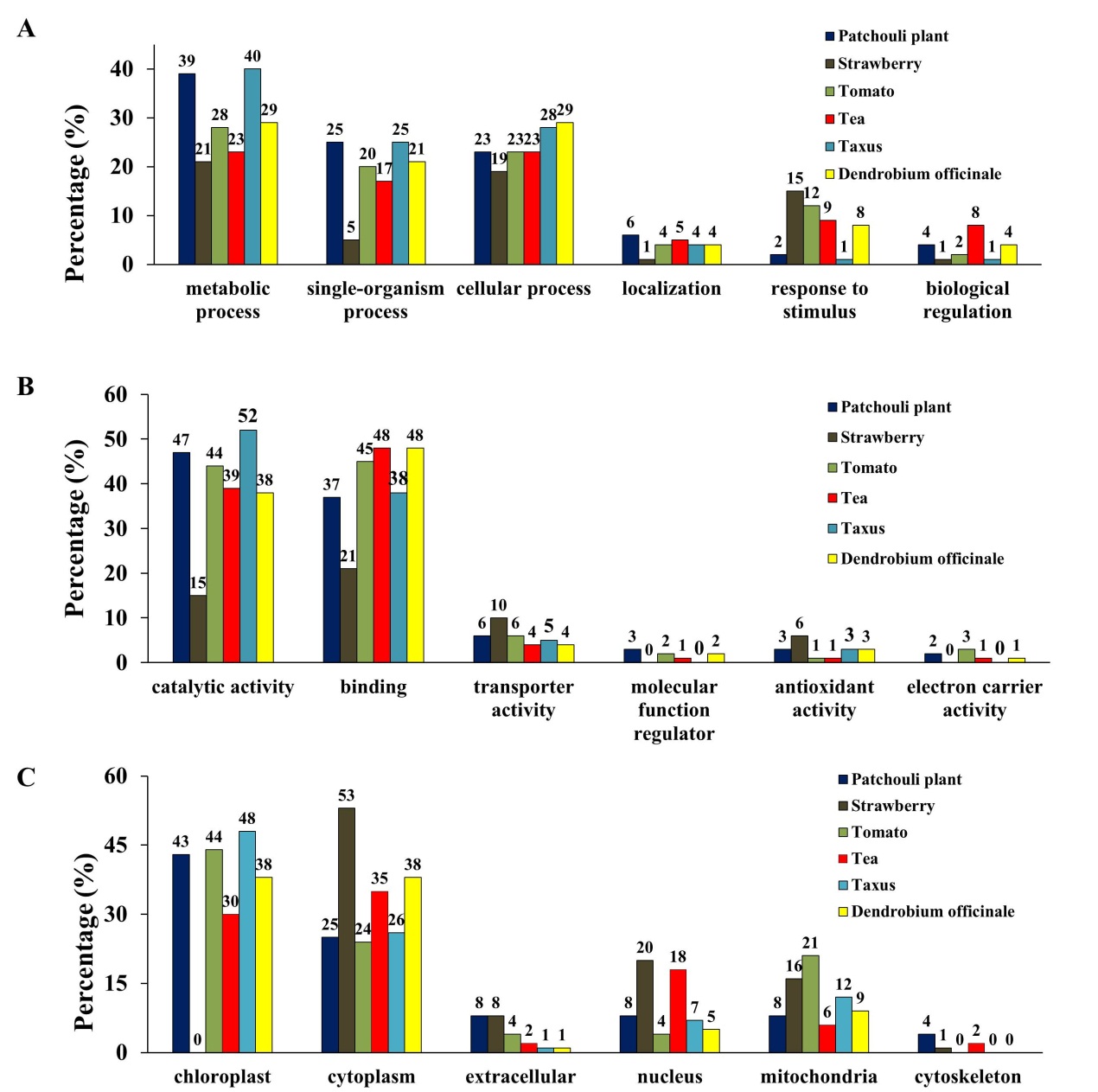
**

**Figure S1** The comparision of functional classification on biological processes level (A) and molecular function level (B) and subcellular location distribution (C) of the succinylated proteins between patchouli plant and various reported other horticulture plant speies. The data of strawberry, tomato, and *Dendrobium officinale* were cited from the published papers*.* For *taxus*, the data of subcellular location were cited form the paper while the data of function classification was obtained through analyzing the related supplementary file in the corresponding paper with the methods descripted in our manuscrtipt. For tea, both were obtained through analyzing the related supplementary file in the corresponding published paper as the original article hasn’t shown this data.

**
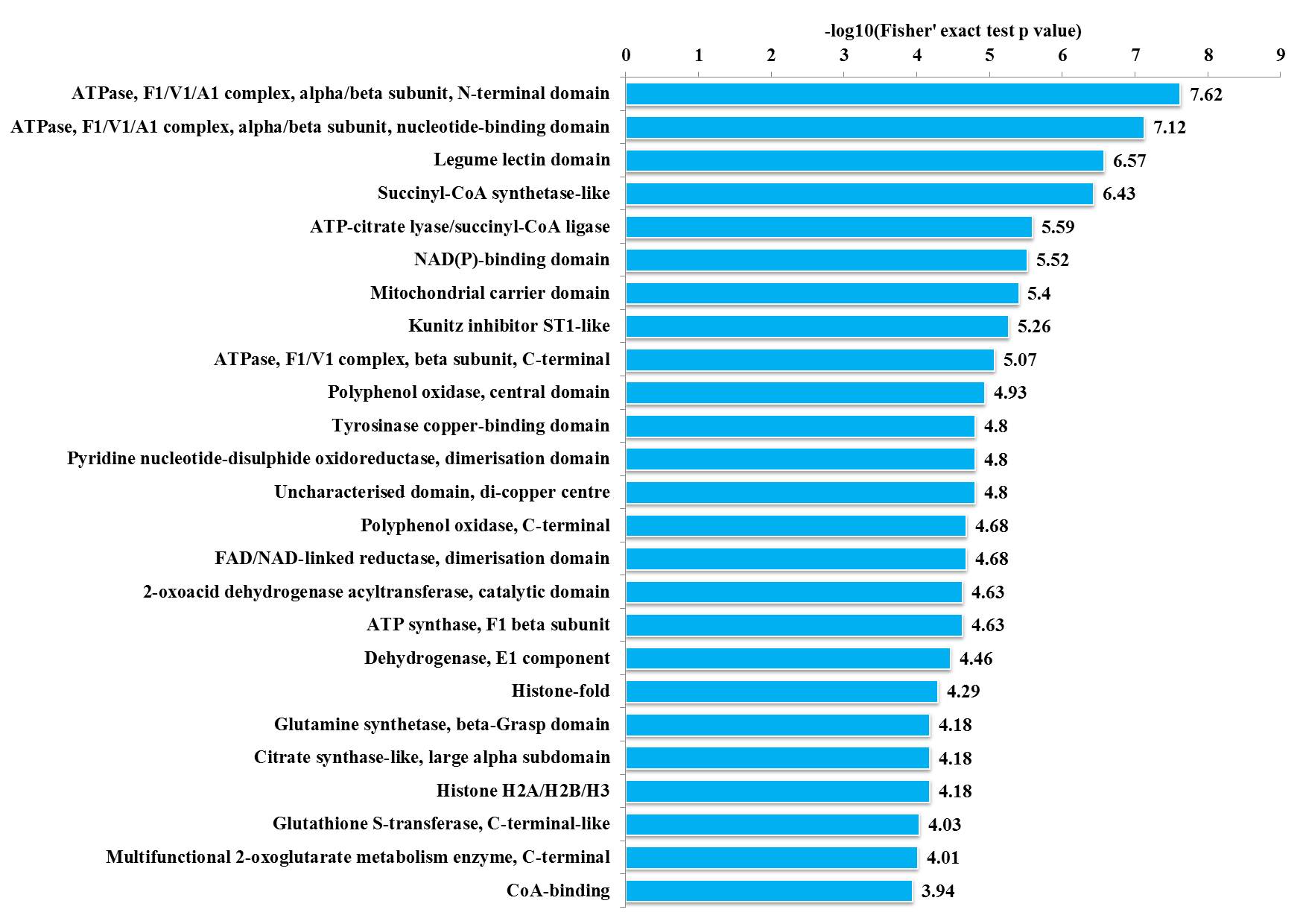
**

**Figure S2** Enrichment analysis of all the identified lysine succinylated proteins based on domain.

**Supporting Tables**

**Table S1** Summary of all the identified lysine succinylation (Ksuc) sites and proteins in patchouli plant leaves within three independent experiments (Page3-39).

**Table S2** The identified succinylated sites and protein in patchouli plant and in other horticultural species (Page40).

**Table S3** The detailed information of the significantly enriched KEGG pathways (Page41-91).

**Table S4** The detailed information of the succinylated proteins in the protein-protein interaction network (Page89-92).

**Table S1** Summary of all the identified lysine succinylation (Ksuc) sites and proteins in patchouli plant leaves within three independent experiments

| **Protein accession** | **Ksuc Position** | **Protein description** | **Repeat identification** |
| --- | --- | --- | --- |
| 7973_0.path0_m.3916 | 289 | (S)-ureidoglycine aminohydrolase | 3 |
| 7828_0.path0_m.3699 | 218 | 12-oxophytodienoate reductase 3 | 1 |
| 8384_0.path0_m.4387 | 48 | 14-3-3-like protein 16R | 1 |
| 8384_0.path0_m.4387 | 81 | 14-3-3-like protein 16R | 2 |
| 7567_0.path0_m.3332 | 99 | 14-3-3-like protein A | 2 |
| 7567_0.path0_m.3332 | 13 | 14-3-3-like protein A | 1 |
| i1_LQ_c7379_f1p0_1442_m.2478 | 292 | 1-aminocyclopropane-1-carboxylate oxidase 3 | 3 |
| 4008_0.path0_m.6859 | 209 | 2,3-bisphosphoglycerate-independent phosphoglycerate mutase | 2 |
| 4008_0.path0_m.6859 | 476 | 2,3-bisphosphoglycerate-independent phosphoglycerate mutase | 1 |
| 11861_0.path0_m.2371 | 237 | 20 kDa chaperonin, chloroplastic | 1 |
| i0_HQ_c21487_f2p2_711_m.4122 | 168 | 20 kDa chaperonin, chloroplastic | 1 |
| 11861_0.path0_m.2371 | 230 | 20 kDa chaperonin, chloroplastic | 3 |
| 11861_0.path0_m.2371 | 67 | 20 kDa chaperonin, chloroplastic | 3 |
| 11861_0.path0_m.2371 | 195 | 20 kDa chaperonin, chloroplastic | 2 |
| 8474_0.path0_m.4526 | 195 | 21 kDa seed protein | 3 |
| 8474_0.path0_m.4526 | 42 | 21 kDa seed protein | 3 |
| 8214_0.path0_m.4129 | 175 | 21 kDa seed protein | 2 |
| 8474_0.path0_m.4526 | 207 | 21 kDa seed protein | 3 |
| 9669_0.path0_m.6087 | 301 | 26S protease regulatory subunit 8 homolog B | 1 |
| 8576_0.path0_m.4715 | 260 | 2-Cys peroxiredoxin BAS1-like, chloroplastic | 2 |
| 8576_0.path0_m.4715 | 181 | 2-Cys peroxiredoxin BAS1-like, chloroplastic | 3 |
| 8576_0.path0_m.4715 | 202 | 2-Cys peroxiredoxin BAS1-like, chloroplastic | 2 |
| 8576_0.path0_m.4715 | 96 | 2-Cys peroxiredoxin BAS1-like, chloroplastic | 2 |
| 8576_0.path0_m.4715 | 177 | 2-Cys peroxiredoxin BAS1-like, chloroplastic | 3 |
| 10667_0.path0_m.939 | 46 | 2-methylene-furan-3-one reductase | 3 |
| 6522_0.path0_m.2251 | 191 | 2-methylene-furan-3-one reductase | 1 |
| 8194_0.path0_m.4253 | 120 | 2-methylene-furan-3-one reductase | 1 |
| 6522_0.path0_m.2251 | 333 | 2-methylene-furan-3-one reductase | 2 |
| 10667_0.path0_m.940 | 27 | 2-methylene-furan-3-one reductase | 1 |
| 10667_0.path0_m.940 | 113 | 2-methylene-furan-3-one reductase | 1 |
| 8194_0.path0_m.4253 | 212 | 2-methylene-furan-3-one reductase | 1 |
| 6522_0.path0_m.2251 | 118 | 2-methylene-furan-3-one reductase | 3 |
| 10667_0.path0_m.940 | 119 | 2-methylene-furan-3-one reductase | 1 |
| 1931_0.path2_m.3930 | 437 | 2-oxoglutarate dehydrogenase, mitochondrial | 3 |
| 1931_0.path2_m.3930 | 456 | 2-oxoglutarate dehydrogenase, mitochondrial | 2 |
| 2221_0.path0_m.4534 | 617 | 2-oxoglutarate dehydrogenase, mitochondrial | 2 |
| 1242_0.path10_m.2975 | 613 | 2-oxoglutarate dehydrogenase, mitochondrial | 3 |
| 1931_0.path2_m.3930 | 392 | 2-oxoglutarate dehydrogenase, mitochondrial | 3 |
| 2221_0.path0_m.4534 | 604 | 2-oxoglutarate dehydrogenase, mitochondrial | 3 |
| 1931_0.path2_m.3930 | 373 | 2-oxoglutarate dehydrogenase, mitochondrial | 3 |
| 1931_0.path2_m.3930 | 446 | 2-oxoglutarate dehydrogenase, mitochondrial | 3 |
| 1931_0.path2_m.3930 | 459 | 2-oxoglutarate dehydrogenase, mitochondrial | 3 |
| 1931_0.path2_m.3930 | 362 | 2-oxoglutarate dehydrogenase, mitochondrial | 3 |
| 1931_0.path2_m.3930 | 218 | 2-oxoglutarate dehydrogenase, mitochondrial | 3 |
| 1242_0.path10_m.2975 | 603 | 2-oxoglutarate dehydrogenase, mitochondrial | 3 |
| 1931_0.path2_m.3930 | 843 | 2-oxoglutarate dehydrogenase, mitochondrial | 3 |
| 2221_0.path0_m.4534 | 614 | 2-oxoglutarate dehydrogenase, mitochondrial | 2 |
| i1_LQ_c10413_f1p0_1112_m.6127 | 192 | 3-hydroxyacyl-[acyl-carrier-protein] dehydratase FabZ | 2 |
| i1_LQ_c14245_f1p3_1457_m.1128 | 279 | 3-hydroxyisobutyryl-CoA hydrolase-like protein 3, mitochondrial | 3 |
| 8586_0.path0_m.4733 | 230 | 3-ketoacyl-CoA thiolase 2, peroxisomal | 1 |
| 8586_0.path0_m.4733 | 239 | 3-ketoacyl-CoA thiolase 2, peroxisomal | 1 |
| i0_LQ_c196076_f1p0_817_m.5460 | 142 | 40S ribosomal protein S11 | 3 |
| i0_LQ_c196076_f1p0_817_m.5460 | 44 | 40S ribosomal protein S11 | 1 |
| i0_LQ_c13928_f1p3_562_m.4294 | 84 | 40S ribosomal protein S15a | 2 |
| i0_LQ_c13247_f5p2_772_m.4260 | 7 | 40S ribosomal protein S19-1 | 2 |
| i0_LQ_c13247_f5p2_772_m.4260 | 26 | 40S ribosomal protein S19-1 | 1 |
| i0_LQ_c9867_f1p8_794_m.5152 | 30 | 40S ribosomal protein S20-2 | 1 |
| i0_LQ_c9867_f1p8_794_m.5152 | 7 | 40S ribosomal protein S20-2 | 1 |
| i1_LQ_c4738_f1p0_1404_m.7270 | 59 | 40S ribosomal protein S2-4 | 1 |
| 7745_0.path0_m.3577 | 22 | 40S ribosomal protein SA | 1 |
| 6733_0.path1_m.2329 | 89 | 40S ribosomal protein SA | 2 |
| 6985_0.path0_m.2739 | 542 | 4-coumarate--CoA ligase-like 5 | 2 |
| 6985_0.path0_m.2739 | 315 | 4-coumarate--CoA ligase-like 5 | 1 |
| 6989_0.path3_m.2744 | 400 | 5-methyltetrahydropteroyltriglutamate--homocysteine methyltransferase | 2 |
| i2_LQ_c8206_f1p0_2486_m.1177 | 422 | 5-methyltetrahydropteroyltriglutamate--homocysteine methyltransferase | 1 |
| 7963_0.path0_m.3904 | 155 | 60S acidic ribosomal protein P0 | 1 |
| 7963_0.path0_m.3904 | 142 | 60S acidic ribosomal protein P0 | 1 |
| 8874_0.path0_m.5151 | 47 | 60S acidic ribosomal protein P2 | 1 |
| 7537_0.path0_m.3286 | 105 | 60S ribosomal protein L10a-1 | 2 |
| 7537_0.path0_m.3286 | 129 | 60S ribosomal protein L10a-1 | 2 |
| 8440_0.path0_m.4466 | 56 | 60S ribosomal protein L11-2 | 2 |
| i0_LQ_c59177_f1p0_655_m.2236 | 82 | 60S ribosomal protein L22-2 | 3 |
| i1_LQ_c13452_f1p0_1493_m.7211 | 180 | 60S ribosomal protein L4-1 | 3 |
| i1_LQ_c13452_f1p0_1493_m.7211 | 316 | 60S ribosomal protein L4-1 | 1 |
| i1_LQ_c13452_f1p0_1493_m.7211 | 128 | 60S ribosomal protein L4-1 | 2 |
| i1_LQ_c13452_f1p0_1493_m.7211 | 312 | 60S ribosomal protein L4-1 | 3 |
| 8693_0.path2_m.4879 | 216 | 60S ribosomal protein L7-2 | 1 |
| 8693_0.path2_m.4879 | 67 | 60S ribosomal protein L7-2 | 2 |
| i0_LQ_c252949_f1p0_328_m.5448 | 8 | 65-kDa microtubule-associated protein 1 | 1 |
| i0_LQ_c393069_f1p4_948_m.4194 | 162 | Acid phosphatase 1 | 3 |
| 7538_0.path1_m.3287 | 229 | Acid phosphatase 1 | 2 |
| 7538_0.path1_m.3287 | 211 | Acid phosphatase 1 | 3 |
| i0_LQ_c393069_f1p4_948_m.4194 | 251 | Acid phosphatase 1 | 2 |
| 7538_0.path1_m.3287 | 175 | Acid phosphatase 1 | 3 |
| 8582_0.path0_m.4729 | 275 | Acidic endochitinase | 3 |
| 8582_0.path0_m.4729 | 181 | Acidic endochitinase | 3 |
| 8582_0.path0_m.4729 | 277 | Acidic endochitinase | 2 |
| 8582_0.path0_m.4729 | 110 | Acidic endochitinase | 3 |
| 8582_0.path0_m.4729 | 99 | Acidic endochitinase | 3 |
| 8582_0.path0_m.4729 | 283 | Acidic endochitinase | 2 |
| 8582_0.path0_m.4729 | 288 | Acidic endochitinase | 3 |
| 8582_0.path0_m.4729 | 113 | Acidic endochitinase | 3 |
| 1589_0.path0_m.3099 | 782 | Aconitate hydratase 2, mitochondrial | 2 |
| 1589_0.path0_m.3099 | 114 | Aconitate hydratase 2, mitochondrial | 3 |
| 1589_0.path0_m.3099 | 574 | Aconitate hydratase 2, mitochondrial | 3 |
| 1589_0.path0_m.3099 | 381 | Aconitate hydratase 2, mitochondrial | 3 |
| 1589_0.path0_m.3099 | 829 | Aconitate hydratase 2, mitochondrial | 2 |
| 1589_0.path0_m.3099 | 855 | Aconitate hydratase 2, mitochondrial | 3 |
| 1589_0.path0_m.3099 | 558 | Aconitate hydratase 2, mitochondrial | 3 |
| 2789_0.path4_m.5221 | 83 | Aconitate hydratase 2, mitochondrial | 3 |
| 1589_0.path0_m.3099 | 844 | Aconitate hydratase 2, mitochondrial | 3 |
| 6060_0.path0_m.1479 | 172 | Aconitate hydratase 2, mitochondrial | 2 |
| 1589_0.path0_m.3099 | 166 | Aconitate hydratase 2, mitochondrial | 3 |
| 2789_0.path4_m.5221 | 736 | Aconitate hydratase 2, mitochondrial | 1 |
| 1589_0.path0_m.3099 | 370 | Aconitate hydratase 2, mitochondrial | 2 |
| 1589_0.path0_m.3099 | 173 | Aconitate hydratase 2, mitochondrial | 2 |
| 1589_0.path0_m.3099 | 128 | Aconitate hydratase 2, mitochondrial | 3 |
| 1589_0.path0_m.3099 | 494 | Aconitate hydratase 2, mitochondrial | 1 |
| 6060_0.path0_m.1479 | 165 | Aconitate hydratase 2, mitochondrial | 1 |
| 1589_0.path0_m.3099 | 508 | Aconitate hydratase 2, mitochondrial | 3 |
| 1589_0.path0_m.3099 | 893 | Aconitate hydratase 2, mitochondrial | 2 |
| 2789_0.path4_m.5221 | 418 | Aconitate hydratase 2, mitochondrial | 2 |
| 2789_0.path4_m.5221 | 76 | Aconitate hydratase 2, mitochondrial | 2 |
| 1589_0.path0_m.3099 | 122 | Aconitate hydratase 2, mitochondrial | 3 |
| 1589_0.path0_m.3099 | 826 | Aconitate hydratase 2, mitochondrial | 2 |
| 2789_0.path4_m.5221 | 72 | Aconitate hydratase 2, mitochondrial | 2 |
| 6930_0.path0_m.2651 | 328 | Actin-7 | 3 |
| 6930_0.path0_m.2651 | 330 | Actin-7 | 2 |
| 6930_0.path0_m.2651 | 115 | Actin-7 | 3 |
| 6933_0.path10_m.2657 | 115 | Actin-97 | 3 |
| 6568_0.path0_m.2325 | 162 | Acyl-coenzyme A oxidase 3, peroxisomal | 2 |
| i1_LQ_c36180_f1p0_1059_m.2215 | 172 | Acylpyruvase FAHD1, mitochondrial | 3 |
| 10865_0.path0_m.1086 | 216 | Adenosine kinase 2 | 1 |
| 10865_0.path0_m.1086 | 207 | Adenosine kinase 2 | 1 |
| 5083_0.path2_m.16 | 7 | Adenosylhomocysteinase | 1 |
| 6817_0.path0_m.2451 | 72 | Adenylate kinase 4 | 3 |
| 6817_0.path0_m.2451 | 207 | Adenylate kinase 4 | 3 |
| 6817_0.path0_m.2451 | 175 | Adenylate kinase 4 | 2 |
| i0_HQ_c145293_f3p1_657_m.2527 | 96 | Adenylylsulfatase HINT1 | 3 |
| 7333_0.path7_m.3258 | 132 | ADP,ATP carrier protein | 2 |
| 7333_0.path7_m.3258 | 101 | ADP,ATP carrier protein | 3 |
| 7579_0.path0_m.3351 | 235 | ADP,ATP carrier protein 1, mitochondrial | 3 |
| 5983_0.path0_m.1328 | 331 | ADP,ATP carrier protein 1, mitochondrial | 3 |
| 7579_0.path0_m.3351 | 6 | ADP,ATP carrier protein 1, mitochondrial | 3 |
| 7579_0.path0_m.3351 | 16 | ADP,ATP carrier protein 1, mitochondrial | 3 |
| 7579_0.path0_m.3351 | 233 | ADP,ATP carrier protein 1, mitochondrial | 3 |
| 5983_0.path0_m.1328 | 342 | ADP,ATP carrier protein 1, mitochondrial | 3 |
| 7579_0.path0_m.3351 | 27 | ADP,ATP carrier protein 1, mitochondrial | 3 |
| 7579_0.path0_m.3351 | 151 | ADP,ATP carrier protein 1, mitochondrial | 3 |
| 7579_0.path0_m.3351 | 284 | ADP,ATP carrier protein 1, mitochondrial | 3 |
| 7579_0.path0_m.3351 | 47 | ADP,ATP carrier protein 1, mitochondrial | 3 |
| 5983_0.path0_m.1328 | 143 | ADP,ATP carrier protein 1, mitochondrial | 3 |
| 5983_0.path0_m.1328 | 380 | ADP,ATP carrier protein 1, mitochondrial | 1 |
| 7579_0.path0_m.3351 | 255 | ADP,ATP carrier protein 1, mitochondrial | 3 |
| 7579_0.path0_m.3351 | 246 | ADP,ATP carrier protein 1, mitochondrial | 3 |
| 7333_0.path8_m.3259 | 70 | ADP,ATP carrier protein 2, mitochondrial | 2 |
| i1_LQ_c67863_f1p0_1554_m.2255 | 175 | ADP,ATP carrier protein, mitochondrial (Fragment) | 2 |
| 8161_0.path0_m.4213 | 36 | ADP-ribosylation factor | 2 |
| 9462_0.path0_m.5804 | 65 | Agglutinin-2 | 3 |
| 7301_0.path0_m.3199 | 201 | Agglutinin-2 | 3 |
| 11235_0.path0_m.1409 | 209 | Agglutinin-2 | 3 |
| 9462_0.path0_m.5804 | 79 | Agglutinin-2 | 3 |
| 9462_0.path0_m.5804 | 241 | Agglutinin-2 | 3 |
| 11235_0.path0_m.1409 | 78 | Agglutinin-2 | 3 |
| 9462_0.path0_m.5804 | 204 | Agglutinin-2 | 1 |
| 11490_0.path1_m.1703 | 59 | Agglutinin-2 | 3 |
| 9810_0.path0_m.6285 | 193 | Agglutinin-2 | 3 |
| 9462_0.path0_m.5804 | 37 | Agglutinin-2 | 3 |
| 9462_0.path0_m.5804 | 209 | Agglutinin-2 | 3 |
| 9810_0.path0_m.6285 | 61 | Agglutinin-2 | 3 |
| i1_HQ_c9551_f2p0_1734_m.4216 | 441 | Alanine--glyoxylate aminotransferase 2 homolog 1, mitochondrial | 1 |
| 4605_0.path1_m.7665 | 443 | Aldehyde dehydrogenase family 2 member B7, mitochondrial | 2 |
| 4605_0.path1_m.7665 | 295 | Aldehyde dehydrogenase family 2 member B7, mitochondrial | 2 |
| i4_LQ_c3798_f1p0_4665_m.2516 | 528 | Aldehyde dehydrogenase family 2 member B7, mitochondrial | 1 |
| 4605_0.path1_m.7665 | 368 | Aldehyde dehydrogenase family 2 member B7, mitochondrial | 2 |
| i4_LQ_c3798_f1p0_4665_m.2516 | 515 | Aldehyde dehydrogenase family 2 member B7, mitochondrial | 1 |
| 4605_0.path1_m.7665 | 528 | Aldehyde dehydrogenase family 2 member B7, mitochondrial | 2 |
| i4_LQ_c3798_f1p0_4665_m.2516 | 295 | Aldehyde dehydrogenase family 2 member B7, mitochondrial | 1 |
| 4605_0.path1_m.7665 | 515 | Aldehyde dehydrogenase family 2 member B7, mitochondrial | 2 |
| i4_LQ_c3798_f1p0_4665_m.2516 | 443 | Aldehyde dehydrogenase family 2 member B7, mitochondrial | 1 |
| 10413_0.path0_m.608 | 21 | Alpha-1,4-glucan-protein synthase [UDP-forming] 2 | 1 |
| 10413_0.path0_m.608 | 245 | Alpha-1,4-glucan-protein synthase [UDP-forming] 2 | 2 |
| 9635_0.path0_m.6038 | 276 | Alpha-1,4-glucan-protein synthase [UDP-forming] 2 | 1 |
| 9635_0.path0_m.6038 | 81 | Alpha-1,4-glucan-protein synthase [UDP-forming] 2 | 1 |
| 10413_0.path0_m.608 | 43 | Alpha-1,4-glucan-protein synthase [UDP-forming] 2 | 1 |
| 8952_0.path0_m.5270 | 147 | Alpha-amylase_subtilisin inhibitor | 1 |
| 7359_0.path0_m.3296 | 118 | Alpha-galactosidase 1 | 2 |
| 11777_0.path0_m.2272 | 131 | Alpha-galactosidase 1 | 1 |
| i3_LQ_c29252_f1p0_3719_m.2156 | 617 | Alpha-mannosidase | 2 |
| i0_LQ_c35465_f1p1_640_m.6645 | 115 | Aminomethyltransferase, mitochondrial | 3 |
| i1_HQ_c60944_f14p0_1165_m.1032 | 126 | Annexin D2 | 1 |
| 8913_0.path2_m.5215 | 13 | Aquaporin PIP2-7 | 3 |
| 8913_0.path2_m.5215 | 141 | Aquaporin PIP2-7 | 1 |
| i1_LQ_c13021_f1p0_1244_m.6292 | 253 | Arginase 1, mitochondrial | 1 |
| i1_HQ_c14606_f2p0_1675_m.3241 | 428 | Aspartate aminotransferase, mitochondrial | 2 |
| i1_HQ_c14606_f2p0_1675_m.3241 | 75 | Aspartate aminotransferase, mitochondrial | 1 |
| i1_HQ_c14606_f2p0_1675_m.3241 | 179 | Aspartate aminotransferase, mitochondrial | 3 |
| i1_HQ_c14606_f2p0_1675_m.3241 | 374 | Aspartate aminotransferase, mitochondrial | 3 |
| 2769_0.path0_m.5164 | 114 | ATP synthase subunit alpha, chloroplastic | 3 |
| 2769_0.path0_m.5164 | 469 | ATP synthase subunit alpha, chloroplastic | 2 |
| 2769_0.path0_m.5164 | 466 | ATP synthase subunit alpha, chloroplastic | 3 |
| 4175_0.path0_m.7181 | 142 | ATP synthase subunit alpha, mitochondrial | 3 |
| 4175_0.path0_m.7181 | 463 | ATP synthase subunit alpha, mitochondrial | 1 |
| 4175_0.path0_m.7181 | 387 | ATP synthase subunit alpha, mitochondrial | 3 |
| 4175_0.path0_m.7181 | 134 | ATP synthase subunit alpha, mitochondrial | 1 |
| 4175_0.path0_m.7181 | 153 | ATP synthase subunit alpha, mitochondrial | 2 |
| 4175_0.path0_m.7181 | 432 | ATP synthase subunit alpha, mitochondrial | 2 |
| 4175_0.path0_m.7181 | 189 | ATP synthase subunit alpha, mitochondrial | 3 |
| 4175_0.path0_m.7181 | 476 | ATP synthase subunit alpha, mitochondrial | 3 |
| 4175_0.path0_m.7181 | 384 | ATP synthase subunit alpha, mitochondrial | 3 |
| 5626_0.path0_m.993 | 86 | ATP synthase subunit beta, chloroplastic | 1 |
| 5626_0.path0_m.993 | 50 | ATP synthase subunit beta, chloroplastic | 1 |
| 5626_0.path0_m.993 | 17 | ATP synthase subunit beta, chloroplastic | 2 |
| 5626_0.path0_m.993 | 392 | ATP synthase subunit beta, chloroplastic | 2 |
| 5626_0.path0_m.993 | 167 | ATP synthase subunit beta, chloroplastic | 1 |
| i1_LQ_c51375_f1p0_1803_m.7249 | 465 | ATP synthase subunit beta, mitochondrial | 1 |
| i1_LQ_c51375_f1p0_1803_m.7249 | 86 | ATP synthase subunit beta, mitochondrial | 3 |
| 4133_0.path3_m.7108 | 176 | ATP synthase subunit beta, mitochondrial | 2 |
| 4133_0.path3_m.7108 | 449 | ATP synthase subunit beta, mitochondrial | 2 |
| 4133_0.path3_m.7108 | 70 | ATP synthase subunit beta, mitochondrial | 3 |
| 3962_0.path0_m.6781 | 92 | ATP synthase subunit beta, mitochondrial | 3 |
| 4827_0.path0_m.8083 | 174 | ATP synthase subunit beta, mitochondrial | 1 |
| 4133_0.path3_m.7108 | 78 | ATP synthase subunit beta, mitochondrial | 3 |
| i1_LQ_c51375_f1p0_1803_m.7249 | 94 | ATP synthase subunit beta, mitochondrial | 3 |
| i1_LQ_c51375_f1p0_1803_m.7249 | 192 | ATP synthase subunit beta, mitochondrial | 1 |
| 3962_0.path0_m.6781 | 100 | ATP synthase subunit beta, mitochondrial | 3 |
| 7643_0.path0_m.3432 | 186 | ATP synthase subunit beta, mitochondrial | 1 |
| 4133_0.path3_m.7108 | 222 | ATP synthase subunit beta, mitochondrial | 1 |
| i0_LQ_c59477_f1p0_991_m.4245 | 100 | ATP synthase subunit d, mitochondrial | 3 |
| i0_LQ_c59477_f1p0_991_m.4245 | 17 | ATP synthase subunit d, mitochondrial | 3 |
| i0_LQ_c59477_f1p0_991_m.4245 | 102 | ATP synthase subunit d, mitochondrial | 1 |
| i0_LQ_c59477_f1p0_991_m.4245 | 7 | ATP synthase subunit d, mitochondrial | 3 |
| i0_LQ_c59477_f1p0_991_m.4245 | 149 | ATP synthase subunit d, mitochondrial | 3 |
| i0_LQ_c59477_f1p0_991_m.4245 | 80 | ATP synthase subunit d, mitochondrial | 3 |
| i0_LQ_c59477_f1p0_991_m.4245 | 87 | ATP synthase subunit d, mitochondrial | 3 |
| i0_LQ_c59477_f1p0_991_m.4245 | 14 | ATP synthase subunit d, mitochondrial | 3 |
| 10112_0.path0_m.184 | 169 | ATP synthase subunit O, mitochondrial | 3 |
| 6944_0.path0_m.2670 | 65 | ATP-citrate synthase alpha chain protein 2 | 1 |
| 6944_0.path0_m.2670 | 58 | ATP-citrate synthase alpha chain protein 2 | 3 |
| 6944_0.path0_m.2670 | 16 | ATP-citrate synthase alpha chain protein 2 | 3 |
| 5891_0.path0_m.1161 | 266 | ATP-citrate synthase beta chain protein 2 | 3 |
| 5891_0.path0_m.1161 | 590 | ATP-citrate synthase beta chain protein 2 | 1 |
| i5_LQ_c11712_f1p0_5727_m.6524 | 253 | ATP-dependent DNA helicase pfh1 | 1 |
| i1_LQ_c44477_f1p0_1911_m.5243 | 166 | ATP-dependent zinc metalloprotease FTSH 2, chloroplastic | 1 |
| 10680_0.path0_m.950 | 257 | ATP-dependent zinc metalloprotease FTSH 4, mitochondrial | 2 |
| i2_LQ_c26515_f1p0_2429_m.5439 | 132 | ATP-dependent zinc metalloprotease FTSH, chloroplastic | 2 |
| 1514_0.path5_m.3655 | 779 | Auxin response factor 2 | 1 |
| 10812_0.path0_m.1124 | 133 | Auxin-binding protein ABP19a | 3 |
| 10812_0.path0_m.1124 | 130 | Auxin-binding protein ABP19a | 3 |
| i2_LQ_c39913_f1p0_2070_m.5374 | 429 | B3 domain-containing protein Os03g0619600 | 2 |
| 10317_0.path0_m.464 | 222 | Basic secretory protease (Fragments) | 1 |
| 10317_0.path0_m.464 | 166 | Basic secretory protease (Fragments) | 1 |
| i0_LQ_c48220_f2p1_691_m.7450 | 173 | Berberine bridge enzyme-like 1 | 1 |
| 5062_0.path1_m.8498 | 138 | Berberine bridge enzyme-like 15 | 3 |
| 9778_0.path2_m.6248 | 384 | Berberine bridge enzyme-like 18 | 3 |
| 9778_0.path2_m.6248 | 376 | Berberine bridge enzyme-like 18 | 1 |
| 9778_0.path2_m.6248 | 506 | Berberine bridge enzyme-like 18 | 1 |
| 9778_0.path2_m.6248 | 469 | Berberine bridge enzyme-like 18 | 3 |
| 9778_0.path2_m.6248 | 286 | Berberine bridge enzyme-like 18 | 3 |
| 4230_0.path9_m.7330 | 74 | Berberine bridge enzyme-like 22 | 3 |
| 9500_0.path0_m.5855 | 358 | Berberine bridge enzyme-like 28 | 1 |
| 7015_0.path0_m.2795 | 263 | Berberine bridge enzyme-like 8 | 3 |
| 7015_0.path0_m.2794 | 163 | Berberine bridge enzyme-like 8 | 2 |
| 6115_0.path0_m.1574 | 110 | Beta-glucosidase 12 | 2 |
| 6115_0.path0_m.1574 | 266 | Beta-glucosidase 12 | 3 |
| 6115_0.path0_m.1574 | 113 | Beta-glucosidase 12 | 3 |
| i1_LQ_c3987_f1p0_1314_m.4264 | 60 | Bifunctional L-3-cyanoalanine synthase_cysteine synthase 1, mitochondrial | 2 |
| 4661_0.path8_m.7778 | 445 | Bifunctional purple acid phosphatase 26 | 3 |
| 4661_0.path8_m.7778 | 202 | Bifunctional purple acid phosphatase 26 | 1 |
| 9386_0.path0_m.5681 | 240 | Binding partner of ACD11 1 | 2 |
| 10208_0.path0_m.297 | 139 | BRASSINOSTEROID INSENSITIVE 1-associated receptor kinase 1 | 3 |
| i1_LQ_c5915_f1p0_1332_m.43 | 325 | BTB_POZ domain-containing protein At1g67900 | 1 |
| i0_HQ_c381237_f23p3_483_m.3371 | 46 | Calmodulin-1 | 1 |
| i2_LQ_c33428_f1p0_2326_m.4485 | 95 | Calmodulin-1_11_16 | 2 |
| 6945_0.path0_m.2671 | 56 | Calreticulin | 1 |
| 9889_0.path0_m.6272 | 55 | Calreticulin-3 | 1 |
| 6277_0.path0_m.1840 | 280 | Calumenin-B | 2 |
| i1_LQ_c27338_f1p0_1960_m.5158 | 202 | Cannabidiolic acid synthase | 3 |
| 6785_0.path1_m.2407 | 298 | Carbonic anhydrase, chloroplastic | 2 |
| 6785_0.path1_m.2407 | 188 | Carbonic anhydrase, chloroplastic | 2 |
| 6785_0.path1_m.2407 | 128 | Carbonic anhydrase, chloroplastic | 1 |
| 6785_0.path1_m.2407 | 134 | Carbonic anhydrase, chloroplastic | 1 |
| 6785_0.path1_m.2407 | 293 | Carbonic anhydrase, chloroplastic | 2 |
| 6785_0.path1_m.2407 | 249 | Carbonic anhydrase, chloroplastic | 1 |
| 6785_0.path1_m.2407 | 121 | Carbonic anhydrase, chloroplastic | 1 |
| 7335_0.path0_m.3261 | 127 | Carbonic anhydrase, chloroplastic | 1 |
| 7335_0.path0_m.3261 | 232 | Carbonic anhydrase, chloroplastic | 1 |
| 7335_0.path0_m.3261 | 237 | Carbonic anhydrase, chloroplastic | 1 |
| 5215_0.path0_m.243 | 414 | Catalase isozyme 3 | 1 |
| 8435_0.path0_m.4461 | 313 | Cathepsin B | 3 |
| 8435_0.path0_m.4461 | 98 | Cathepsin B | 2 |
| 8076_0.path0_m.4073 | 63 | CBS domain-containing protein CBSX3, mitochondrial | 3 |
| 8076_0.path0_m.4073 | 149 | CBS domain-containing protein CBSX3, mitochondrial | 2 |
| 11469_0.path0_m.1679 | 98 | CDGSH iron-sulfur domain-containing protein NEET | 2 |
| 3423_0.path0_m.6010 | 53 | Cell division control protein 6 homolog | 1 |
| 5146_0.path0_m.123 | 41 | Cell division cycle protein 48 homolog | 1 |
| 8934_0.path0_m.5246 | 127 | Chitinase 5 | 3 |
| 8934_0.path0_m.5246 | 171 | Chitinase 5 | 3 |
| 11141_0.path0_m.1297 | 252 | Chitotriosidase-1 | 3 |
| 11141_0.path0_m.1297 | 120 | Chitotriosidase-1 | 3 |
| 11882_0.path0_m.2389 | 40 | Chlorophyll a-b binding protein 21, chloroplastic | 2 |
| 8146_0.path0_m.4182 | 124 | Chlorophyll a-b binding protein 36, chloroplastic | 3 |
| 8146_0.path0_m.4182 | 42 | Chlorophyll a-b binding protein 36, chloroplastic | 3 |
| i0_HQ_c151837_f2p8_620_m.7479 | 49 | Chlorophyll a-b binding protein 6, chloroplastic | 3 |
| i0_HQ_c151837_f2p8_620_m.7479 | 62 | Chlorophyll a-b binding protein 6, chloroplastic | 3 |
| 7467_0.path0_m.3184 | 238 | Chlorophyll a-b binding protein 8, chloroplastic | 3 |
| 7383_0.path0_m.3334 | 94 | Chlorophyll a-b binding protein CP24 10A, chloroplastic | 1 |
| 7383_0.path0_m.3334 | 53 | Chlorophyll a-b binding protein CP24 10A, chloroplastic | 3 |
| 8352_0.path0_m.4346 | 113 | Chlorophyll a-b binding protein CP26, chloroplastic | 3 |
| 8352_0.path0_m.4346 | 65 | Chlorophyll a-b binding protein CP26, chloroplastic | 2 |
| 6991_0.path1_m.2759 | 238 | Chlorophyll a-b binding protein CP29.1, chloroplastic | 1 |
| 7598_0.path21_m.3382 | 41 | Chlorophyll a-b binding protein of LHCII type I, chloroplastic (Fragment) | 3 |
| 10025_0.path0_m.37 | 7 | Chlorophyll a-b binding protein of LHCII type I, chloroplastic (Fragment) | 3 |
| i1_LQ_c50855_f1p1_1111_m.2233 | 50 | Chlorophyllase-1 | 3 |
| 7997_0.path0_m.3961 | 74 | Chloroplast stem-loop binding protein of 41 kDa b, chloroplastic | 3 |
| 7997_0.path0_m.3961 | 152 | Chloroplast stem-loop binding protein of 41 kDa b, chloroplastic | 3 |
| 7997_0.path0_m.3961 | 143 | Chloroplast stem-loop binding protein of 41 kDa b, chloroplastic | 1 |
| i1_LQ_c60104_f1p0_1059_m.3161 | 198 | Chorismate synthase 1, chloroplastic | 1 |
| 9380_0.path0_m.5675 | 156 | Chorismate synthase 1, chloroplastic | 2 |
| i1_LQ_c60104_f1p0_1059_m.3161 | 35 | Chorismate synthase 1, chloroplastic | 1 |
| i1_LQ_c31882_f1p0_1297_m.6613 | 282 | Cinnamoyl-CoA reductase 1 | 2 |
| i1_LQ_c31882_f1p0_1297_m.6613 | 168 | Cinnamoyl-CoA reductase 1 | 1 |
| i1_LQ_c31882_f1p0_1297_m.6613 | 305 | Cinnamoyl-CoA reductase 1 | 3 |
| 9209_0.path0_m.5429 | 228 | Citrate synthase, glyoxysomal | 3 |
| 9257_0.path0_m.5499 | 327 | Citrate synthase, mitochondrial | 1 |
| 9257_0.path1_m.5500 | 372 | Citrate synthase, mitochondrial | 1 |
| 9257_0.path0_m.5499 | 130 | Citrate synthase, mitochondrial | 1 |
| 9257_0.path0_m.5499 | 270 | Citrate synthase, mitochondrial | 2 |
| 9257_0.path1_m.5500 | 401 | Citrate synthase, mitochondrial | 3 |
| 9257_0.path0_m.5499 | 281 | Citrate synthase, mitochondrial | 2 |
| 9257_0.path0_m.5499 | 48 | Citrate synthase, mitochondrial | 1 |
| 9257_0.path0_m.5499 | 186 | Citrate synthase, mitochondrial | 1 |
| 9257_0.path1_m.5500 | 346 | Citrate synthase, mitochondrial | 3 |
| 9257_0.path0_m.5499 | 265 | Citrate synthase, mitochondrial | 3 |
| 9257_0.path0_m.5499 | 288 | Citrate synthase, mitochondrial | 1 |
| 9257_0.path0_m.5499 | 234 | Citrate synthase, mitochondrial | 1 |
| 9257_0.path0_m.5499 | 308 | Citrate synthase, mitochondrial | 1 |
| 207_0.path8_m.4254 | 951 | Clathrin heavy chain 1 | 1 |
| 207_0.path17_m.4258 | 1530 | Clathrin heavy chain 1 | 1 |
| 207_0.path17_m.4258 | 102 | Clathrin heavy chain 1 | 1 |
| i1_HQ_c2639_f2p0_1518_m.4247 | 209 | Cysteine desulfurase 1, chloroplastic | 1 |
| 4922_0.path3_m.8283 | 257 | Cysteine proteinase RD21A | 3 |
| 4922_0.path3_m.8283 | 261 | Cysteine proteinase RD21A | 3 |
| 4922_0.path3_m.8283 | 338 | Cysteine proteinase RD21A | 2 |
| i1_HQ_c60950_f14p0_1160_m.1131 | 220 | Cysteine synthase | 3 |
| i1_HQ_c60950_f14p0_1160_m.1131 | 194 | Cysteine synthase | 1 |
| 11755_0.path14_m.2228 | 291 | Cysteine synthase, chloroplastic_chromoplastic | 1 |
| 11755_0.path10_m.2226 | 93 | Cysteine synthase, chloroplastic_chromoplastic | 1 |
| 7809_0.path0_m.3670 | 147 | Cytochrome b6-f complex iron-sulfur subunit, chloroplastic | 3 |
| 7809_0.path0_m.3670 | 133 | Cytochrome b6-f complex iron-sulfur subunit, chloroplastic | 3 |
| i1_LQ_c70037_f1p0_1177_m.2262 | 94 | Cytochrome c oxidase subunit 5b-1, mitochondrial | 3 |
| 6328_0.path0_m.1926 | 291 | Cytochrome c1 2, heme protein, mitochondrial | 3 |
| 6706_0.path0_m.2288 | 174 | Cytochrome f | 3 |
| 6706_0.path0_m.2288 | 216 | Cytochrome f | 3 |
| 6706_0.path0_m.2288 | 180 | Cytochrome f | 3 |
| 6706_0.path0_m.2288 | 200 | Cytochrome f | 1 |
| 8929_0.path0_m.5242 | 99 | Cytochrome P450 71D95 | 1 |
| 5191_0.path0_m.197 | 78 | Cytosolic endo-beta-N-acetylglucosaminidase 1 | 1 |
| 9489_0.path0_m.5840 | 54 | DEAD-box ATP-dependent RNA helicase 56 | 1 |
| 8598_0.path6_m.4746 | 235 | Dehydrin ERD10 | 1 |
| i0_LQ_c12020_f2p1_945_m.400 | 101 | Desiccation-related protein PCC13-62 | 3 |
| i0_LQ_c12020_f2p1_945_m.400 | 273 | Desiccation-related protein PCC13-62 | 2 |
| 5592_0.path0_m.933 | 173 | Dihydrolipoyl dehydrogenase 1, mitochondrial | 3 |
| 5592_0.path0_m.933 | 354 | Dihydrolipoyl dehydrogenase 1, mitochondrial | 3 |
| 5592_0.path0_m.933 | 166 | Dihydrolipoyl dehydrogenase 1, mitochondrial | 3 |
| 5592_0.path0_m.933 | 181 | Dihydrolipoyl dehydrogenase 1, mitochondrial | 3 |
| 5592_0.path0_m.933 | 448 | Dihydrolipoyl dehydrogenase 1, mitochondrial | 3 |
| 5592_0.path0_m.933 | 240 | Dihydrolipoyl dehydrogenase 1, mitochondrial | 1 |
| 5592_0.path0_m.933 | 306 | Dihydrolipoyl dehydrogenase 1, mitochondrial | 3 |
| 5592_0.path0_m.933 | 185 | Dihydrolipoyl dehydrogenase 1, mitochondrial | 2 |
| 5592_0.path0_m.933 | 176 | Dihydrolipoyl dehydrogenase 1, mitochondrial | 3 |
| 5592_0.path0_m.933 | 205 | Dihydrolipoyl dehydrogenase 1, mitochondrial | 3 |
| 5592_0.path0_m.933 | 97 | Dihydrolipoyl dehydrogenase 1, mitochondrial | 3 |
| 5592_0.path0_m.933 | 133 | Dihydrolipoyl dehydrogenase 1, mitochondrial | 3 |
| 5592_0.path0_m.933 | 218 | Dihydrolipoyl dehydrogenase 1, mitochondrial | 3 |
| 5592_0.path0_m.933 | 207 | Dihydrolipoyl dehydrogenase 1, mitochondrial | 3 |
| i1_LQ_c26180_f2p0_1342_m.6236 | 203 | Dihydrolipoyllysine-residue acetyltransferase component 1 of pyruvate dehydrogenase complex, mitochondrial | 3 |
| 9666_0.path0_m.6084 | 157 | Dihydrolipoyllysine-residue acetyltransferase component 3 of pyruvate dehydrogenase complex, mitochondrial | 3 |
| 9666_0.path0_m.6084 | 150 | Dihydrolipoyllysine-residue acetyltransferase component 3 of pyruvate dehydrogenase complex, mitochondrial | 3 |
| 9666_0.path0_m.6084 | 204 | Dihydrolipoyllysine-residue acetyltransferase component 3 of pyruvate dehydrogenase complex, mitochondrial | 1 |
| 4752_1.path0_m.7937 | 249 | Dihydrolipoyllysine-residue succinyltransferase component of 2-oxoglutarate dehydrogenase complex 2, mitochondrial | 3 |
| 4752_1.path0_m.7937 | 315 | Dihydrolipoyllysine-residue succinyltransferase component of 2-oxoglutarate dehydrogenase complex 2, mitochondrial | 1 |
| 4752_1.path0_m.7937 | 266 | Dihydrolipoyllysine-residue succinyltransferase component of 2-oxoglutarate dehydrogenase complex 2, mitochondrial | 1 |
| 4752_1.path0_m.7937 | 322 | Dihydrolipoyllysine-residue succinyltransferase component of 2-oxoglutarate dehydrogenase complex 2, mitochondrial | 3 |
| 4752_1.path0_m.7937 | 124 | Dihydrolipoyllysine-residue succinyltransferase component of 2-oxoglutarate dehydrogenase complex 2, mitochondrial | 3 |
| i4_LQ_c16173_f1p0_4068_m.2166 | 346 | DNA repair protein RAD50 | 1 |
| 913_0.path0_m.5315 | 106 | DNA-directed RNA polymerase IV subunit 1 | 1 |
| 5794_0.path1_m.1289 | 274 | Dolichyl-diphosphooligosaccharide--protein glycosyltransferase subunit 2 | 1 |
| i2_LQ_c34331_f1p0_2422_m.7289 | 456 | E3 ubiquitin-protein ligase KEG | 1 |
| 226_0.path11_m.4605 | 276 | E3 ubiquitin-protein ligase UPL2 | 1 |
| i1_LQ_c27071_f1p0_1590_m.5417 | 396 | Elongation factor 1-alpha | 3 |
| i1_LQ_c27071_f1p0_1590_m.5417 | 172 | Elongation factor 1-alpha | 1 |
| i1_LQ_c27071_f1p0_1590_m.5417 | 427 | Elongation factor 1-alpha | 3 |
| i1_LQ_c27071_f1p0_1590_m.5417 | 243 | Elongation factor 1-alpha | 3 |
| 7466_0.path0_m.3183 | 207 | Elongation factor 1-delta 2 | 3 |
| 7466_0.path0_m.3183 | 37 | Elongation factor 1-delta 2 | 1 |
| 5988_0.path84_m.1341 | 722 | Elongation factor 2 | 1 |
| i2_LQ_c85911_f1p0_2750_m.4357 | 722 | Elongation factor 2 | 1 |
| i0_LQ_c57277_f1p0_890_m.2207 | 207 | Elongation factor P | 2 |
| i1_LQ_c48019_f1p0_1783_m.5563 | 310 | Elongation factor Tu, chloroplastic | 3 |
| 9425_0.path0_m.5723 | 291 | Elongation factor Tu, mitochondrial | 1 |
| 9425_0.path0_m.5723 | 305 | Elongation factor Tu, mitochondrial | 2 |
| 9425_0.path0_m.5723 | 445 | Elongation factor Tu, mitochondrial | 3 |
| 11558_0.path0_m.1786 | 129 | Endo-1,3;1,4-beta-D-glucanase | 1 |
| 6171_0.path0_m.1661 | 356 | Epidermis-specific secreted glycoprotein EP1 | 1 |
| 8229_0.path0_m.4151 | 35 | Eukaryotic initiation factor 4A-10 | 2 |
| 9229_0.path0_m.5460 | 66 | Farnesyl pyrophosphate synthase 1 | 1 |
| 9229_0.path0_m.5460 | 288 | Farnesyl pyrophosphate synthase 1 | 2 |
| 6576_0.path1_m.2343 | 66 | Farnesyl pyrophosphate synthase 1 | 1 |
| 6551_0.path0_m.2298 | 146 | Fatty acid hydroperoxide lyase, chloroplastic | 1 |
| 6551_0.path0_m.2298 | 366 | Fatty acid hydroperoxide lyase, chloroplastic | 2 |
| 6551_0.path0_m.2298 | 59 | Fatty acid hydroperoxide lyase, chloroplastic | 3 |
| 281_0.path2_m.5265 | 930 | Ferredoxin-dependent glutamate synthase, chloroplastic | 2 |
| i1_LQ_c76228_f1p0_1462_m.6339 | 120 | Ferredoxin--NADP reductase, leaf-type isozyme, chloroplastic | 2 |
| 10004_0.path0_m.7 | 120 | Ferritin-3, chloroplastic | 1 |
| 10683_0.path0_m.953 | 24 | Formate dehydrogenase, mitochondrial | 2 |
| 10683_0.path0_m.953 | 261 | Formate dehydrogenase, mitochondrial | 3 |
| 10683_0.path0_m.953 | 123 | Formate dehydrogenase, mitochondrial | 2 |
| 6405_0.path5_m.2044 | 52 | FRIGIDA-like protein 3 | 1 |
| 6405_0.path5_m.2044 | 55 | FRIGIDA-like protein 3 | 1 |
| 10152_0.path0_m.229 | 369 | Fructose-bisphosphate aldolase 2, chloroplastic | 3 |
| 10152_0.path0_m.229 | 61 | Fructose-bisphosphate aldolase 2, chloroplastic | 3 |
| 10152_0.path0_m.229 | 296 | Fructose-bisphosphate aldolase 2, chloroplastic | 1 |
| 11884_0.path0_m.2391 | 40 | Fructose-bisphosphate aldolase 6, cytosolic | 3 |
| 11884_0.path0_m.2391 | 357 | Fructose-bisphosphate aldolase 6, cytosolic | 3 |
| 5677_0.path0_m.1074 | 461 | Galactokinase | 1 |
| 5214_0.path7_m.240 | 126 | Gamma aminobutyrate transaminase 3, chloroplastic | 1 |
| 5214_0.path7_m.241 | 130 | Gamma aminobutyrate transaminase 3, chloroplastic | 1 |
| 6531_0.path0_m.2266 | 187 | Gamma carbonic anhydrase 1, mitochondrial | 2 |
| 7440_0.path0_m.3146 | 110 | Gamma carbonic anhydrase 1, mitochondrial | 3 |
| 7440_0.path0_m.3146 | 187 | Gamma carbonic anhydrase 1, mitochondrial | 2 |
| 10224_0.path0_m.313 | 69 | Gamma-interferon-inducible lysosomal thiol reductase | 2 |
| 6858_0.path0_m.2515 | 196 | GDSL esterase_lipase At1g29670 | 1 |
| 11972_0.path1_m.2816 | 1402 | Genome polyprotein | 1 |
| 3111_0.path0_m.5349 | 1382 | Genome polyprotein | 1 |
| 7734_0.path0_m.3563 | 288 | Glucan endo-1,3-beta-glucosidase, acidic isoform PR-Q' | 3 |
| 7734_0.path0_m.3563 | 196 | Glucan endo-1,3-beta-glucosidase, acidic isoform PR-Q' | 3 |
| i1_LQ_c32795_f1p0_1251_m.5504 | 332 | Glucan endo-1,3-beta-glucosidase, acidic isoform PR-Q' | 3 |
| 7734_0.path0_m.3563 | 330 | Glucan endo-1,3-beta-glucosidase, acidic isoform PR-Q' | 3 |
| 4464_0.path0_m.7377 | 179 | Glucose-6-phosphate 1-dehydrogenase, cytoplasmic isoform | 1 |
| i0_LQ_c59053_f1p2_783_m.2235 | 150 | Glucosidase 2 subunit beta | 1 |
| i0_LQ_c242702_f1p1_601_m.4135 | 25 | Glutamate dehydrogenase 2 | 2 |
| i0_LQ_c242702_f1p1_601_m.4135 | 5 | Glutamate dehydrogenase 2 | 1 |
| i2_LQ_c73255_f1p0_2257_m.2560 | 28 | Glutamate dehydrogenase B | 1 |
| 5304_0.path0_m.401 | 362 | Glutamate-1-semialdehyde 2,1-aminomutase, chloroplastic | 1 |
| i1_HQ_c41079_f2p0_1865_m.5127 | 54 | Glutamate--glyoxylate aminotransferase 2 | 3 |
| i1_HQ_c41079_f2p0_1865_m.5127 | 90 | Glutamate--glyoxylate aminotransferase 2 | 1 |
| i1_HQ_c41079_f2p0_1865_m.5127 | 437 | Glutamate--glyoxylate aminotransferase 2 | 1 |
| 6440_0.path0_m.2112 | 322 | Glutamine synthetase cytosolic isozyme | 3 |
| i1_LQ_c11546_f1p0_1553_m.7464 | 182 | Glutamine synthetase cytosolic isozyme | 2 |
| 8610_0.path0_m.4759 | 305 | Glutamine synthetase cytosolic isozyme | 1 |
| 8610_0.path0_m.4759 | 272 | Glutamine synthetase cytosolic isozyme | 1 |
| 6440_0.path0_m.2112 | 312 | Glutamine synthetase cytosolic isozyme | 3 |
| 11907_0.path0_m.2413 | 288 | Glutamine synthetase cytosolic isozyme | 2 |
| 6440_0.path0_m.2112 | 289 | Glutamine synthetase cytosolic isozyme | 3 |
| 6440_0.path0_m.2112 | 66 | Glutamine synthetase cytosolic isozyme | 2 |
| 11907_0.path0_m.2413 | 311 | Glutamine synthetase cytosolic isozyme | 3 |
| 9538_0.path0_m.5901 | 168 | Glutamine synthetase, chloroplastic | 3 |
| 9538_0.path0_m.5901 | 101 | Glutamine synthetase, chloroplastic | 3 |
| 9538_0.path0_m.5901 | 354 | Glutamine synthetase, chloroplastic | 3 |
| 4520_0.path2_m.7505 | 189 | Glutathione reductase, chloroplastic (Fragment) | 1 |
| 4520_0.path2_m.7505 | 353 | Glutathione reductase, chloroplastic (Fragment) | 1 |
| 4520_0.path2_m.7505 | 185 | Glutathione reductase, chloroplastic (Fragment) | 3 |
| 4225_0.path0_m.7327 | 137 | Glutathione reductase, cytosolic | 1 |
| 7028_0.path1_m.2813 | 103 | Glutathione S-transferase DHAR3, chloroplastic | 1 |
| 7028_0.path1_m.2813 | 265 | Glutathione S-transferase DHAR3, chloroplastic | 3 |
| i1_LQ_c6725_f1p0_1783_m.1515 | 55 | Glutathione S-transferase F10 | 2 |
| i2_LQ_c96775_f1p0_2002_m.4473 | 55 | Glutathione S-transferase F11 | 3 |
| 7428_0.path0_m.3131 | 208 | Glutathione S-transferase T1 | 3 |
| 11747_0.path1_m.2202 | 4 | Glutathione S-transferase T1 | 2 |
| 8224_0.path0_m.4143 | 213 | Glutathione S-transferase U25 | 1 |
| i1_LQ_c14475_f1p0_1196_m.6557 | 183 | Glyceraldehyde-3-phosphate dehydrogenase A, chloroplastic (Fragment) | 2 |
| 6986_0.path0_m.2740 | 87 | Glyceraldehyde-3-phosphate dehydrogenase A, chloroplastic (Fragment) | 1 |
| i1_LQ_c14475_f1p0_1196_m.6557 | 233 | Glyceraldehyde-3-phosphate dehydrogenase A, chloroplastic (Fragment) | 2 |
| i1_LQ_c14475_f1p0_1196_m.6557 | 317 | Glyceraldehyde-3-phosphate dehydrogenase A, chloroplastic (Fragment) | 1 |
| i1_LQ_c14475_f1p0_1196_m.6557 | 105 | Glyceraldehyde-3-phosphate dehydrogenase A, chloroplastic (Fragment) | 2 |
| 6986_0.path0_m.2740 | 181 | Glyceraldehyde-3-phosphate dehydrogenase A, chloroplastic (Fragment) | 1 |
| i1_LQ_c14475_f1p0_1196_m.6557 | 280 | Glyceraldehyde-3-phosphate dehydrogenase A, chloroplastic (Fragment) | 1 |
| i1_LQ_c14475_f1p0_1196_m.6557 | 290 | Glyceraldehyde-3-phosphate dehydrogenase A, chloroplastic (Fragment) | 2 |
| 6986_0.path0_m.2740 | 288 | Glyceraldehyde-3-phosphate dehydrogenase A, chloroplastic (Fragment) | 1 |
| 6986_0.path0_m.2740 | 135 | Glyceraldehyde-3-phosphate dehydrogenase A, chloroplastic (Fragment) | 1 |
| 6986_0.path0_m.2740 | 231 | Glyceraldehyde-3-phosphate dehydrogenase A, chloroplastic (Fragment) | 1 |
| 6986_0.path0_m.2740 | 103 | Glyceraldehyde-3-phosphate dehydrogenase A, chloroplastic (Fragment) | 1 |
| 6986_0.path0_m.2740 | 315 | Glyceraldehyde-3-phosphate dehydrogenase A, chloroplastic (Fragment) | 1 |
| 8394_0.path0_m.4400 | 271 | Glyceraldehyde-3-phosphate dehydrogenase B, chloroplastic | 3 |
| 8394_0.path0_m.4400 | 119 | Glyceraldehyde-3-phosphate dehydrogenase B, chloroplastic | 3 |
| 8394_0.path0_m.4400 | 104 | Glyceraldehyde-3-phosphate dehydrogenase B, chloroplastic | 2 |
| 8394_0.path0_m.4400 | 91 | Glyceraldehyde-3-phosphate dehydrogenase B, chloroplastic | 3 |
| 9946_0.path0_m.6365 | 238 | Glyceraldehyde-3-phosphate dehydrogenase, cytosolic | 2 |
| 6443_0.path0_m.2119 | 245 | Glyceraldehyde-3-phosphate dehydrogenase, cytosolic | 1 |
| 6443_0.path0_m.2119 | 249 | Glyceraldehyde-3-phosphate dehydrogenase, cytosolic | 1 |
| 9946_0.path0_m.6365 | 242 | Glyceraldehyde-3-phosphate dehydrogenase, cytosolic | 2 |
| 9946_0.path0_m.6365 | 352 | Glyceraldehyde-3-phosphate dehydrogenase, cytosolic | 2 |
| 9946_0.path0_m.6365 | 78 | Glyceraldehyde-3-phosphate dehydrogenase, cytosolic | 1 |
| 2765_0.path4_m.5155 | 419 | Glycerophosphodiester phosphodiesterase GDPDL3 | 1 |
| 11093_0.path0_m.1258 | 154 | Glycine cleavage system H protein 3, mitochondrial | 3 |
| 11093_0.path0_m.1258 | 140 | Glycine cleavage system H protein 3, mitochondrial | 3 |
| 11093_0.path0_m.1258 | 81 | Glycine cleavage system H protein 3, mitochondrial | 2 |
| 11093_0.path0_m.1258 | 47 | Glycine cleavage system H protein 3, mitochondrial | 3 |
| 3539_0.path0_m.6388 | 867 | Glycine dehydrogenase (decarboxylating), mitochondrial | 3 |
| 3539_0.path0_m.6388 | 863 | Glycine dehydrogenase (decarboxylating), mitochondrial | 1 |
| 3539_0.path0_m.6388 | 998 | Glycine dehydrogenase (decarboxylating), mitochondrial | 2 |
| 3539_0.path0_m.6388 | 376 | Glycine dehydrogenase (decarboxylating), mitochondrial | 1 |
| 3539_0.path0_m.6388 | 700 | Glycine dehydrogenase (decarboxylating), mitochondrial | 3 |
| 3539_0.path0_m.6388 | 359 | Glycine dehydrogenase (decarboxylating), mitochondrial | 2 |
| 3539_0.path0_m.6388 | 1001 | Glycine dehydrogenase (decarboxylating), mitochondrial | 1 |
| 3539_0.path0_m.6388 | 441 | Glycine dehydrogenase (decarboxylating), mitochondrial | 1 |
| 7564_0.path1_m.3329 | 555 | Glycine--tRNA ligase, mitochondrial 1 | 1 |
| 7564_0.path1_m.3329 | 395 | Glycine--tRNA ligase, mitochondrial 1 | 1 |
| 5250_0.path0_m.317 | 709 | Glyoxysomal fatty acid beta-oxidation multifunctional protein MFP-a | 3 |
| 5250_0.path0_m.317 | 355 | Glyoxysomal fatty acid beta-oxidation multifunctional protein MFP-a | 3 |
| 5250_0.path0_m.317 | 374 | Glyoxysomal fatty acid beta-oxidation multifunctional protein MFP-a | 3 |
| 7053_0.path0_m.2858 | 169 | GTP-binding nuclear protein Ran_TC4 | 2 |
| 7053_0.path0_m.2858 | 58 | GTP-binding nuclear protein Ran_TC4 | 1 |
| i1_LQ_c36298_f1p3_1050_m.248 | 63 | GTP-binding protein SAR1A | 2 |
| 5048_0.path0_m.8474 | 206 | Guanosine nucleotide diphosphate dissociation inhibitor 1 | 3 |
| 4053_0.path0_m.6952 | 34 | Heat shock 70 kDa protein, mitochondrial | 1 |
| 6237_0.path0_m.1765 | 107 | Heat shock 70 kDa protein, mitochondrial | 2 |
| 4053_0.path0_m.6952 | 277 | Heat shock 70 kDa protein, mitochondrial | 1 |
| 4053_0.path0_m.6952 | 294 | Heat shock 70 kDa protein, mitochondrial | 1 |
| 6237_0.path0_m.1765 | 350 | Heat shock 70 kDa protein, mitochondrial | 2 |
| 5730_0.path0_m.1191 | 414 | Heat shock cognate 70 kDa protein 2 | 3 |
| 5730_0.path0_m.1191 | 89 | Heat shock cognate 70 kDa protein 2 | 1 |
| 5730_0.path0_m.1191 | 237 | Heat shock cognate 70 kDa protein 2 | 3 |
| 5730_0.path0_m.1191 | 176 | Heat shock cognate 70 kDa protein 2 | 3 |
| 3483_0.path0_m.6126 | 415 | Heat shock cognate protein 80 | 1 |
| 4173_0.path0_m.7176 | 449 | Heat shock cognate protein 80 | 2 |
| 4173_0.path0_m.7176 | 91 | Heat shock cognate protein 80 | 2 |
| 4173_0.path0_m.7176 | 351 | Heat shock cognate protein 80 | 1 |
| 4173_0.path0_m.7176 | 227 | Heat shock cognate protein 80 | 1 |
| 3483_0.path0_m.6126 | 587 | Heat shock cognate protein 80 | 1 |
| 4173_0.path0_m.7176 | 546 | Heat shock cognate protein 80 | 1 |
| 4173_0.path0_m.7176 | 582 | Heat shock cognate protein 80 | 3 |
| 3483_0.path0_m.6126 | 253 | Heat shock cognate protein 80 | 1 |
| 3483_0.path0_m.6126 | 56 | Heat shock cognate protein 80 | 1 |
| 4173_0.path0_m.7176 | 287 | Heat shock cognate protein 80 | 1 |
| 9376_0.path0_m.5665 | 253 | Heat shock protein 81-1 | 1 |
| 6694_0.path0_m.2272 | 127 | Heat shock protein 90-6, mitochondrial | 1 |
| 71_0.path1_m.2935 | 1681 | Helicase protein MOM1 | 2 |
| i2_LQ_c21171_f1p1_2202_m.2177 | 92 | Hevamine-A | 3 |
| 6709_0.path0_m.2293 | 558 | Histidine--tRNA ligase, cytoplasmic | 1 |
| 10847_0.path0_m.1046 | 50 | Histone H2A | 3 |
| i0_LQ_c32917_f1p0_664_m.2650 | 107 | Histone H2B | 3 |
| i0_LQ_c209190_f3p0_503_m.6215 | 106 | Histone H2B.9 | 3 |
| i0_LQ_c209190_f3p0_503_m.6215 | 64 | Histone H2B.9 | 3 |
| i0_LQ_c209190_f3p0_503_m.6215 | 67 | Histone H2B.9 | 3 |
| i0_LQ_c209190_f3p0_503_m.6215 | 59 | Histone H2B.9 | 1 |
| i0_LQ_c209190_f3p0_503_m.6215 | 107 | Histone H2B.9 | 3 |
| i0_LQ_c209190_f3p0_503_m.6215 | 129 | Histone H2B.9 | 3 |
| i0_HQ_c170292_f2p7_674_m.7230 | 123 | Histone H3.3 | 3 |
| i0_HQ_c170292_f2p7_674_m.7230 | 57 | Histone H3.3 | 3 |
| 9745_0.path0_m.6197 | 92 | Histone H4 | 3 |
| 9745_0.path0_m.6197 | 32 | Histone H4 | 3 |
| 9212_0.path0_m.5436 | 423 | Inactive beta-amylase 9 | 1 |
| 9032_0.path0_m.5190 | 72 | Inactive protein RESTRICTED TEV MOVEMENT 1 | 3 |
| 9032_0.path0_m.5190 | 120 | Inactive protein RESTRICTED TEV MOVEMENT 1 | 1 |
| 9032_0.path0_m.5190 | 115 | Inactive protein RESTRICTED TEV MOVEMENT 1 | 1 |
| i1_LQ_c36162_f1p0_1024_m.6330 | 145 | Internal alternative NAD(P)H-ubiquinone oxidoreductase A2, mitochondrial | 3 |
| 8601_0.path0_m.4749 | 104 | Isocitrate dehydrogenase [NAD] catalytic subunit 5, mitochondrial | 1 |
| 8601_0.path0_m.4749 | 205 | Isocitrate dehydrogenase [NAD] catalytic subunit 5, mitochondrial | 3 |
| 8601_0.path0_m.4749 | 168 | Isocitrate dehydrogenase [NAD] catalytic subunit 5, mitochondrial | 3 |
| 8601_0.path0_m.4749 | 332 | Isocitrate dehydrogenase [NAD] catalytic subunit 5, mitochondrial | 3 |
| 8024_0.path0_m.4009 | 77 | Isocitrate dehydrogenase [NADP] | 1 |
| i2_LQ_c68897_f1p0_2701_m.4177 | 65 | Isovaleryl-CoA dehydrogenase, mitochondrial | 2 |
| 8722_0.path0_m.4911 | 113 | Jacalin-related lectin 19 | 3 |
| 8722_0.path0_m.4911 | 59 | Jacalin-related lectin 19 | 2 |
| 8722_0.path0_m.4911 | 64 | Jacalin-related lectin 19 | 1 |
| 8722_0.path0_m.4911 | 165 | Jacalin-related lectin 19 | 1 |
| i1_LQ_c14416_f1p0_1939_m.82 | 117 | Ketol-acid reductoisomerase, chloroplastic | 2 |
| 8415_0.path2_m.4435 | 194 | Kunitz trypsin inhibitor 2 | 3 |
| 8415_0.path2_m.4435 | 101 | Kunitz trypsin inhibitor 2 | 3 |
| 8415_0.path2_m.4435 | 102 | Kunitz trypsin inhibitor 2 | 3 |
| 8415_0.path2_m.4435 | 162 | Kunitz trypsin inhibitor 2 | 3 |
| 8415_0.path2_m.4435 | 159 | Kunitz trypsin inhibitor 2 | 1 |
| 8554_0.path0_m.4690 | 106 | Kunitz-type trypsin inhibitor-like 2 protein | 3 |
| 8274_0.path0_m.4219 | 126 | Lactoylglutathione lyase | 2 |
| 10159_0.path0_m.239 | 3 | L-ascorbate peroxidase 1, cytosolic | 2 |
| 7402_0.path0_m.3092 | 30 | L-ascorbate peroxidase 1, cytosolic | 1 |
| 7402_0.path0_m.3092 | 3 | L-ascorbate peroxidase 1, cytosolic | 1 |
| 10159_0.path0_m.239 | 30 | L-ascorbate peroxidase 1, cytosolic | 2 |
| 8750_0.path0_m.4965 | 267 | L-ascorbate peroxidase 3, peroxisomal | 3 |
| 5772_0.path0_m.1247 | 144 | L-ascorbate peroxidase T, chloroplastic | 3 |
| 5772_0.path0_m.1247 | 103 | L-ascorbate peroxidase T, chloroplastic | 3 |
| 5772_0.path0_m.1247 | 156 | L-ascorbate peroxidase T, chloroplastic | 1 |
| 5772_0.path0_m.1247 | 345 | L-ascorbate peroxidase T, chloroplastic | 3 |
| 5772_0.path0_m.1247 | 365 | L-ascorbate peroxidase T, chloroplastic | 3 |
| 8533_0.path0_m.4638 | 140 | Latex serine proteinase inhibitor | 2 |
| 8835_0.path0_m.5093 | 123 | Latex serine proteinase inhibitor | 3 |
| 8835_0.path0_m.5093 | 105 | Latex serine proteinase inhibitor | 3 |
| 3549_0.path0_m.6407 | 251 | Leucine aminopeptidase 2, chloroplastic | 3 |
| 5541_0.path17_m.848 | 218 | Linoleate 13S-lipoxygenase 2-1, chloroplastic | 2 |
| 5541_0.path17_m.848 | 269 | Linoleate 13S-lipoxygenase 2-1, chloroplastic | 1 |
| 5541_0.path5_m.843 | 218 | Linoleate 13S-lipoxygenase 2-1, chloroplastic | 1 |
| 5541_0.path5_m.843 | 269 | Linoleate 13S-lipoxygenase 2-1, chloroplastic | 1 |
| 1844_0.path0_m.3705 | 306 | Lipoxygenase 6, chloroplastic | 2 |
| 4989_0.path4_m.8397 | 377 | Luminal-binding protein 5 | 2 |
| 4989_0.path4_m.8397 | 490 | Luminal-binding protein 5 | 1 |
| 4989_0.path4_m.8397 | 455 | Luminal-binding protein 5 | 2 |
| 3626_0.path0_m.6573 | 147 | Luminal-binding protein 5 | 1 |
| 7228_0.path0_m.3100 | 383 | Luminal-binding protein 5 | 1 |
| 3626_0.path0_m.6573 | 219 | Luminal-binding protein 5 | 1 |
| 7228_0.path0_m.3100 | 377 | Luminal-binding protein 5 | 1 |
| 7228_0.path0_m.3100 | 455 | Luminal-binding protein 5 | 1 |
| 4989_0.path4_m.8397 | 383 | Luminal-binding protein 5 | 2 |
| 7620_0.path0_m.3409 | 176 | Macro domain-containing protein XCC3184 | 3 |
| 9427_0.path0_m.5725 | 99 | Macrophage migration inhibitory factor homolog | 3 |
| i0_LQ_c367943_f1p1_653_m.6289 | 29 | Major allergen Pru ar 1 | 1 |
| i0_LQ_c367943_f1p1_653_m.6289 | 116 | Major allergen Pru ar 1 | 3 |
| 6295_0.path5_m.1860 | 111 | Malate dehydrogenase | 1 |
| i3_LQ_c3189_f1p0_3808_m.6480 | 791 | Mediator of DNA damage checkpoint protein 1 | 3 |
| 10395_0.path0_m.580 | 161 | Membrane steroid-binding protein 2 | 2 |
| 10395_0.path0_m.580 | 164 | Membrane steroid-binding protein 2 | 2 |
| 7757_0.path2_m.3599 | 187 | Membrane steroid-binding protein 2 | 1 |
| i1_LQ_c23898_f1p0_1584_m.1532 | 394 | Methylthioribose kinase | 1 |
| 8426_0.path0_m.4449 | 32 | Miraculin | 1 |
| i0_LQ_c178185_f1p3_749_m.6753 | 94 | Miraculin | 2 |
| i0_LQ_c178185_f1p3_749_m.6753 | 82 | Miraculin | 3 |
| 8426_0.path0_m.4449 | 148 | Miraculin | 3 |
| 8597_0.path0_m.4744 | 189 | Miraculin | 3 |
| 8426_0.path0_m.4449 | 175 | Miraculin | 3 |
| i0_LQ_c178185_f1p3_749_m.6753 | 107 | Miraculin | 3 |
| 7188_0.path0_m.3036 | 47 | Mitochondrial adenine nucleotide transporter ADNT1 | 3 |
| 6833_0.path1_m.2478 | 88 | Mitochondrial dicarboxylate_tricarboxylate transporter DTC | 1 |
| 6575_0.path0_m.2339 | 58 | Mitochondrial dicarboxylate_tricarboxylate transporter DTC | 1 |
| 6833_0.path1_m.2478 | 58 | Mitochondrial dicarboxylate_tricarboxylate transporter DTC | 2 |
| 8242_0.path1_m.4174 | 73 | Mitochondrial outer membrane protein porin of 34 kDa | 1 |
| 7360_0.path0_m.3297 | 271 | Mitochondrial outer membrane protein porin of 34 kDa | 1 |
| 7360_0.path0_m.3297 | 71 | Mitochondrial outer membrane protein porin of 34 kDa | 2 |
| 7360_0.path0_m.3297 | 36 | Mitochondrial outer membrane protein porin of 34 kDa | 2 |
| 7360_0.path0_m.3297 | 84 | Mitochondrial outer membrane protein porin of 34 kDa | 3 |
| 7360_0.path0_m.3297 | 120 | Mitochondrial outer membrane protein porin of 34 kDa | 3 |
| 7360_0.path0_m.3297 | 259 | Mitochondrial outer membrane protein porin of 34 kDa | 2 |
| 7360_0.path0_m.3297 | 70 | Mitochondrial outer membrane protein porin of 34 kDa | 1 |
| i1_HQ_c9639_f2p1_1104_m.1587 | 13 | Mitochondrial outer membrane protein porin of 34 kDa | 3 |
| 8752_0.path0_m.4971 | 249 | Mitochondrial phosphate carrier protein 3, mitochondrial | 3 |
| 8752_0.path0_m.4971 | 144 | Mitochondrial phosphate carrier protein 3, mitochondrial | 2 |
| 8752_0.path0_m.4971 | 348 | Mitochondrial phosphate carrier protein 3, mitochondrial | 1 |
| 6063_0.path0_m.1482 | 244 | Mitochondrial phosphate carrier protein 3, mitochondrial | 3 |
| 8752_0.path0_m.4971 | 252 | Mitochondrial phosphate carrier protein 3, mitochondrial | 3 |
| 10063_0.path0_m.88 | 126 | Mitochondrial-processing peptidase subunit alpha | 1 |
| 7306_0.path0_m.3205 | 127 | Mitochondrial-processing peptidase subunit alpha | 2 |
| i1_LQ_c88014_f1p0_1008_m.4205 | 246 | Mitochondrial-processing peptidase subunit alpha | 1 |
| 9658_0.path1_m.6076 | 85 | Monodehydroascorbate reductase | 3 |
| 8051_0.path0_m.4044 | 269 | Monodehydroascorbate reductase | 1 |
| 8051_0.path0_m.4044 | 30 | Monodehydroascorbate reductase | 3 |
| 9658_0.path1_m.6076 | 221 | Monodehydroascorbate reductase | 1 |
| i5_LQ_c4830_f1p0_5090_m.7265 | 90 | Movement protein Hsp70h | 1 |
| i5_LQ_c4830_f1p0_5090_m.7265 | 48 | Movement protein Hsp70h | 3 |
| 1563_0.path0_m.3776 | 143 | Movement protein Hsp70h | 1 |
| i5_LQ_c4830_f1p0_5090_m.7265 | 36 | Movement protein Hsp70h | 3 |
| i5_LQ_c4830_f1p0_5090_m.7265 | 89 | Movement protein Hsp70h | 3 |
| 1563_0.path0_m.3776 | 169 | Movement protein Hsp70h | 1 |
| i5_LQ_c4830_f1p0_5090_m.7265 | 38 | Movement protein Hsp70h | 3 |
| i5_LQ_c4830_f1p0_5090_m.7265 | 73 | Movement protein Hsp70h | 3 |
| i5_LQ_c4830_f1p0_5090_m.7265 | 139 | Movement protein Hsp70h | 3 |
| 1563_0.path0_m.3776 | 163 | Movement protein Hsp70h | 1 |
| 5339_0.path5_m.452 | 279 | N-acetyl-alpha-D-glucosaminyl L-malate synthase | 1 |
| 7464_0.path0_m.3180 | 21 | NAD(P)H:quinone oxidoreductase | 1 |
| 3773_0.path1_m.6408 | 229 | NAD-dependent malic enzyme 59 kDa isoform, mitochondrial | 3 |
| 3773_0.path1_m.6408 | 405 | NAD-dependent malic enzyme 59 kDa isoform, mitochondrial | 1 |
| 3879_0.path0_m.6640 | 299 | NAD-dependent malic enzyme 62 kDa isoform, mitochondrial | 3 |
| 3879_0.path0_m.6640 | 99 | NAD-dependent malic enzyme 62 kDa isoform, mitochondrial | 3 |
| 4700_0.path0_m.7851 | 97 | NADH dehydrogenase [ubiquinone] flavoprotein 1, mitochondrial | 3 |
| 4700_0.path0_m.7851 | 221 | NADH dehydrogenase [ubiquinone] flavoprotein 1, mitochondrial | 1 |
| 4700_0.path0_m.7851 | 124 | NADH dehydrogenase [ubiquinone] flavoprotein 1, mitochondrial | 2 |
| i1_HQ_c60867_f7p0_1132_m.308 | 148 | NADH dehydrogenase [ubiquinone] flavoprotein 2, mitochondrial | 2 |
| 9679_0.path0_m.6099 | 45 | NADH-cytochrome b5 reductase-like protein | 3 |
| 9679_0.path0_m.6099 | 83 | NADH-cytochrome b5 reductase-like protein | 1 |
| 9679_0.path0_m.6099 | 220 | NADH-cytochrome b5 reductase-like protein | 2 |
| 4476_0.path1_m.7434 | 411 | NADP-dependent malic enzyme | 3 |
| 9952_0.path0_m.6375 | 9 | Nascent polypeptide-associated complex subunit beta | 3 |
| i2_LQ_c70037_f1p0_2240_m.6643 | 215 | Natterin-4 | 1 |
| 5156_0.path0_m.146 | 222 | Natterin-4 | 1 |
| i2_LQ_c70037_f1p0_2240_m.6643 | 221 | Natterin-4 | 1 |
| 5156_0.path0_m.146 | 216 | Natterin-4 | 1 |
| i2_LQ_c70037_f1p0_2240_m.6643 | 123 | Natterin-4 | 2 |
| 6929_0.path0_m.2650 | 215 | Natterin-4 | 1 |
| 6929_0.path0_m.2650 | 221 | Natterin-4 | 1 |
| 5156_0.path0_m.146 | 124 | Natterin-4 | 2 |
| 6929_0.path0_m.2650 | 123 | Natterin-4 | 1 |
| 6929_0.path0_m.2650 | 164 | Natterin-4 | 1 |
| 5156_0.path0_m.146 | 60 | Natterin-4 | 2 |
| 11240_0.path0_m.1414 | 104 | Non-specific lipid-transfer protein 1 | 1 |
| 11240_0.path0_m.1414 | 76 | Non-specific lipid-transfer protein 1 | 3 |
| i0_HQ_c709_f39p6_690_m.5562 | 80 | Non-specific lipid-transfer protein 2 | 3 |
| 7090_0.path1_m.2911 | 178 | Nucleoside diphosphate kinase IV, chloroplastic_mitochondrial | 3 |
| 5367_0.path7_m.492 | 116 | Oryzain alpha chain | 1 |
| 6598_0.path0_m.2118 | 120 | Oxygen-evolving enhancer protein 1, chloroplastic | 3 |
| 6598_0.path0_m.2118 | 90 | Oxygen-evolving enhancer protein 1, chloroplastic | 3 |
| 6598_0.path0_m.2118 | 213 | Oxygen-evolving enhancer protein 1, chloroplastic | 3 |
| 6598_0.path0_m.2118 | 125 | Oxygen-evolving enhancer protein 1, chloroplastic | 3 |
| 6598_0.path0_m.2118 | 113 | Oxygen-evolving enhancer protein 1, chloroplastic | 3 |
| 6598_0.path0_m.2118 | 136 | Oxygen-evolving enhancer protein 1, chloroplastic | 3 |
| 6598_0.path0_m.2118 | 235 | Oxygen-evolving enhancer protein 1, chloroplastic | 2 |
| 6598_0.path0_m.2118 | 181 | Oxygen-evolving enhancer protein 1, chloroplastic | 3 |
| 7619_0.path0_m.3408 | 112 | Oxygen-evolving enhancer protein 2-1, chloroplastic | 3 |
| 7619_0.path0_m.3408 | 231 | Oxygen-evolving enhancer protein 2-1, chloroplastic | 3 |
| 7619_0.path0_m.3408 | 239 | Oxygen-evolving enhancer protein 2-1, chloroplastic | 2 |
| 7942_0.path0_m.3874 | 224 | Oxygen-evolving enhancer protein 3, chloroplastic | 1 |
| 7942_0.path0_m.3874 | 213 | Oxygen-evolving enhancer protein 3, chloroplastic | 1 |
| i0_LQ_c128861_f1p0_857_m.7234 | 88 | Oxygen-evolving enhancer protein 3-1, chloroplastic | 2 |
| i0_LQ_c128861_f1p0_857_m.7234 | 155 | Oxygen-evolving enhancer protein 3-1, chloroplastic | 3 |
| i0_LQ_c128861_f1p0_857_m.7234 | 26 | Oxygen-evolving enhancer protein 3-1, chloroplastic | 2 |
| i0_LQ_c128861_f1p0_857_m.7234 | 115 | Oxygen-evolving enhancer protein 3-1, chloroplastic | 3 |
| i0_LQ_c128861_f1p0_857_m.7234 | 129 | Oxygen-evolving enhancer protein 3-1, chloroplastic | 3 |
| i0_LQ_c128861_f1p0_857_m.7234 | 132 | Oxygen-evolving enhancer protein 3-1, chloroplastic | 3 |
| i0_LQ_c128861_f1p0_857_m.7234 | 82 | Oxygen-evolving enhancer protein 3-1, chloroplastic | 3 |
| i0_LQ_c128861_f1p0_857_m.7234 | 166 | Oxygen-evolving enhancer protein 3-1, chloroplastic | 3 |
| i0_LQ_c128861_f1p0_857_m.7234 | 76 | Oxygen-evolving enhancer protein 3-1, chloroplastic | 3 |
| i0_LQ_c128861_f1p0_857_m.7234 | 117 | Oxygen-evolving enhancer protein 3-1, chloroplastic | 3 |
| 8276_0.path0_m.4221 | 230 | Patchoulol synthase | 1 |
| 8276_0.path0_m.4221 | 233 | Patchoulol synthase | 3 |
| 8276_0.path0_m.4221 | 68 | Patchoulol synthase | 3 |
| 10399_0.path0_m.584 | 89 | Pathogenesis-related leaf protein 6 | 3 |
| 10399_0.path0_m.584 | 107 | Pathogenesis-related leaf protein 6 | 3 |
| 4427_0.path0_m.7313 | 102 | Pectinesterase_pectinesterase inhibitor 3 | 2 |
| i1_LQ_c27272_f1p0_1804_m.5221 | 468 | Pectinesterase_pectinesterase inhibitor 3 | 3 |
| i1_LQ_c27272_f1p0_1804_m.5221 | 91 | Pectinesterase_pectinesterase inhibitor 3 | 3 |
| 5700_0.path0_m.1129 | 5 | Pentatricopeptide repeat-containing protein At5g12100, mitochondrial | 1 |
| 11226_0.path0_m.1398 | 124 | Peptide methionine sulfoxide reductase | 2 |
| 11470_0.path0_m.1680 | 150 | Peptide methionine sulfoxide reductase (Fragment) | 2 |
| 6193_0.path0_m.1692 | 405 | Peptide-N4-(N-acetyl-beta-glucosaminyl)asparagine amidase A | 1 |
| 6193_0.path0_m.1692 | 540 | Peptide-N4-(N-acetyl-beta-glucosaminyl)asparagine amidase A | 1 |
| 8552_0.path0_m.4687 | 147 | Peptidyl-prolyl cis-trans isomerase 1 | 1 |
| 9407_0.path0_m.5701 | 58 | Peptidyl-prolyl cis-trans isomerase FKBP12 | 1 |
| i1_LQ_c41952_f1p0_1255_m.178 | 225 | Peptidyl-prolyl cis-trans isomerase FKBP62 | 1 |
| 8418_0.path0_m.4439 | 115 | Peroxidase 4 | 3 |
| 8418_0.path0_m.4439 | 160 | Peroxidase 4 | 2 |
| i1_HQ_c17945_f4p0_1122_m.1124 | 60 | Peroxidase 4 | 2 |
| 8418_0.path0_m.4439 | 12 | Peroxidase 4 | 1 |
| i0_LQ_c391587_f1p0_639_m.4102 | 125 | Peroxiredoxin Q, chloroplastic | 2 |
| i0_LQ_c391587_f1p0_639_m.4102 | 163 | Peroxiredoxin Q, chloroplastic | 3 |
| i0_LQ_c391587_f1p0_639_m.4102 | 122 | Peroxiredoxin Q, chloroplastic | 3 |
| 8824_0.path0_m.5082 | 126 | Peroxiredoxin-2B | 2 |
| 8824_0.path0_m.5082 | 118 | Peroxiredoxin-2B | 3 |
| 5827_0.path3_m.1058 | 151 | Peroxisomal (S)-2-hydroxy-acid oxidase GLO1 | 3 |
| i0_LQ_c104330_f1p1_837_m.4398 | 221 | Peroxisomal (S)-2-hydroxy-acid oxidase GLO2 | 3 |
| 10165_0.path0_m.247 | 440 | Peroxisomal acyl-coenzyme A oxidase 1 | 2 |
| 3152_0.path2_m.5448 | 211 | Phosphatidylinositol_phosphatidylcholine transfer protein SFH8 | 1 |
| 4658_0.path0_m.7768 | 124 | Phosphoglycerate kinase, chloroplastic | 1 |
| 4658_0.path0_m.7768 | 148 | Phosphoglycerate kinase, chloroplastic | 3 |
| i1_LQ_c67740_f1p0_1601_m.6729 | 78 | Phosphoglycerate kinase, cytosolic | 2 |
| i1_LQ_c67740_f1p0_1601_m.6729 | 74 | Phosphoglycerate kinase, cytosolic | 3 |
| i1_LQ_c67740_f1p0_1601_m.6729 | 261 | Phosphoglycerate kinase, cytosolic | 1 |
| i1_LQ_c67740_f1p0_1601_m.6729 | 168 | Phosphoglycerate kinase, cytosolic | 2 |
| 9469_0.path0_m.5810 | 128 | Phosphoglycolate phosphatase 1A, chloroplastic | 1 |
| 5634_0.path0_m.1004 | 287 | Phospholipase A1-IIdelta | 3 |
| 5634_0.path0_m.1004 | 202 | Phospholipase A1-IIdelta | 3 |
| 5634_0.path0_m.1004 | 363 | Phospholipase A1-IIdelta | 2 |
| 5634_0.path0_m.1004 | 381 | Phospholipase A1-IIdelta | 3 |
| 5634_0.path0_m.1004 | 80 | Phospholipase A1-IIdelta | 3 |
| 5634_0.path0_m.1004 | 217 | Phospholipase A1-IIdelta | 3 |
| 5634_0.path0_m.1004 | 270 | Phospholipase A1-IIdelta | 2 |
| 5634_0.path0_m.1004 | 162 | Phospholipase A1-IIdelta | 1 |
| 10590_0.path0_m.838 | 163 | Photosynthetic NDH subunit of lumenal location 5, chloroplastic | 1 |
| 5071_0.path1_m.1 | 162 | Photosystem I P700 chlorophyll a apoprotein A2 | 1 |
| 5071_0.path1_m.2 | 211 | Photosystem I P700 chlorophyll a apoprotein A2 | 2 |
| 5071_0.path1_m.2 | 68 | Photosystem I P700 chlorophyll a apoprotein A2 | 3 |
| 8810_0.path5_m.5063 | 76 | Photosystem I reaction center subunit II, chloroplastic | 2 |
| 8810_0.path5_m.5063 | 95 | Photosystem I reaction center subunit II, chloroplastic | 3 |
| 8810_0.path5_m.5063 | 139 | Photosystem I reaction center subunit II, chloroplastic | 3 |
| 8810_0.path5_m.5063 | 147 | Photosystem I reaction center subunit II, chloroplastic | 3 |
| 7914_0.path0_m.3834 | 96 | Photosystem I reaction center subunit III, chloroplastic | 3 |
| 7914_0.path0_m.3834 | 127 | Photosystem I reaction center subunit III, chloroplastic | 3 |
| 7914_0.path0_m.3834 | 99 | Photosystem I reaction center subunit III, chloroplastic | 2 |
| 7914_0.path0_m.3834 | 117 | Photosystem I reaction center subunit III, chloroplastic | 3 |
| 9127_0.path0_m.5312 | 76 | Photosystem I reaction center subunit IV, chloroplastic | 2 |
| 9127_0.path0_m.5312 | 99 | Photosystem I reaction center subunit IV, chloroplastic | 3 |
| i0_HQ_c48389_f3p13_732_m.5398 | 135 | Photosystem I reaction center subunit N, chloroplastic | 2 |
| i0_HQ_c48389_f3p13_732_m.5398 | 154 | Photosystem I reaction center subunit N, chloroplastic | 2 |
| i0_LQ_c63447_f1p14_724_m.5286 | 85 | Photosystem I reaction center subunit psaK, chloroplastic | 2 |
| 9654_0.path0_m.6072 | 111 | Photosystem I reaction center subunit V, chloroplastic | 3 |
| 8472_0.path0_m.4524 | 137 | Photosystem I reaction center subunit VI, chloroplastic | 3 |
| 8472_0.path0_m.4524 | 98 | Photosystem I reaction center subunit VI, chloroplastic | 3 |
| i0_LQ_c102321_f1p1_510_m.5175 | 24 | Photosystem II 10 kDa polypeptide, chloroplastic | 1 |
| i0_LQ_c102321_f1p1_510_m.5175 | 51 | Photosystem II 10 kDa polypeptide, chloroplastic | 3 |
| 10229_0.path0_m.317 | 98 | Photosystem II 5 kDa protein, chloroplastic | 3 |
| 1315_0.path3_m.3128 | 7 | Photosystem II CP43 reaction center protein | 2 |
| 1315_0.path3_m.3128 | 318 | Photosystem II CP43 reaction center protein | 2 |
| 5136_0.path0_m.111 | 308 | Photosystem II CP47 reaction center protein | 3 |
| 5136_0.path0_m.111 | 438 | Photosystem II CP47 reaction center protein | 3 |
| 5136_0.path0_m.111 | 304 | Photosystem II CP47 reaction center protein | 3 |
| 4155_0.path0_m.7150 | 316 | Plasma membrane ATPase 1 | 1 |
| 6462_0.path16_m.2160 | 177 | Plasma membrane ATPase 4 | 1 |
| 8318_0.path0_m.4286 | 86 | Plasma membrane-associated cation-binding protein 1 | 1 |
| i0_HQ_c28425_f2p22_869_m.4307 | 97 | Plastocyanin, chloroplastic | 3 |
| 7569_0.path0_m.3334 | 258 | Polygalacturonase inhibitor | 2 |
| i1_HQ_c40190_f2p0_1279_m.7400 | 43 | Polygalacturonase inhibitor | 1 |
| i1_HQ_c40190_f2p0_1279_m.7400 | 282 | Polygalacturonase inhibitor | 2 |
| i1_HQ_c40190_f2p0_1279_m.7400 | 275 | Polygalacturonase inhibitor | 3 |
| i1_HQ_c40190_f2p0_1279_m.7400 | 124 | Polygalacturonase inhibitor | 2 |
| 7569_0.path0_m.3334 | 315 | Polygalacturonase inhibitor | 1 |
| i1_HQ_c40190_f2p0_1279_m.7400 | 302 | Polygalacturonase inhibitor | 2 |
| i1_HQ_c40190_f2p0_1279_m.7400 | 321 | Polygalacturonase inhibitor | 2 |
| i1_HQ_c40190_f2p0_1279_m.7400 | 42 | Polygalacturonase inhibitor | 2 |
| i1_HQ_c40190_f2p0_1279_m.7400 | 264 | Polygalacturonase inhibitor | 3 |
| i1_HQ_c40190_f2p0_1279_m.7400 | 228 | Polygalacturonase inhibitor | 2 |
| i1_HQ_c40190_f2p0_1279_m.7400 | 180 | Polygalacturonase inhibitor | 2 |
| 5737_0.path0_m.1203 | 572 | Polyphenol oxidase I, chloroplastic | 3 |
| 5737_0.path0_m.1203 | 562 | Polyphenol oxidase I, chloroplastic | 3 |
| 5737_0.path0_m.1203 | 409 | Polyphenol oxidase I, chloroplastic | 2 |
| 5737_0.path0_m.1203 | 511 | Polyphenol oxidase I, chloroplastic | 3 |
| 5737_0.path0_m.1203 | 491 | Polyphenol oxidase I, chloroplastic | 3 |
| 5737_0.path0_m.1203 | 126 | Polyphenol oxidase I, chloroplastic | 3 |
| 5737_0.path0_m.1203 | 134 | Polyphenol oxidase I, chloroplastic | 3 |
| 5737_0.path0_m.1203 | 484 | Polyphenol oxidase I, chloroplastic | 3 |
| 5737_0.path0_m.1203 | 305 | Polyphenol oxidase I, chloroplastic | 3 |
| 5737_0.path0_m.1203 | 524 | Polyphenol oxidase I, chloroplastic | 3 |
| 5737_0.path0_m.1203 | 451 | Polyphenol oxidase I, chloroplastic | 2 |
| 5532_0.path0_m.831 | 458 | Polyphenol oxidase I, chloroplastic | 1 |
| 5532_0.path0_m.831 | 22 | Polyphenol oxidase I, chloroplastic | 3 |
| 5737_0.path0_m.1203 | 149 | Polyphenol oxidase I, chloroplastic | 3 |
| 4933_0.path0_m.8308 | 119 | Polyphenol oxidase II, chloroplastic | 1 |
| 4933_0.path0_m.8308 | 137 | Polyphenol oxidase II, chloroplastic | 3 |
| 4933_0.path0_m.8308 | 460 | Polyphenol oxidase II, chloroplastic | 3 |
| 4933_0.path0_m.8308 | 447 | Polyphenol oxidase II, chloroplastic | 3 |
| 4933_0.path0_m.8308 | 145 | Polyphenol oxidase II, chloroplastic | 3 |
| 4933_0.path0_m.8308 | 427 | Polyphenol oxidase II, chloroplastic | 3 |
| 4202_0.path1_m.7266 | 528 | Polyphenol oxidase II, chloroplastic | 3 |
| 4202_0.path1_m.7266 | 309 | Polyphenol oxidase II, chloroplastic | 3 |
| 4202_0.path1_m.7266 | 583 | Polyphenol oxidase II, chloroplastic | 3 |
| 4202_0.path1_m.7266 | 442 | Polyphenol oxidase II, chloroplastic | 3 |
| 4933_0.path0_m.8308 | 262 | Polyphenol oxidase II, chloroplastic | 3 |
| 4933_0.path0_m.8308 | 419 | Polyphenol oxidase II, chloroplastic | 3 |
| 4202_0.path1_m.7266 | 457 | Polyphenol oxidase II, chloroplastic | 2 |
| 4202_0.path1_m.7266 | 412 | Polyphenol oxidase II, chloroplastic | 2 |
| 4202_0.path1_m.7266 | 155 | Polyphenol oxidase II, chloroplastic | 1 |
| 4202_0.path1_m.7266 | 576 | Polyphenol oxidase II, chloroplastic | 3 |
| 4202_0.path1_m.7266 | 140 | Polyphenol oxidase II, chloroplastic | 3 |
| 4933_0.path0_m.8308 | 437 | Polyphenol oxidase II, chloroplastic | 2 |
| 4933_0.path0_m.8308 | 151 | Polyphenol oxidase II, chloroplastic | 3 |
| 4933_0.path0_m.8308 | 249 | Polyphenol oxidase II, chloroplastic | 3 |
| 4933_0.path0_m.8308 | 453 | Polyphenol oxidase II, chloroplastic | 3 |
| 6834_0.path0_m.2479 | 11 | Polyubiquitin | 1 |
| 6834_0.path0_m.2479 | 63 | Polyubiquitin | 1 |
| 6834_0.path0_m.2479 | 6 | Polyubiquitin | 1 |
| 6834_0.path0_m.2479 | 48 | Polyubiquitin | 1 |
| 6515_0.path2_m.2241 | 430 | Primary amine oxidase | 2 |
| i1_LQ_c77746_f1p0_1530_m.131 | 223 | Probable acetyl-CoA acetyltransferase, cytosolic 2 | 3 |
| i1_LQ_c20662_f1p0_1805_m.5359 | 385 | Probable aldehyde dehydrogenase | 2 |
| 7342_0.path0_m.3268 | 12 | Probable aldo-keto reductase 4 | 2 |
| 7342_0.path0_m.3268 | 147 | Probable aldo-keto reductase 4 | 2 |
| 3129_0.path0_m.5386 | 163 | Probable alpha-mannosidase At5g13980 | 3 |
| 3129_0.path0_m.5386 | 848 | Probable alpha-mannosidase At5g13980 | 1 |
| 8789_0.path0_m.5031 | 100 | Probable ATP synthase 24 kDa subunit, mitochondrial | 3 |
| 8789_0.path0_m.5031 | 55 | Probable ATP synthase 24 kDa subunit, mitochondrial | 3 |
| 8789_0.path0_m.5031 | 25 | Probable ATP synthase 24 kDa subunit, mitochondrial | 3 |
| 6636_0.path1_m.2179 | 222 | Probable carboxylesterase 8 | 3 |
| 6636_0.path1_m.2179 | 90 | Probable carboxylesterase 8 | 2 |
| i1_LQ_c24465_f1p2_1115_m.4161 | 223 | Probable carboxylesterase 8 | 3 |
| i1_LQ_c24465_f1p2_1115_m.4161 | 89 | Probable carboxylesterase 8 | 1 |
| 6636_0.path1_m.2179 | 277 | Probable carboxylesterase 8 | 1 |
| 6068_0.path5_m.1490 | 256 | Probable elongation factor 1-gamma 2 | 1 |
| i1_LQ_c46534_f1p0_1143_m.1204 | 311 | Probable enoyl-CoA hydratase 2, mitochondrial | 1 |
| 10612_0.path0_m.864 | 64 | Probable enoyl-CoA hydratase 2, mitochondrial | 3 |
| 10612_0.path0_m.864 | 291 | Probable enoyl-CoA hydratase 2, mitochondrial | 2 |
| i1_LQ_c46534_f1p0_1143_m.1204 | 131 | Probable enoyl-CoA hydratase 2, mitochondrial | 3 |
| i1_LQ_c46534_f1p0_1143_m.1204 | 84 | Probable enoyl-CoA hydratase 2, mitochondrial | 1 |
| 10612_0.path0_m.864 | 111 | Probable enoyl-CoA hydratase 2, mitochondrial | 3 |
| 8826_0.path0_m.5084 | 223 | Probable glucan 1,3-beta-glucosidase A | 3 |
| 8826_0.path0_m.5084 | 107 | Probable glucan 1,3-beta-glucosidase A | 3 |
| 8826_0.path0_m.5084 | 306 | Probable glucan 1,3-beta-glucosidase A | 1 |
| 6127_0.path0_m.1593 | 269 | Probable glucan endo-1,3-beta-glucosidase A6 | 2 |
| 6127_0.path0_m.1594 | 52 | Probable glucan endo-1,3-beta-glucosidase A6 | 2 |
| 8640_0.path0_m.4809 | 128 | Probable glutathione S-transferase | 2 |
| 6763_0.path0_m.2378 | 47 | Probable mitochondrial-processing peptidase subunit beta, mitochondrial | 2 |
| 6763_0.path0_m.2378 | 38 | Probable mitochondrial-processing peptidase subunit beta, mitochondrial | 3 |
| i1_HQ_c20333_f2p0_1924_m.2340 | 253 | Probable mitochondrial-processing peptidase subunit beta, mitochondrial | 2 |
| i1_LQ_c90359_f1p0_1008_m.5193 | 185 | Probable NAD(P)H dehydrogenase (quinone) FQR1-like 1 | 2 |
| 7493_0.path1_m.3233 | 197 | Probable NAD(P)H dehydrogenase (quinone) FQR1-like 1 | 3 |
| i1_LQ_c90359_f1p0_1008_m.5193 | 223 | Probable NAD(P)H dehydrogenase (quinone) FQR1-like 1 | 3 |
| i1_LQ_c90359_f1p0_1008_m.5193 | 216 | Probable NAD(P)H dehydrogenase (quinone) FQR1-like 1 | 3 |
| 8541_0.path0_m.4672 | 165 | Probable NADH dehydrogenase [ubiquinone] 1 alpha subcomplex subunit 5, mitochondrial | 1 |
| i0_LQ_c58068_f1p1_942_m.5321 | 122 | Probable phospholipid hydroperoxide glutathione peroxidase | 1 |
| i0_LQ_c58068_f1p1_942_m.5321 | 95 | Probable phospholipid hydroperoxide glutathione peroxidase | 2 |
| i0_LQ_c58068_f1p1_942_m.5321 | 160 | Probable phospholipid hydroperoxide glutathione peroxidase | 2 |
| 7413_0.path0_m.3113 | 231 | Probable plastid-lipid-associated protein 6, chloroplastic | 1 |
| 7413_0.path0_m.3113 | 235 | Probable plastid-lipid-associated protein 6, chloroplastic | 1 |
| 7413_0.path0_m.3113 | 97 | Probable plastid-lipid-associated protein 6, chloroplastic | 3 |
| 8465_0.path0_m.4513 | 245 | Probable S-adenosylmethionine-dependent methyltransferase At5g37990 | 3 |
| i2_LQ_c55751_f1p0_2961_m.1622 | 144 | Probable serine_threonine protein kinase IREH1 | 1 |
| 8355_0.path0_m.4350 | 104 | Profilin-3 | 3 |
| 8355_0.path0_m.4350 | 80 | Profilin-3 | 2 |
| 6863_0.path1_m.2523 | 4 | Prohibitin-1, mitochondrial | 2 |
| 6863_0.path1_m.2523 | 100 | Prohibitin-1, mitochondrial | 2 |
| 11450_0.path0_m.1663 | 139 | Prohibitin-3, mitochondrial | 3 |
| 10956_0.path0_m.1142 | 102 | Proteasome subunit alpha type-2-A | 2 |
| 7075_0.path1_m.2886 | 455 | Protein ASPARTIC PROTEASE IN GUARD CELL 1 | 1 |
| 8671_0.path0_m.4850 | 186 | Protein COBRA | 3 |
| 8671_0.path0_m.4850 | 204 | Protein COBRA | 2 |
| 4563_0.path0_m.7582 | 418 | Protein DEK | 2 |
| i0_LQ_c5647_f1p0_879_m.2210 | 235 | Protein EXORDIUM | 1 |
| i1_LQ_c59710_f1p0_1057_m.5199 | 100 | Protein EXORDIUM-like 2 | 1 |
| i1_LQ_c36007_f1p0_1069_m.13 | 191 | Protein IN2-1 homolog B | 3 |
| i1_LQ_c36007_f1p0_1069_m.13 | 230 | Protein IN2-1 homolog B | 3 |
| i1_LQ_c36007_f1p0_1069_m.13 | 219 | Protein IN2-1 homolog B | 1 |
| i1_LQ_c36007_f1p0_1069_m.13 | 70 | Protein IN2-1 homolog B | 3 |
| i1_LQ_c36007_f1p0_1069_m.13 | 104 | Protein IN2-1 homolog B | 1 |
| i1_LQ_c36007_f1p0_1069_m.13 | 204 | Protein IN2-1 homolog B | 3 |
| 4525_0.path0_m.7522 | 288 | Protein NRT1_ PTR FAMILY 5.6 | 2 |
| 8286_0.path3_m.4240 | 60 | Protein plastid transcriptionally active 16, chloroplastic | 1 |
| 5281_0.path0_m.366 | 60 | Protein plastid transcriptionally active 16, chloroplastic | 2 |
| 5281_0.path0_m.366 | 492 | Protein plastid transcriptionally active 16, chloroplastic | 1 |
| 5281_0.path0_m.366 | 458 | Protein plastid transcriptionally active 16, chloroplastic | 1 |
| 3037_1.path0_m.5174 | 14 | Protein tesmin_TSO1-like CXC 2 | 2 |
| 4571_0.path0_m.7605 | 385 | Protein TolB | 1 |
| i0_LQ_c326122_f1p1_708_m.7228 | 79 | Protein YLS3 | 2 |
| i0_LQ_c326122_f1p1_708_m.7228 | 64 | Protein YLS3 | 3 |
| 1439_0.path0_m.3458 | 556 | Putative ABC transporter B family member 8 | 3 |
| i0_LQ_c149507_f1p2_991_m.6413 | 90 | Putative lactoylglutathione lyase | 1 |
| i0_LQ_c380498_f5p1_601_m.1077 | 108 | Putative peptidyl-tRNA hydrolase PTRHD1 | 1 |
| 7808_0.path0_m.3669 | 54 | Putative quinone-oxidoreductase homolog, chloroplastic | 3 |
| 7112_0.path0_m.2939 | 336 | Pyruvate dehydrogenase E1 component subunit alpha, mitochondrial | 1 |
| 7112_0.path0_m.2939 | 239 | Pyruvate dehydrogenase E1 component subunit alpha, mitochondrial | 3 |
| 7112_0.path0_m.2939 | 371 | Pyruvate dehydrogenase E1 component subunit alpha, mitochondrial | 3 |
| 7112_0.path0_m.2939 | 354 | Pyruvate dehydrogenase E1 component subunit alpha, mitochondrial | 3 |
| 7112_0.path0_m.2939 | 366 | Pyruvate dehydrogenase E1 component subunit alpha, mitochondrial | 1 |
| 7112_0.path0_m.2939 | 322 | Pyruvate dehydrogenase E1 component subunit alpha, mitochondrial | 3 |
| 7112_0.path0_m.2939 | 333 | Pyruvate dehydrogenase E1 component subunit alpha, mitochondrial | 3 |
| 9636_0.path0_m.6039 | 76 | Pyruvate dehydrogenase E1 component subunit beta-1, mitochondrial | 3 |
| i1_LQ_c47461_f2p0_1737_m.6781 | 498 | Pyruvate kinase, cytosolic isozyme | 1 |
| 5201_0.path1_m.207 | 31 | Raucaffricine-O-beta-D-glucosidase | 2 |
| 5201_0.path1_m.207 | 386 | Raucaffricine-O-beta-D-glucosidase | 3 |
| 10660_0.path0_m.916 | 144 | Reactive Intermediate Deaminase A, chloroplastic | 3 |
| i1_HQ_c20148_f7p0_1561_m.4141 | 248 | RGG repeats nuclear RNA binding protein A (Fragment) | 3 |
| 1899_0.path1_m.3813 | 789 | Ribonuclease J | 1 |
| 1899_0.path1_m.3813 | 794 | Ribonuclease J | 1 |
| 5638_0.path0_m.1009 | 153 | Ribulose bisphosphate carboxylase large chain | 3 |
| 5638_0.path0_m.1009 | 39 | Ribulose bisphosphate carboxylase large chain | 3 |
| 5638_0.path0_m.1009 | 259 | Ribulose bisphosphate carboxylase large chain | 1 |
| 5638_0.path0_m.1009 | 457 | Ribulose bisphosphate carboxylase large chain | 1 |
| 5638_0.path0_m.1009 | 243 | Ribulose bisphosphate carboxylase large chain | 2 |
| 5638_0.path0_m.1009 | 184 | Ribulose bisphosphate carboxylase large chain | 2 |
| 5638_0.path0_m.1009 | 182 | Ribulose bisphosphate carboxylase large chain | 3 |
| 5638_0.path0_m.1009 | 341 | Ribulose bisphosphate carboxylase large chain | 3 |
| 5638_0.path0_m.1009 | 208 | Ribulose bisphosphate carboxylase large chain | 1 |
| 5638_0.path0_m.1009 | 190 | Ribulose bisphosphate carboxylase large chain | 3 |
| 5638_0.path0_m.1009 | 323 | Ribulose bisphosphate carboxylase large chain | 2 |
| 8673_0.path2_m.4855 | 107 | Ribulose bisphosphate carboxylase small chain, chloroplastic | 3 |
| 8673_0.path2_m.4855 | 36 | Ribulose bisphosphate carboxylase small chain, chloroplastic | 1 |
| 8673_0.path2_m.4855 | 11 | Ribulose bisphosphate carboxylase small chain, chloroplastic | 3 |
| 4413_0.path0_m.7292 | 400 | Ribulose bisphosphate carboxylase_oxygenase activase 2, chloroplastic | 2 |
| i1_HQ_c2074_f3p0_1549_m.7677 | 434 | Ribulose bisphosphate carboxylase_oxygenase activase 2, chloroplastic | 2 |
| i1_HQ_c2074_f3p0_1549_m.7677 | 173 | Ribulose bisphosphate carboxylase_oxygenase activase 2, chloroplastic | 1 |
| i1_HQ_c2074_f3p0_1549_m.7677 | 398 | Ribulose bisphosphate carboxylase_oxygenase activase 2, chloroplastic | 3 |
| i1_HQ_c2074_f3p0_1549_m.7677 | 328 | Ribulose bisphosphate carboxylase_oxygenase activase 2, chloroplastic | 3 |
| i1_HQ_c2074_f3p0_1549_m.7677 | 177 | Ribulose bisphosphate carboxylase_oxygenase activase 2, chloroplastic | 1 |
| 4413_0.path0_m.7292 | 410 | Ribulose bisphosphate carboxylase_oxygenase activase 2, chloroplastic | 1 |
| 4413_0.path0_m.7292 | 396 | Ribulose bisphosphate carboxylase_oxygenase activase 2, chloroplastic | 1 |
| i1_HQ_c2074_f3p0_1549_m.7677 | 394 | Ribulose bisphosphate carboxylase_oxygenase activase 2, chloroplastic | 2 |
| i1_LQ_c69489_f1p0_1162_m.2162 | 168 | Ricin B-like lectin EULS3 | 1 |
| 10269_0.path1_m.386 | 333 | RNA2 polyprotein | 1 |
| 1866_0.path5_m.3741 | 355 | RNA2 polyprotein | 2 |
| 1866_0.path5_m.3741 | 751 | RNA2 polyprotein | 2 |
| 10269_0.path1_m.386 | 190 | RNA2 polyprotein | 1 |
| 1866_0.path5_m.3741 | 605 | RNA2 polyprotein | 2 |
| 10269_0.path1_m.386 | 224 | RNA2 polyprotein | 1 |
| 10269_0.path1_m.386 | 474 | RNA2 polyprotein | 1 |
| 1866_0.path5_m.3741 | 321 | RNA2 polyprotein | 2 |
| 6835_0.path4_m.2482 | 113 | RNA2 polyprotein | 2 |
| 6835_0.path4_m.2482 | 120 | RNA2 polyprotein | 2 |
| 4485_0.path0_m.7451 | 419 | RuBisCO large subunit-binding protein subunit alpha, chloroplastic | 3 |
| 5436_0.path0_m.624 | 371 | RuBisCO large subunit-binding protein subunit alpha, chloroplastic | 1 |
| 4485_0.path0_m.7451 | 165 | RuBisCO large subunit-binding protein subunit alpha, chloroplastic | 1 |
| i2_LQ_c49747_f1p2_2285_m.3131 | 287 | RuBisCO large subunit-binding protein subunit beta, chloroplastic | 3 |
| i2_LQ_c49747_f1p2_2285_m.3131 | 195 | RuBisCO large subunit-binding protein subunit beta, chloroplastic | 1 |
| i2_LQ_c49747_f1p2_2285_m.3131 | 339 | RuBisCO large subunit-binding protein subunit beta, chloroplastic | 3 |
| i2_LQ_c49747_f1p2_2285_m.3131 | 487 | RuBisCO large subunit-binding protein subunit beta, chloroplastic | 1 |
| i2_LQ_c49747_f1p2_2285_m.3131 | 516 | RuBisCO large subunit-binding protein subunit beta, chloroplastic | 3 |
| i2_LQ_c49747_f1p2_2285_m.3131 | 97 | RuBisCO large subunit-binding protein subunit beta, chloroplastic | 1 |
| i2_LQ_c49747_f1p2_2285_m.3131 | 188 | RuBisCO large subunit-binding protein subunit beta, chloroplastic | 3 |
| i2_LQ_c49747_f1p2_2285_m.3131 | 334 | RuBisCO large subunit-binding protein subunit beta, chloroplastic | 2 |
| 7646_0.path0_m.3435 | 323 | S-adenosylmethionine synthase 1 | 2 |
| 7646_0.path0_m.3435 | 219 | S-adenosylmethionine synthase 1 | 2 |
| 7646_0.path0_m.3435 | 356 | S-adenosylmethionine synthase 1 | 1 |
| 7646_0.path0_m.3435 | 214 | S-adenosylmethionine synthase 1 | 1 |
| 11384_0.path0_m.1588 | 308 | Sedoheptulose-1,7-bisphosphatase, chloroplastic | 3 |
| 11384_0.path0_m.1588 | 369 | Sedoheptulose-1,7-bisphosphatase, chloroplastic | 2 |
| 9208_0.path0_m.5428 | 90 | Seed lectin | 3 |
| 9208_0.path0_m.5428 | 64 | Seed lectin | 3 |
| 9208_0.path0_m.5428 | 86 | Seed lectin | 3 |
| i1_LQ_c11136_f1p2_1950_m.5633 | 399 | Serine carboxypeptidase 1 | 1 |
| i1_LQ_c53538_f1p0_1993_m.4442 | 166 | Serine carboxypeptidase-like 29 | 2 |
| i1_LQ_c53538_f1p0_1993_m.4442 | 447 | Serine carboxypeptidase-like 29 | 1 |
| i0_LQ_c51764_f1p4_562_m.81 | 83 | Serine hydroxymethyltransferase 4 | 1 |
| 9685_0.path0_m.6107 | 185 | Serine hydroxymethyltransferase, mitochondrial | 3 |
| 9685_0.path0_m.6107 | 412 | Serine hydroxymethyltransferase, mitochondrial | 3 |
| 9685_0.path0_m.6107 | 507 | Serine hydroxymethyltransferase, mitochondrial | 3 |
| 9685_0.path0_m.6107 | 401 | Serine hydroxymethyltransferase, mitochondrial | 3 |
| 9685_0.path0_m.6107 | 471 | Serine hydroxymethyltransferase, mitochondrial | 3 |
| 10664_0.path0_m.935 | 77 | Soluble inorganic pyrophosphatase 6, chloroplastic | 2 |
| 10664_0.path0_m.936 | 276 | Soluble inorganic pyrophosphatase 6, chloroplastic | 2 |
| i0_LQ_c141836_f1p4_434_m.1649 | 68 | Sorbitol dehydrogenase | 2 |
| 11224_0.path0_m.1395 | 338 | Stomatin-like protein 2, mitochondrial | 3 |
| i1_LQ_c8279_f1p0_1816_m.2140 | 172 | Stromal 70 kDa heat shock-related protein, chloroplastic | 1 |
| 3338_0.path7_m.5835 | 413 | Stromal 70 kDa heat shock-related protein, chloroplastic | 1 |
| 3338_0.path7_m.5835 | 119 | Stromal 70 kDa heat shock-related protein, chloroplastic | 2 |
| 2876_0.path0_m.5382 | 118 | Stromal 70 kDa heat shock-related protein, chloroplastic | 1 |
| 3338_0.path7_m.5835 | 108 | Stromal 70 kDa heat shock-related protein, chloroplastic | 1 |
| 9798_0.path6_m.6274 | 424 | Structural maintenance of chromosomes protein 3 | 2 |
| 3998_0.path0_m.6832 | 752 | Subtilisin-like protease SBT1.6 | 1 |
| i2_LQ_c21648_f1p0_2342_m.5165 | 327 | Subtilisin-like protease SBT1.6 | 1 |
| i2_LQ_c21648_f1p0_2342_m.5165 | 306 | Subtilisin-like protease SBT1.6 | 3 |
| i2_LQ_c33479_f1p0_2471_m.3092 | 420 | Subtilisin-like protease SBT1.9 | 3 |
| i2_LQ_c33479_f1p0_2471_m.3092 | 718 | Subtilisin-like protease SBT1.9 | 3 |
| i2_LQ_c33479_f1p0_2471_m.3092 | 752 | Subtilisin-like protease SBT1.9 | 3 |
| 9144_0.path0_m.5334 | 55 | Subtilisin-like protease SBT5.6 | 1 |
| 4897_0.path0_m.8229 | 318 | Succinate dehydrogenase [ubiquinone] flavoprotein subunit 1, mitochondrial | 3 |
| 4897_0.path0_m.8229 | 464 | Succinate dehydrogenase [ubiquinone] flavoprotein subunit 1, mitochondrial | 2 |
| 4897_0.path0_m.8229 | 164 | Succinate dehydrogenase [ubiquinone] flavoprotein subunit 1, mitochondrial | 3 |
| 10414_0.path0_m.609 | 137 | Succinate dehydrogenase subunit 5, mitochondrial | 1 |
| 10414_0.path0_m.609 | 120 | Succinate dehydrogenase subunit 5, mitochondrial | 1 |
| i0_HQ_c174165_f5p1_410_m.84 | 62 | Succinate dehydrogenase subunit 7B, mitochondrial | 3 |
| i1_HQ_c32623_f2p0_1256_m.6619 | 56 | Succinate--CoA ligase [ADP-forming] subunit alpha-2, mitochondrial | 2 |
| i1_HQ_c32623_f2p0_1256_m.6619 | 102 | Succinate--CoA ligase [ADP-forming] subunit alpha-2, mitochondrial | 2 |
| i1_HQ_c32623_f2p0_1256_m.6619 | 80 | Succinate--CoA ligase [ADP-forming] subunit alpha-2, mitochondrial | 2 |
| i1_HQ_c32623_f2p0_1256_m.6619 | 146 | Succinate--CoA ligase [ADP-forming] subunit alpha-2, mitochondrial | 1 |
| i1_HQ_c32623_f2p0_1256_m.6619 | 328 | Succinate--CoA ligase [ADP-forming] subunit alpha-2, mitochondrial | 2 |
| 6655_0.path0_m.2208 | 77 | Succinate--CoA ligase [ADP-forming] subunit alpha-2, mitochondrial | 3 |
| i1_HQ_c32623_f2p0_1256_m.6619 | 79 | Succinate--CoA ligase [ADP-forming] subunit alpha-2, mitochondrial | 3 |
| 5856_0.path0_m.1092 | 377 | Succinate--CoA ligase [ADP-forming] subunit beta, mitochondrial | 1 |
| 5856_0.path0_m.1092 | 205 | Succinate--CoA ligase [ADP-forming] subunit beta, mitochondrial | 3 |
| 5856_0.path0_m.1092 | 100 | Succinate--CoA ligase [ADP-forming] subunit beta, mitochondrial | 2 |
| 5856_0.path0_m.1092 | 119 | Succinate--CoA ligase [ADP-forming] subunit beta, mitochondrial | 3 |
| 5856_0.path0_m.1092 | 174 | Succinate--CoA ligase [ADP-forming] subunit beta, mitochondrial | 3 |
| 5856_0.path0_m.1092 | 163 | Succinate--CoA ligase [ADP-forming] subunit beta, mitochondrial | 3 |
| 5856_0.path0_m.1092 | 89 | Succinate--CoA ligase [ADP-forming] subunit beta, mitochondrial | 3 |
| i0_LQ_c3565_f1p2_807_m.6245 | 22 | Superoxide dismutase [Mn], mitochondrial | 3 |
| i0_LQ_c3565_f1p2_807_m.6245 | 14 | Superoxide dismutase [Mn], mitochondrial | 3 |
| i0_LQ_c3565_f1p2_807_m.6245 | 145 | Superoxide dismutase [Mn], mitochondrial | 3 |
| i0_LQ_c3565_f1p2_807_m.6245 | 28 | Superoxide dismutase [Mn], mitochondrial | 1 |
| i0_LQ_c3565_f1p2_807_m.6245 | 149 | Superoxide dismutase [Mn], mitochondrial | 3 |
| i1_LQ_c23261_f1p0_1733_m.2209 | 69 | Syntaxin-81 | 1 |
| i1_LQ_c23261_f1p0_1733_m.2209 | 67 | Syntaxin-81 | 1 |
| 7915_0.path1_m.3836 | 156 | Thaumatin-like protein | 3 |
| i0_LQ_c306413_f2p7_621_m.6251 | 162 | Thaumatin-like protein | 3 |
| 10388_0.path2_m.564 | 138 | Thioredoxin M4, chloroplastic | 1 |
| i0_HQ_c21421_f3p1_892_m.5310 | 97 | Thioredoxin O1, mitochondrial | 2 |
| 9227_0.path0_m.5457 | 302 | Thylakoid lumenal 29 kDa protein, chloroplastic | 2 |
| 9227_0.path0_m.5457 | 143 | Thylakoid lumenal 29 kDa protein, chloroplastic | 2 |
| 9655_0.path0_m.6073 | 614 | Transketolase, chloroplastic | 2 |
| 9655_0.path0_m.6073 | 596 | Transketolase, chloroplastic | 1 |
| 9655_0.path0_m.6073 | 490 | Transketolase, chloroplastic | 2 |
| 9655_0.path0_m.6073 | 479 | Transketolase, chloroplastic | 1 |
| 8366_0.path0_m.4366 | 96 | Translationally-controlled tumor protein homolog | 2 |
| 8366_0.path0_m.4366 | 85 | Translationally-controlled tumor protein homolog | 1 |
| 8366_0.path0_m.4366 | 92 | Translationally-controlled tumor protein homolog | 2 |
| 8366_0.path0_m.4366 | 119 | Translationally-controlled tumor protein homolog | 3 |
| 465_0.path4_m.7756 | 260 | Transposon Ty3-G Gag-Pol polyprotein | 1 |
| 465_0.path4_m.7756 | 186 | Transposon Ty3-G Gag-Pol polyprotein | 2 |
| 9124_0.path0_m.5309 | 288 | Triosephosphate isomerase, chloroplastic | 2 |
| 10465_0.path0_m.679 | 149 | Triosephosphate isomerase, cytosolic | 1 |
| 9441_0.path0_m.5748 | 18 | Tryptophan aminotransferase-related protein 4 | 3 |
| 9441_0.path0_m.5748 | 366 | Tryptophan aminotransferase-related protein 4 | 3 |
| 10098_0.path0_m.164 | 398 | Tryptophan aminotransferase-related protein 4 | 3 |
| 9441_0.path0_m.5748 | 23 | Tryptophan aminotransferase-related protein 4 | 3 |
| 9441_0.path0_m.5748 | 335 | Tryptophan aminotransferase-related protein 4 | 3 |
| 9441_0.path0_m.5748 | 122 | Tryptophan aminotransferase-related protein 4 | 3 |
| 9441_0.path0_m.5748 | 383 | Tryptophan aminotransferase-related protein 4 | 3 |
| 9441_0.path0_m.5748 | 274 | Tryptophan aminotransferase-related protein 4 | 3 |
| 10098_0.path0_m.164 | 60 | Tryptophan aminotransferase-related protein 4 | 1 |
| 9441_0.path0_m.5748 | 304 | Tryptophan aminotransferase-related protein 4 | 3 |
| 10098_0.path0_m.164 | 52 | Tryptophan aminotransferase-related protein 4 | 2 |
| 9441_0.path0_m.5748 | 272 | Tryptophan aminotransferase-related protein 4 | 3 |
| 10098_0.path0_m.164 | 335 | Tryptophan aminotransferase-related protein 4 | 3 |
| 9049_0.path0_m.5211 | 29 | Ubiquitin-40S ribosomal protein S27a | 1 |
| 9049_0.path0_m.5211 | 81 | Ubiquitin-40S ribosomal protein S27a | 1 |
| 9049_0.path0_m.5211 | 24 | Ubiquitin-40S ribosomal protein S27a | 1 |
| 9049_0.path0_m.5211 | 66 | Ubiquitin-40S ribosomal protein S27a | 1 |
| i0_HQ_c303659_f14p5_668_m.7406 | 63 | Ubiquitin-60S ribosomal protein L40 | 1 |
| i0_HQ_c303659_f14p5_668_m.7406 | 11 | Ubiquitin-60S ribosomal protein L40 | 1 |
| i0_HQ_c303659_f14p5_668_m.7406 | 48 | Ubiquitin-60S ribosomal protein L40 | 1 |
| i0_HQ_c303659_f14p5_668_m.7406 | 6 | Ubiquitin-60S ribosomal protein L40 | 1 |
| i0_LQ_c66978_f2p0_737_m.3 | 47 | Ubiquitin-NEDD8-like protein RUB1 | 1 |
| i0_LQ_c66978_f2p0_737_m.3 | 89 | Ubiquitin-NEDD8-like protein RUB1 | 1 |
| 9412_0.path0_m.5709 | 21 | Uncharacterized mitochondrial protein AtMg00310 | 1 |
| i1_LQ_c14179_f1p0_1554_m.5223 | 34 | Uncharacterized protein At2g27730, mitochondrial | 3 |
| i1_LQ_c32654_f1p0_1326_m.4263 | 207 | Uncharacterized protein At2g37660, chloroplastic | 3 |
| i0_LQ_c249820_f1p1_595_m.1201 | 99 | Uncharacterized protein At2g37660, chloroplastic | 3 |
| i1_LQ_c32654_f1p0_1326_m.4263 | 290 | Uncharacterized protein At2g37660, chloroplastic | 3 |
| 9676_0.path0_m.6093 | 242 | Uncharacterized protein At2g39795, mitochondrial | 3 |
| 9676_0.path0_m.6093 | 234 | Uncharacterized protein At2g39795, mitochondrial | 3 |
| 5593_0.path2_m.936 | 142 | Uncharacterized protein At4g06744 | 1 |
| i0_LQ_c345813_f1p6_439_m.2377 | 28 | Universal stress protein A-like protein | 2 |
| 6596_0.path0_m.2115 | 284 | UTP--glucose-1-phosphate uridylyltransferase | 2 |
| 6596_0.path0_m.2115 | 312 | UTP--glucose-1-phosphate uridylyltransferase | 2 |
| 9002_0.path1_m.5334 | 75 | Vestitone reductase | 1 |
| 2708_0.path6_m.5056 | 575 | V-type proton ATPase catalytic subunit A | 3 |
| 4269_0.path0_m.7401 | 475 | V-type proton ATPase subunit B2 | 2 |
| 4269_0.path0_m.7401 | 40 | V-type proton ATPase subunit B2 | 2 |
| 4269_0.path0_m.7401 | 438 | V-type proton ATPase subunit B2 | 3 |
| 9597_0.path0_m.5983 | 168 | V-type proton ATPase subunit E | 2 |
| 9597_0.path0_m.5983 | 153 | V-type proton ATPase subunit E | 2 |
| 8559_0.path0_m.4694 | 87 | V-type proton ATPase subunit G 1 | 1 |
| 7896_0.path0_m.3792 | 192 | Unknown | 2 |
| 4754_0.path1_m.7943 | 104 | Unknown | 2 |
| 4729_2.path0_m.7895 | 83 | Unknown | 2 |
| 10340_0.path0_m.492 | 8 | Unknown | 3 |
| 4729_2.path0_m.7895 | 103 | Unknown | 1 |
| 790_0.path6_m.3798 | 805 | Unknown | 1 |
| i2_LQ_c39982_f1p0_2058_m.1593 | 6 | Unknown | 2 |
| 5587_0.path0_m.926 | 608 | Unknown | 1 |
| 7813_0.path2_m.3678 | 88 | Unknown | 2 |
| 5587_0.path0_m.926 | 599 | Unknown | 1 |
| 10199_0.path0_m.289 | 54 | Unknown | 1 |
| 7292_0.path0_m.3186 | 23 | Unknown | 1 |
| 4729_2.path0_m.7895 | 93 | Unknown | 1 |

**Table S2. The identified succinylated sites and protein in patchouli plant and in other horticultural species.**

| Species | No. of succinylated sites | No. of succinylated proteins | Reference |
| --- | --- | --- | --- |
| patchouli plant | 466 | 241 | This study |
| Strawberry | 200 | 116 | Fang *et al.* |
| Tomato | 347 | 202 | Jin *et al.* |
| Tea | 3530 | 2132 | Xu *et al.* |
| *Taxus* | 325 | 193 | Shen *et al.* |
| *Dendrobium officinale* | 314 | 207 | Feng *et al.* |

**Table S3** The detailed information of the significantly enriched KEGG pathways.

| **KEGG pathway** | **Protein accession** | **Ksuc Position** | **Protein description** |
| --- | --- | --- | --- |
| sind00950 Isoquinoline alkaloid biosynthesis | 5532_0.path0_m.831 | 22 | Polyphenol oxidase I, chloroplastic |
| sind00950 Isoquinoline alkaloid biosynthesis | 4933_0.path0_m.8308 | 249 | Polyphenol oxidase II, chloroplastic |
| sind00950 Isoquinoline alkaloid biosynthesis | 4933_0.path0_m.8308 | 262 | Polyphenol oxidase II, chloroplastic |
| sind00950 Isoquinoline alkaloid biosynthesis | 4933_0.path0_m.8308 | 460 | Polyphenol oxidase II, chloroplastic |
| sind00950 Isoquinoline alkaloid biosynthesis | 4933_0.path0_m.8308 | 137 | Polyphenol oxidase II, chloroplastic |
| sind00950 Isoquinoline alkaloid biosynthesis | 4933_0.path0_m.8308 | 447 | Polyphenol oxidase II, chloroplastic |
| sind00950 Isoquinoline alkaloid biosynthesis | 4933_0.path0_m.8308 | 427 | Polyphenol oxidase II, chloroplastic |
| sind00950 Isoquinoline alkaloid biosynthesis | 4933_0.path0_m.8308 | 453 | Polyphenol oxidase II, chloroplastic |
| sind00950 Isoquinoline alkaloid biosynthesis | 4933_0.path0_m.8308 | 145 | Polyphenol oxidase II, chloroplastic |
| sind00950 Isoquinoline alkaloid biosynthesis | 4933_0.path0_m.8308 | 419 | Polyphenol oxidase II, chloroplastic |
| sind00950 Isoquinoline alkaloid biosynthesis | 4933_0.path0_m.8308 | 151 | Polyphenol oxidase II, chloroplastic |
| sind00950 Isoquinoline alkaloid biosynthesis | 4202_0.path1_m.7266 | 309 | Polyphenol oxidase II, chloroplastic |
| sind00950 Isoquinoline alkaloid biosynthesis | 4202_0.path1_m.7266 | 528 | Polyphenol oxidase II, chloroplastic |
| sind00950 Isoquinoline alkaloid biosynthesis | 4202_0.path1_m.7266 | 583 | Polyphenol oxidase II, chloroplastic |
| sind00950 Isoquinoline alkaloid biosynthesis | 4202_0.path1_m.7266 | 442 | Polyphenol oxidase II, chloroplastic |
| sind00950 Isoquinoline alkaloid biosynthesis | 4202_0.path1_m.7266 | 140 | Polyphenol oxidase II, chloroplastic |
| sind00950 Isoquinoline alkaloid biosynthesis | 4202_0.path1_m.7266 | 576 | Polyphenol oxidase II, chloroplastic |
| sind00950 Isoquinoline alkaloid biosynthesis | i1_HQ_c14606_f2p0_1675_m.3241 | 179 | Aspartate aminotransferase, mitochondrial |
| sind00950 Isoquinoline alkaloid biosynthesis | i1_HQ_c14606_f2p0_1675_m.3241 | 374 | Aspartate aminotransferase, mitochondrial |
| sind00950 Isoquinoline alkaloid biosynthesis | 5737_0.path0_m.1203 | 149 | Polyphenol oxidase I, chloroplastic |
| sind00950 Isoquinoline alkaloid biosynthesis | 5737_0.path0_m.1203 | 572 | Polyphenol oxidase I, chloroplastic |
| sind00950 Isoquinoline alkaloid biosynthesis | 5737_0.path0_m.1203 | 562 | Polyphenol oxidase I, chloroplastic |
| sind00950 Isoquinoline alkaloid biosynthesis | 5737_0.path0_m.1203 | 524 | Polyphenol oxidase I, chloroplastic |
| sind00950 Isoquinoline alkaloid biosynthesis | 5737_0.path0_m.1203 | 134 | Polyphenol oxidase I, chloroplastic |
| sind00950 Isoquinoline alkaloid biosynthesis | 5737_0.path0_m.1203 | 484 | Polyphenol oxidase I, chloroplastic |
| sind00950 Isoquinoline alkaloid biosynthesis | 5737_0.path0_m.1203 | 305 | Polyphenol oxidase I, chloroplastic |
| sind00950 Isoquinoline alkaloid biosynthesis | 5737_0.path0_m.1203 | 511 | Polyphenol oxidase I, chloroplastic |
| sind00950 Isoquinoline alkaloid biosynthesis | 5737_0.path0_m.1203 | 491 | Polyphenol oxidase I, chloroplastic |
| sind00950 Isoquinoline alkaloid biosynthesis | 5737_0.path0_m.1203 | 126 | Polyphenol oxidase I, chloroplastic |
| sind00053 Ascorbate and aldarate metabolism | 7028_0.path1_m.2813 | 265 | Glutathione S-transferase DHAR3, chloroplastic |
| sind00053 Ascorbate and aldarate metabolism | 8750_0.path0_m.4965 | 267 | L-ascorbate peroxidase 3, peroxisomal |
| sind00053 Ascorbate and aldarate metabolism | 8051_0.path0_m.4044 | 30 | Monodehydroascorbate reductase |
| sind00053 Ascorbate and aldarate metabolism | 5772_0.path0_m.1247 | 365 | L-ascorbate peroxidase T, chloroplastic |
| sind00053 Ascorbate and aldarate metabolism | 5772_0.path0_m.1247 | 345 | L-ascorbate peroxidase T, chloroplastic |
| sind00053 Ascorbate and aldarate metabolism | 5772_0.path0_m.1247 | 103 | L-ascorbate peroxidase T, chloroplastic |
| sind00053 Ascorbate and aldarate metabolism | 5772_0.path0_m.1247 | 144 | L-ascorbate peroxidase T, chloroplastic |
| sind00053 Ascorbate and aldarate metabolism | 9658_0.path1_m.6076 | 85 | Monodehydroascorbate reductase |
| sind00350 Tyrosine metabolism | 5532_0.path0_m.831 | 22 | Polyphenol oxidase I, chloroplastic |
| sind00350 Tyrosine metabolism | 4933_0.path0_m.8308 | 249 | Polyphenol oxidase II, chloroplastic |
| sind00350 Tyrosine metabolism | 4933_0.path0_m.8308 | 262 | Polyphenol oxidase II, chloroplastic |
| sind00350 Tyrosine metabolism | 4933_0.path0_m.8308 | 460 | Polyphenol oxidase II, chloroplastic |
| sind00350 Tyrosine metabolism | 4933_0.path0_m.8308 | 137 | Polyphenol oxidase II, chloroplastic |
| sind00350 Tyrosine metabolism | 4933_0.path0_m.8308 | 447 | Polyphenol oxidase II, chloroplastic |
| sind00350 Tyrosine metabolism | 4933_0.path0_m.8308 | 427 | Polyphenol oxidase II, chloroplastic |
| sind00350 Tyrosine metabolism | 4933_0.path0_m.8308 | 453 | Polyphenol oxidase II, chloroplastic |
| sind00350 Tyrosine metabolism | 4933_0.path0_m.8308 | 145 | Polyphenol oxidase II, chloroplastic |
| sind00350 Tyrosine metabolism | 4933_0.path0_m.8308 | 419 | Polyphenol oxidase II, chloroplastic |
| sind00350 Tyrosine metabolism | 4933_0.path0_m.8308 | 151 | Polyphenol oxidase II, chloroplastic |
| sind00350 Tyrosine metabolism | 4202_0.path1_m.7266 | 309 | Polyphenol oxidase II, chloroplastic |
| sind00350 Tyrosine metabolism | 4202_0.path1_m.7266 | 528 | Polyphenol oxidase II, chloroplastic |
| sind00350 Tyrosine metabolism | 4202_0.path1_m.7266 | 583 | Polyphenol oxidase II, chloroplastic |
| sind00350 Tyrosine metabolism | 4202_0.path1_m.7266 | 442 | Polyphenol oxidase II, chloroplastic |
| sind00350 Tyrosine metabolism | 4202_0.path1_m.7266 | 140 | Polyphenol oxidase II, chloroplastic |
| sind00350 Tyrosine metabolism | 4202_0.path1_m.7266 | 576 | Polyphenol oxidase II, chloroplastic |
| sind00350 Tyrosine metabolism | i1_HQ_c14606_f2p0_1675_m.3241 | 179 | Aspartate aminotransferase, mitochondrial |
| sind00350 Tyrosine metabolism | i1_HQ_c14606_f2p0_1675_m.3241 | 374 | Aspartate aminotransferase, mitochondrial |
| sind00350 Tyrosine metabolism | i1_LQ_c36180_f1p0_1059_m.2215 | 172 | Acylpyruvase FAHD1, mitochondrial |
| sind00350 Tyrosine metabolism | 5737_0.path0_m.1203 | 149 | Polyphenol oxidase I, chloroplastic |
| sind00350 Tyrosine metabolism | 5737_0.path0_m.1203 | 572 | Polyphenol oxidase I, chloroplastic |
| sind00350 Tyrosine metabolism | 5737_0.path0_m.1203 | 562 | Polyphenol oxidase I, chloroplastic |
| sind00350 Tyrosine metabolism | 5737_0.path0_m.1203 | 524 | Polyphenol oxidase I, chloroplastic |
| sind00350 Tyrosine metabolism | 5737_0.path0_m.1203 | 134 | Polyphenol oxidase I, chloroplastic |
| sind00350 Tyrosine metabolism | 5737_0.path0_m.1203 | 484 | Polyphenol oxidase I, chloroplastic |
| sind00350 Tyrosine metabolism | 5737_0.path0_m.1203 | 305 | Polyphenol oxidase I, chloroplastic |
| sind00350 Tyrosine metabolism | 5737_0.path0_m.1203 | 511 | Polyphenol oxidase I, chloroplastic |
| sind00350 Tyrosine metabolism | 5737_0.path0_m.1203 | 491 | Polyphenol oxidase I, chloroplastic |
| sind00350 Tyrosine metabolism | 5737_0.path0_m.1203 | 126 | Polyphenol oxidase I, chloroplastic |
| sind01100 Metabolic pathways | i1_HQ_c14606_f2p0_1675_m.3241 | 179 | Aspartate aminotransferase, mitochondrial |
| sind01100 Metabolic pathways | i1_HQ_c14606_f2p0_1675_m.3241 | 374 | Aspartate aminotransferase, mitochondrial |
| sind01100 Metabolic pathways | 1931_0.path2_m.3930 | 373 | 2-oxoglutarate dehydrogenase, mitochondrial |
| sind01100 Metabolic pathways | 1931_0.path2_m.3930 | 437 | 2-oxoglutarate dehydrogenase, mitochondrial |
| sind01100 Metabolic pathways | 1931_0.path2_m.3930 | 446 | 2-oxoglutarate dehydrogenase, mitochondrial |
| sind01100 Metabolic pathways | 1931_0.path2_m.3930 | 218 | 2-oxoglutarate dehydrogenase, mitochondrial |
| sind01100 Metabolic pathways | 1931_0.path2_m.3930 | 392 | 2-oxoglutarate dehydrogenase, mitochondrial |
| sind01100 Metabolic pathways | 1931_0.path2_m.3930 | 362 | 2-oxoglutarate dehydrogenase, mitochondrial |
| sind01100 Metabolic pathways | 1931_0.path2_m.3930 | 459 | 2-oxoglutarate dehydrogenase, mitochondrial |
| sind01100 Metabolic pathways | 1931_0.path2_m.3930 | 843 | 2-oxoglutarate dehydrogenase, mitochondrial |
| sind01100 Metabolic pathways | 11907_0.path0_m.2413 | 311 | Glutamine synthetase cytosolic isozyme |
| sind01100 Metabolic pathways | i1_LQ_c7379_f1p0_1442_m.2478 | 292 | 1-aminocyclopropane-1-carboxylate oxidase 3 |
| sind01100 Metabolic pathways | 5737_0.path0_m.1203 | 149 | Polyphenol oxidase I, chloroplastic |
| sind01100 Metabolic pathways | 5737_0.path0_m.1203 | 572 | Polyphenol oxidase I, chloroplastic |
| sind01100 Metabolic pathways | 5737_0.path0_m.1203 | 562 | Polyphenol oxidase I, chloroplastic |
| sind01100 Metabolic pathways | 5737_0.path0_m.1203 | 524 | Polyphenol oxidase I, chloroplastic |
| sind01100 Metabolic pathways | 5737_0.path0_m.1203 | 134 | Polyphenol oxidase I, chloroplastic |
| sind01100 Metabolic pathways | 5737_0.path0_m.1203 | 484 | Polyphenol oxidase I, chloroplastic |
| sind01100 Metabolic pathways | 5737_0.path0_m.1203 | 305 | Polyphenol oxidase I, chloroplastic |
| sind01100 Metabolic pathways | 5737_0.path0_m.1203 | 511 | Polyphenol oxidase I, chloroplastic |
| sind01100 Metabolic pathways | 5737_0.path0_m.1203 | 491 | Polyphenol oxidase I, chloroplastic |
| sind01100 Metabolic pathways | 5737_0.path0_m.1203 | 126 | Polyphenol oxidase I, chloroplastic |
| sind01100 Metabolic pathways | 5532_0.path0_m.831 | 22 | Polyphenol oxidase I, chloroplastic |
| sind01100 Metabolic pathways | 2221_0.path0_m.4534 | 604 | 2-oxoglutarate dehydrogenase, mitochondrial |
| sind01100 Metabolic pathways | 4133_0.path3_m.7108 | 70 | ATP synthase subunit beta, mitochondrial |
| sind01100 Metabolic pathways | 4133_0.path3_m.7108 | 78 | ATP synthase subunit beta, mitochondrial |
| sind01100 Metabolic pathways | i0_LQ_c35465_f1p1_640_m.6645 | 115 | Aminomethyltransferase, mitochondrial |
| sind01100 Metabolic pathways | i1_LQ_c36180_f1p0_1059_m.2215 | 172 | Acylpyruvase FAHD1, mitochondrial |
| sind01100 Metabolic pathways | 7090_0.path1_m.2911 | 178 | Nucleoside diphosphate kinase IV, chloroplastic_mitochondrial |
| sind01100 Metabolic pathways | 6115_0.path0_m.1574 | 266 | Beta-glucosidase 12 |
| sind01100 Metabolic pathways | 6115_0.path0_m.1574 | 113 | Beta-glucosidase 12 |
| sind01100 Metabolic pathways | 6706_0.path0_m.2288 | 180 | Cytochrome f |
| sind01100 Metabolic pathways | 6706_0.path0_m.2288 | 174 | Cytochrome f |
| sind01100 Metabolic pathways | 6706_0.path0_m.2288 | 216 | Cytochrome f |
| sind01100 Metabolic pathways | 5136_0.path0_m.111 | 308 | Photosystem II CP47 reaction center protein |
| sind01100 Metabolic pathways | 5136_0.path0_m.111 | 304 | Photosystem II CP47 reaction center protein |
| sind01100 Metabolic pathways | 5136_0.path0_m.111 | 438 | Photosystem II CP47 reaction center protein |
| sind01100 Metabolic pathways | 8418_0.path0_m.4439 | 115 | Peroxidase 4 |
| sind01100 Metabolic pathways | 8051_0.path0_m.4044 | 30 | Monodehydroascorbate reductase |
| sind01100 Metabolic pathways | 5250_0.path0_m.317 | 709 | Glyoxysomal fatty acid beta-oxidation multifunctional protein MFP-a |
| sind01100 Metabolic pathways | 5250_0.path0_m.317 | 374 | Glyoxysomal fatty acid beta-oxidation multifunctional protein MFP-a |
| sind01100 Metabolic pathways | 5250_0.path0_m.317 | 355 | Glyoxysomal fatty acid beta-oxidation multifunctional protein MFP-a |
| sind01100 Metabolic pathways | 10112_0.path0_m.184 | 169 | ATP synthase subunit O, mitochondrial |
| sind01100 Metabolic pathways | 8352_0.path0_m.4346 | 113 | Chlorophyll a-b binding protein CP26, chloroplastic |
| sind01100 Metabolic pathways | i1_LQ_c26180_f2p0_1342_m.6236 | 203 | Dihydrolipoyllysine-residue acetyltransferase component 1 of pyruvate dehydrogenase complex, mitochondrial |
| sind01100 Metabolic pathways | 7028_0.path1_m.2813 | 265 | Glutathione S-transferase DHAR3, chloroplastic |
| sind01100 Metabolic pathways | 4658_0.path0_m.7768 | 148 | Phosphoglycerate kinase, chloroplastic |
| sind01100 Metabolic pathways | 4897_0.path0_m.8229 | 164 | Succinate dehydrogenase [ubiquinone] flavoprotein subunit 1, mitochondrial |
| sind01100 Metabolic pathways | 4897_0.path0_m.8229 | 318 | Succinate dehydrogenase [ubiquinone] flavoprotein subunit 1, mitochondrial |
| sind01100 Metabolic pathways | i0_LQ_c104330_f1p1_837_m.4398 | 221 | Peroxisomal (S)-2-hydroxy-acid oxidase GLO2 |
| sind01100 Metabolic pathways | 8394_0.path0_m.4400 | 119 | Glyceraldehyde-3-phosphate dehydrogenase B, chloroplastic |
| sind01100 Metabolic pathways | 8394_0.path0_m.4400 | 271 | Glyceraldehyde-3-phosphate dehydrogenase B, chloroplastic |
| sind01100 Metabolic pathways | 8394_0.path0_m.4400 | 91 | Glyceraldehyde-3-phosphate dehydrogenase B, chloroplastic |
| sind01100 Metabolic pathways | 8472_0.path0_m.4524 | 137 | Photosystem I reaction center subunit VI, chloroplastic |
| sind01100 Metabolic pathways | 8472_0.path0_m.4524 | 98 | Photosystem I reaction center subunit VI, chloroplastic |
| sind01100 Metabolic pathways | 4752_1.path0_m.7937 | 124 | Dihydrolipoyllysine-residue succinyltransferase component of 2-oxoglutarate dehydrogenase complex 2, mitochondrial |
| sind01100 Metabolic pathways | 4752_1.path0_m.7937 | 322 | Dihydrolipoyllysine-residue succinyltransferase component of 2-oxoglutarate dehydrogenase complex 2, mitochondrial |
| sind01100 Metabolic pathways | 4752_1.path0_m.7937 | 249 | Dihydrolipoyllysine-residue succinyltransferase component of 2-oxoglutarate dehydrogenase complex 2, mitochondrial |
| sind01100 Metabolic pathways | 11384_0.path0_m.1588 | 308 | Sedoheptulose-1,7-bisphosphatase, chloroplastic |
| sind01100 Metabolic pathways | 7383_0.path0_m.3334 | 53 | Chlorophyll a-b binding protein CP24 10A, chloroplastic |
| sind01100 Metabolic pathways | 1242_0.path10_m.2975 | 613 | 2-oxoglutarate dehydrogenase, mitochondrial |
| sind01100 Metabolic pathways | 1242_0.path10_m.2975 | 603 | 2-oxoglutarate dehydrogenase, mitochondrial |
| sind01100 Metabolic pathways | 8582_0.path0_m.4729 | 99 | Acidic endochitinase |
| sind01100 Metabolic pathways | 8582_0.path0_m.4729 | 181 | Acidic endochitinase |
| sind01100 Metabolic pathways | 8582_0.path0_m.4729 | 275 | Acidic endochitinase |
| sind01100 Metabolic pathways | 8582_0.path0_m.4729 | 288 | Acidic endochitinase |
| sind01100 Metabolic pathways | 8582_0.path0_m.4729 | 113 | Acidic endochitinase |
| sind01100 Metabolic pathways | 8582_0.path0_m.4729 | 110 | Acidic endochitinase |
| sind01100 Metabolic pathways | 4175_0.path0_m.7181 | 384 | ATP synthase subunit alpha, mitochondrial |
| sind01100 Metabolic pathways | 4175_0.path0_m.7181 | 189 | ATP synthase subunit alpha, mitochondrial |
| sind01100 Metabolic pathways | 4175_0.path0_m.7181 | 476 | ATP synthase subunit alpha, mitochondrial |
| sind01100 Metabolic pathways | 4175_0.path0_m.7181 | 387 | ATP synthase subunit alpha, mitochondrial |
| sind01100 Metabolic pathways | 4175_0.path0_m.7181 | 142 | ATP synthase subunit alpha, mitochondrial |
| sind01100 Metabolic pathways | 10683_0.path0_m.953 | 261 | Formate dehydrogenase, mitochondrial |
| sind01100 Metabolic pathways | 3549_0.path0_m.6407 | 251 | Leucine aminopeptidase 2, chloroplastic |
| sind01100 Metabolic pathways | 4700_0.path0_m.7851 | 97 | NADH dehydrogenase [ubiquinone] flavoprotein 1, mitochondrial |
| sind01100 Metabolic pathways | i1_LQ_c77746_f1p0_1530_m.131 | 223 | Probable acetyl-CoA acetyltransferase, cytosolic 2 |
| sind01100 Metabolic pathways | 10612_0.path0_m.864 | 64 | Probable enoyl-CoA hydratase 2, mitochondrial |
| sind01100 Metabolic pathways | 10612_0.path0_m.864 | 111 | Probable enoyl-CoA hydratase 2, mitochondrial |
| sind01100 Metabolic pathways | 2769_0.path0_m.5164 | 466 | ATP synthase subunit alpha, chloroplastic |
| sind01100 Metabolic pathways | 2769_0.path0_m.5164 | 114 | ATP synthase subunit alpha, chloroplastic |
| sind01100 Metabolic pathways | 3879_0.path0_m.6640 | 99 | NAD-dependent malic enzyme 62 kDa isoform, mitochondrial |
| sind01100 Metabolic pathways | 3879_0.path0_m.6640 | 299 | NAD-dependent malic enzyme 62 kDa isoform, mitochondrial |
| sind01100 Metabolic pathways | 4269_0.path0_m.7401 | 438 | V-type proton ATPase subunit B2 |
| sind01100 Metabolic pathways | 7598_0.path21_m.3382 | 41 | Chlorophyll a-b binding protein of LHCII type I, chloroplastic (Fragment) |
| sind01100 Metabolic pathways | 3962_0.path0_m.6781 | 92 | ATP synthase subunit beta, mitochondrial |
| sind01100 Metabolic pathways | 3962_0.path0_m.6781 | 100 | ATP synthase subunit beta, mitochondrial |
| sind01100 Metabolic pathways | 9538_0.path0_m.5901 | 354 | Glutamine synthetase, chloroplastic |
| sind01100 Metabolic pathways | 9538_0.path0_m.5901 | 101 | Glutamine synthetase, chloroplastic |
| sind01100 Metabolic pathways | 9538_0.path0_m.5901 | 168 | Glutamine synthetase, chloroplastic |
| sind01100 Metabolic pathways | i1_LQ_c67740_f1p0_1601_m.6729 | 74 | Phosphoglycerate kinase, cytosolic |
| sind01100 Metabolic pathways | 11884_0.path0_m.2391 | 40 | Fructose-bisphosphate aldolase 6, cytosolic |
| sind01100 Metabolic pathways | 11884_0.path0_m.2391 | 357 | Fructose-bisphosphate aldolase 6, cytosolic |
| sind01100 Metabolic pathways | 5856_0.path0_m.1092 | 163 | Succinate--CoA ligase [ADP-forming] subunit beta, mitochondrial |
| sind01100 Metabolic pathways | 5856_0.path0_m.1092 | 119 | Succinate--CoA ligase [ADP-forming] subunit beta, mitochondrial |
| sind01100 Metabolic pathways | 5856_0.path0_m.1092 | 205 | Succinate--CoA ligase [ADP-forming] subunit beta, mitochondrial |
| sind01100 Metabolic pathways | 5856_0.path0_m.1092 | 174 | Succinate--CoA ligase [ADP-forming] subunit beta, mitochondrial |
| sind01100 Metabolic pathways | 5856_0.path0_m.1092 | 89 | Succinate--CoA ligase [ADP-forming] subunit beta, mitochondrial |
| sind01100 Metabolic pathways | 9127_0.path0_m.5312 | 99 | Photosystem I reaction center subunit IV, chloroplastic |
| sind01100 Metabolic pathways | 7809_0.path0_m.3670 | 147 | Cytochrome b6-f complex iron-sulfur subunit, chloroplastic |
| sind01100 Metabolic pathways | 7809_0.path0_m.3670 | 133 | Cytochrome b6-f complex iron-sulfur subunit, chloroplastic |
| sind01100 Metabolic pathways | 5201_0.path1_m.207 | 386 | Raucaffricine-O-beta-D-glucosidase |
| sind01100 Metabolic pathways | 2789_0.path4_m.5221 | 83 | Aconitate hydratase 2, mitochondrial |
| sind01100 Metabolic pathways | i1_HQ_c32623_f2p0_1256_m.6619 | 79 | Succinate--CoA ligase [ADP-forming] subunit alpha-2, mitochondrial |
| sind01100 Metabolic pathways | 9666_0.path0_m.6084 | 157 | Dihydrolipoyllysine-residue acetyltransferase component 3 of pyruvate dehydrogenase complex, mitochondrial |
| sind01100 Metabolic pathways | 9666_0.path0_m.6084 | 150 | Dihydrolipoyllysine-residue acetyltransferase component 3 of pyruvate dehydrogenase complex, mitochondrial |
| sind01100 Metabolic pathways | 8673_0.path2_m.4855 | 11 | Ribulose bisphosphate carboxylase small chain, chloroplastic |
| sind01100 Metabolic pathways | 8673_0.path2_m.4855 | 107 | Ribulose bisphosphate carboxylase small chain, chloroplastic |
| sind01100 Metabolic pathways | 10025_0.path0_m.37 | 7 | Chlorophyll a-b binding protein of LHCII type I, chloroplastic (Fragment) |
| sind01100 Metabolic pathways | 5592_0.path0_m.933 | 306 | Dihydrolipoyl dehydrogenase 1, mitochondrial |
| sind01100 Metabolic pathways | 5592_0.path0_m.933 | 181 | Dihydrolipoyl dehydrogenase 1, mitochondrial |
| sind01100 Metabolic pathways | 5592_0.path0_m.933 | 207 | Dihydrolipoyl dehydrogenase 1, mitochondrial |
| sind01100 Metabolic pathways | 5592_0.path0_m.933 | 97 | Dihydrolipoyl dehydrogenase 1, mitochondrial |
| sind01100 Metabolic pathways | 5592_0.path0_m.933 | 218 | Dihydrolipoyl dehydrogenase 1, mitochondrial |
| sind01100 Metabolic pathways | 5592_0.path0_m.933 | 176 | Dihydrolipoyl dehydrogenase 1, mitochondrial |
| sind01100 Metabolic pathways | 5592_0.path0_m.933 | 448 | Dihydrolipoyl dehydrogenase 1, mitochondrial |
| sind01100 Metabolic pathways | 5592_0.path0_m.933 | 205 | Dihydrolipoyl dehydrogenase 1, mitochondrial |
| sind01100 Metabolic pathways | 5592_0.path0_m.933 | 166 | Dihydrolipoyl dehydrogenase 1, mitochondrial |
| sind01100 Metabolic pathways | 5592_0.path0_m.933 | 173 | Dihydrolipoyl dehydrogenase 1, mitochondrial |
| sind01100 Metabolic pathways | 5592_0.path0_m.933 | 354 | Dihydrolipoyl dehydrogenase 1, mitochondrial |
| sind01100 Metabolic pathways | 5592_0.path0_m.933 | 133 | Dihydrolipoyl dehydrogenase 1, mitochondrial |
| sind01100 Metabolic pathways | 4933_0.path0_m.8308 | 249 | Polyphenol oxidase II, chloroplastic |
| sind01100 Metabolic pathways | 4933_0.path0_m.8308 | 262 | Polyphenol oxidase II, chloroplastic |
| sind01100 Metabolic pathways | 4933_0.path0_m.8308 | 460 | Polyphenol oxidase II, chloroplastic |
| sind01100 Metabolic pathways | 4933_0.path0_m.8308 | 137 | Polyphenol oxidase II, chloroplastic |
| sind01100 Metabolic pathways | 4933_0.path0_m.8308 | 447 | Polyphenol oxidase II, chloroplastic |
| sind01100 Metabolic pathways | 4933_0.path0_m.8308 | 427 | Polyphenol oxidase II, chloroplastic |
| sind01100 Metabolic pathways | 4933_0.path0_m.8308 | 453 | Polyphenol oxidase II, chloroplastic |
| sind01100 Metabolic pathways | 4933_0.path0_m.8308 | 145 | Polyphenol oxidase II, chloroplastic |
| sind01100 Metabolic pathways | 4933_0.path0_m.8308 | 419 | Polyphenol oxidase II, chloroplastic |
| sind01100 Metabolic pathways | 4933_0.path0_m.8308 | 151 | Polyphenol oxidase II, chloroplastic |
| sind01100 Metabolic pathways | i1_LQ_c46534_f1p0_1143_m.1204 | 131 | Probable enoyl-CoA hydratase 2, mitochondrial |
| sind01100 Metabolic pathways | 6655_0.path0_m.2208 | 77 | Succinate--CoA ligase [ADP-forming] subunit alpha-2, mitochondrial |
| sind01100 Metabolic pathways | 4202_0.path1_m.7266 | 309 | Polyphenol oxidase II, chloroplastic |
| sind01100 Metabolic pathways | 4202_0.path1_m.7266 | 528 | Polyphenol oxidase II, chloroplastic |
| sind01100 Metabolic pathways | 4202_0.path1_m.7266 | 583 | Polyphenol oxidase II, chloroplastic |
| sind01100 Metabolic pathways | 4202_0.path1_m.7266 | 442 | Polyphenol oxidase II, chloroplastic |
| sind01100 Metabolic pathways | 4202_0.path1_m.7266 | 140 | Polyphenol oxidase II, chloroplastic |
| sind01100 Metabolic pathways | 4202_0.path1_m.7266 | 576 | Polyphenol oxidase II, chloroplastic |
| sind01100 Metabolic pathways | i2_LQ_c21171_f1p1_2202_m.2177 | 92 | Hevamine-A |
| sind01100 Metabolic pathways | 8934_0.path0_m.5246 | 127 | Chitinase 5 |
| sind01100 Metabolic pathways | 8934_0.path0_m.5246 | 171 | Chitinase 5 |
| sind01100 Metabolic pathways | i1_LQ_c51375_f1p0_1803_m.7249 | 94 | ATP synthase subunit beta, mitochondrial |
| sind01100 Metabolic pathways | i1_LQ_c51375_f1p0_1803_m.7249 | 86 | ATP synthase subunit beta, mitochondrial |
| sind01100 Metabolic pathways | i0_LQ_c59477_f1p0_991_m.4245 | 149 | ATP synthase subunit d, mitochondrial |
| sind01100 Metabolic pathways | i0_LQ_c59477_f1p0_991_m.4245 | 87 | ATP synthase subunit d, mitochondrial |
| sind01100 Metabolic pathways | i0_LQ_c59477_f1p0_991_m.4245 | 7 | ATP synthase subunit d, mitochondrial |
| sind01100 Metabolic pathways | i0_LQ_c59477_f1p0_991_m.4245 | 80 | ATP synthase subunit d, mitochondrial |
| sind01100 Metabolic pathways | i0_LQ_c59477_f1p0_991_m.4245 | 17 | ATP synthase subunit d, mitochondrial |
| sind01100 Metabolic pathways | i0_LQ_c59477_f1p0_991_m.4245 | 100 | ATP synthase subunit d, mitochondrial |
| sind01100 Metabolic pathways | i0_LQ_c59477_f1p0_991_m.4245 | 14 | ATP synthase subunit d, mitochondrial |
| sind01100 Metabolic pathways | 6328_0.path0_m.1926 | 291 | Cytochrome c1 2, heme protein, mitochondrial |
| sind01100 Metabolic pathways | 6817_0.path0_m.2451 | 207 | Adenylate kinase 4 |
| sind01100 Metabolic pathways | 6817_0.path0_m.2451 | 72 | Adenylate kinase 4 |
| sind01100 Metabolic pathways | 5638_0.path0_m.1009 | 190 | Ribulose bisphosphate carboxylase large chain |
| sind01100 Metabolic pathways | 5638_0.path0_m.1009 | 182 | Ribulose bisphosphate carboxylase large chain |
| sind01100 Metabolic pathways | 5638_0.path0_m.1009 | 39 | Ribulose bisphosphate carboxylase large chain |
| sind01100 Metabolic pathways | 5638_0.path0_m.1009 | 341 | Ribulose bisphosphate carboxylase large chain |
| sind01100 Metabolic pathways | 5638_0.path0_m.1009 | 153 | Ribulose bisphosphate carboxylase large chain |
| sind01100 Metabolic pathways | 7914_0.path0_m.3834 | 127 | Photosystem I reaction center subunit III, chloroplastic |
| sind01100 Metabolic pathways | 7914_0.path0_m.3834 | 117 | Photosystem I reaction center subunit III, chloroplastic |
| sind01100 Metabolic pathways | 7914_0.path0_m.3834 | 96 | Photosystem I reaction center subunit III, chloroplastic |
| sind01100 Metabolic pathways | i1_LQ_c50855_f1p1_1111_m.2233 | 50 | Chlorophyllase-1 |
| sind01100 Metabolic pathways | 9658_0.path1_m.6076 | 85 | Monodehydroascorbate reductase |
| sind01100 Metabolic pathways | 9636_0.path0_m.6039 | 76 | Pyruvate dehydrogenase E1 component subunit beta-1, mitochondrial |
| sind01100 Metabolic pathways | 9685_0.path0_m.6107 | 401 | Serine hydroxymethyltransferase, mitochondrial |
| sind01100 Metabolic pathways | 9685_0.path0_m.6107 | 412 | Serine hydroxymethyltransferase, mitochondrial |
| sind01100 Metabolic pathways | 9685_0.path0_m.6107 | 185 | Serine hydroxymethyltransferase, mitochondrial |
| sind01100 Metabolic pathways | 9685_0.path0_m.6107 | 471 | Serine hydroxymethyltransferase, mitochondrial |
| sind01100 Metabolic pathways | 9685_0.path0_m.6107 | 507 | Serine hydroxymethyltransferase, mitochondrial |
| sind01100 Metabolic pathways | 9654_0.path0_m.6072 | 111 | Photosystem I reaction center subunit V, chloroplastic |
| sind01100 Metabolic pathways | 9257_0.path1_m.5500 | 401 | Citrate synthase, mitochondrial |
| sind01100 Metabolic pathways | 9257_0.path1_m.5500 | 346 | Citrate synthase, mitochondrial |
| sind01100 Metabolic pathways | 5891_0.path0_m.1161 | 266 | ATP-citrate synthase beta chain protein 2 |
| sind01100 Metabolic pathways | i0_LQ_c102321_f1p1_510_m.5175 | 51 | Photosystem II 10 kDa polypeptide, chloroplastic |
| sind01100 Metabolic pathways | 9209_0.path0_m.5429 | 228 | Citrate synthase, glyoxysomal |
| sind01100 Metabolic pathways | 3539_0.path0_m.6388 | 867 | Glycine dehydrogenase (decarboxylating), mitochondrial |
| sind01100 Metabolic pathways | 3539_0.path0_m.6388 | 700 | Glycine dehydrogenase (decarboxylating), mitochondrial |
| sind01100 Metabolic pathways | 8146_0.path0_m.4182 | 42 | Chlorophyll a-b binding protein 36, chloroplastic |
| sind01100 Metabolic pathways | 8146_0.path0_m.4182 | 124 | Chlorophyll a-b binding protein 36, chloroplastic |
| sind01100 Metabolic pathways | i0_LQ_c128861_f1p0_857_m.7234 | 155 | Oxygen-evolving enhancer protein 3-1, chloroplastic |
| sind01100 Metabolic pathways | i0_LQ_c128861_f1p0_857_m.7234 | 129 | Oxygen-evolving enhancer protein 3-1, chloroplastic |
| sind01100 Metabolic pathways | i0_LQ_c128861_f1p0_857_m.7234 | 115 | Oxygen-evolving enhancer protein 3-1, chloroplastic |
| sind01100 Metabolic pathways | i0_LQ_c128861_f1p0_857_m.7234 | 166 | Oxygen-evolving enhancer protein 3-1, chloroplastic |
| sind01100 Metabolic pathways | i0_LQ_c128861_f1p0_857_m.7234 | 132 | Oxygen-evolving enhancer protein 3-1, chloroplastic |
| sind01100 Metabolic pathways | i0_LQ_c128861_f1p0_857_m.7234 | 76 | Oxygen-evolving enhancer protein 3-1, chloroplastic |
| sind01100 Metabolic pathways | i0_LQ_c128861_f1p0_857_m.7234 | 117 | Oxygen-evolving enhancer protein 3-1, chloroplastic |
| sind01100 Metabolic pathways | i0_LQ_c128861_f1p0_857_m.7234 | 82 | Oxygen-evolving enhancer protein 3-1, chloroplastic |
| sind01100 Metabolic pathways | 8601_0.path0_m.4749 | 205 | Isocitrate dehydrogenase [NAD] catalytic subunit 5, mitochondrial |
| sind01100 Metabolic pathways | 8601_0.path0_m.4749 | 332 | Isocitrate dehydrogenase [NAD] catalytic subunit 5, mitochondrial |
| sind01100 Metabolic pathways | 8601_0.path0_m.4749 | 168 | Isocitrate dehydrogenase [NAD] catalytic subunit 5, mitochondrial |
| sind01100 Metabolic pathways | i1_LQ_c70037_f1p0_1177_m.2262 | 94 | Cytochrome c oxidase subunit 5b-1, mitochondrial |
| sind01100 Metabolic pathways | 5071_0.path1_m.2 | 68 | Photosystem I P700 chlorophyll a apoprotein A2 |
| sind01100 Metabolic pathways | i1_LQ_c14245_f1p3_1457_m.1128 | 279 | 3-hydroxyisobutyryl-CoA hydrolase-like protein 3, mitochondrial |
| sind01100 Metabolic pathways | 6440_0.path0_m.2112 | 312 | Glutamine synthetase cytosolic isozyme |
| sind01100 Metabolic pathways | 6440_0.path0_m.2112 | 289 | Glutamine synthetase cytosolic isozyme |
| sind01100 Metabolic pathways | 6440_0.path0_m.2112 | 322 | Glutamine synthetase cytosolic isozyme |
| sind01100 Metabolic pathways | 6944_0.path0_m.2670 | 16 | ATP-citrate synthase alpha chain protein 2 |
| sind01100 Metabolic pathways | 6944_0.path0_m.2670 | 58 | ATP-citrate synthase alpha chain protein 2 |
| sind01100 Metabolic pathways | 6598_0.path0_m.2118 | 181 | Oxygen-evolving enhancer protein 1, chloroplastic |
| sind01100 Metabolic pathways | 6598_0.path0_m.2118 | 136 | Oxygen-evolving enhancer protein 1, chloroplastic |
| sind01100 Metabolic pathways | 6598_0.path0_m.2118 | 90 | Oxygen-evolving enhancer protein 1, chloroplastic |
| sind01100 Metabolic pathways | 6598_0.path0_m.2118 | 120 | Oxygen-evolving enhancer protein 1, chloroplastic |
| sind01100 Metabolic pathways | 6598_0.path0_m.2118 | 125 | Oxygen-evolving enhancer protein 1, chloroplastic |
| sind01100 Metabolic pathways | 6598_0.path0_m.2118 | 213 | Oxygen-evolving enhancer protein 1, chloroplastic |
| sind01100 Metabolic pathways | 6598_0.path0_m.2118 | 113 | Oxygen-evolving enhancer protein 1, chloroplastic |
| sind01100 Metabolic pathways | 2708_0.path6_m.5056 | 575 | V-type proton ATPase catalytic subunit A |
| sind01100 Metabolic pathways | 5827_0.path3_m.1058 | 151 | Peroxisomal (S)-2-hydroxy-acid oxidase GLO1 |
| sind01100 Metabolic pathways | 3773_0.path1_m.6408 | 229 | NAD-dependent malic enzyme 59 kDa isoform, mitochondrial |
| sind01100 Metabolic pathways | i1_HQ_c41079_f2p0_1865_m.5127 | 54 | Glutamate--glyoxylate aminotransferase 2 |
| sind01100 Metabolic pathways | 7619_0.path0_m.3408 | 231 | Oxygen-evolving enhancer protein 2-1, chloroplastic |
| sind01100 Metabolic pathways | 7619_0.path0_m.3408 | 112 | Oxygen-evolving enhancer protein 2-1, chloroplastic |
| sind01100 Metabolic pathways | 1589_0.path0_m.3099 | 128 | Aconitate hydratase 2, mitochondrial |
| sind01100 Metabolic pathways | 1589_0.path0_m.3099 | 122 | Aconitate hydratase 2, mitochondrial |
| sind01100 Metabolic pathways | 1589_0.path0_m.3099 | 558 | Aconitate hydratase 2, mitochondrial |
| sind01100 Metabolic pathways | 1589_0.path0_m.3099 | 381 | Aconitate hydratase 2, mitochondrial |
| sind01100 Metabolic pathways | 1589_0.path0_m.3099 | 114 | Aconitate hydratase 2, mitochondrial |
| sind01100 Metabolic pathways | 1589_0.path0_m.3099 | 166 | Aconitate hydratase 2, mitochondrial |
| sind01100 Metabolic pathways | 1589_0.path0_m.3099 | 844 | Aconitate hydratase 2, mitochondrial |
| sind01100 Metabolic pathways | 1589_0.path0_m.3099 | 855 | Aconitate hydratase 2, mitochondrial |
| sind01100 Metabolic pathways | 1589_0.path0_m.3099 | 508 | Aconitate hydratase 2, mitochondrial |
| sind01100 Metabolic pathways | 1589_0.path0_m.3099 | 574 | Aconitate hydratase 2, mitochondrial |
| sind01100 Metabolic pathways | i1_HQ_c60950_f14p0_1160_m.1131 | 220 | Cysteine synthase |
| sind01100 Metabolic pathways | 8810_0.path5_m.5063 | 147 | Photosystem I reaction center subunit II, chloroplastic |
| sind01100 Metabolic pathways | 8810_0.path5_m.5063 | 95 | Photosystem I reaction center subunit II, chloroplastic |
| sind01100 Metabolic pathways | 8810_0.path5_m.5063 | 139 | Photosystem I reaction center subunit II, chloroplastic |
| sind01100 Metabolic pathways | 11093_0.path0_m.1258 | 154 | Glycine cleavage system H protein 3, mitochondrial |
| sind01100 Metabolic pathways | 11093_0.path0_m.1258 | 140 | Glycine cleavage system H protein 3, mitochondrial |
| sind01100 Metabolic pathways | 11093_0.path0_m.1258 | 47 | Glycine cleavage system H protein 3, mitochondrial |
| sind01100 Metabolic pathways | 7112_0.path0_m.2939 | 371 | Pyruvate dehydrogenase E1 component subunit alpha, mitochondrial |
| sind01100 Metabolic pathways | 7112_0.path0_m.2939 | 333 | Pyruvate dehydrogenase E1 component subunit alpha, mitochondrial |
| sind01100 Metabolic pathways | 7112_0.path0_m.2939 | 354 | Pyruvate dehydrogenase E1 component subunit alpha, mitochondrial |
| sind01100 Metabolic pathways | 7112_0.path0_m.2939 | 239 | Pyruvate dehydrogenase E1 component subunit alpha, mitochondrial |
| sind01100 Metabolic pathways | 7112_0.path0_m.2939 | 322 | Pyruvate dehydrogenase E1 component subunit alpha, mitochondrial |
| sind01100 Metabolic pathways | 10152_0.path0_m.229 | 369 | Fructose-bisphosphate aldolase 2, chloroplastic |
| sind01100 Metabolic pathways | 10152_0.path0_m.229 | 61 | Fructose-bisphosphate aldolase 2, chloroplastic |
| sind01100 Metabolic pathways | 4476_0.path1_m.7434 | 411 | NADP-dependent malic enzyme |
| sind00630 Glyoxylate and dicarboxylate metabolism | 9209_0.path0_m.5429 | 228 | Citrate synthase, glyoxysomal |
| sind00630 Glyoxylate and dicarboxylate metabolism | 3539_0.path0_m.6388 | 867 | Glycine dehydrogenase (decarboxylating), mitochondrial |
| sind00630 Glyoxylate and dicarboxylate metabolism | 3539_0.path0_m.6388 | 700 | Glycine dehydrogenase (decarboxylating), mitochondrial |
| sind00630 Glyoxylate and dicarboxylate metabolism | i0_LQ_c104330_f1p1_837_m.4398 | 221 | Peroxisomal (S)-2-hydroxy-acid oxidase GLO2 |
| sind00630 Glyoxylate and dicarboxylate metabolism | 11907_0.path0_m.2413 | 311 | Glutamine synthetase cytosolic isozyme |
| sind00630 Glyoxylate and dicarboxylate metabolism | 2789_0.path4_m.5221 | 83 | Aconitate hydratase 2, mitochondrial |
| sind00630 Glyoxylate and dicarboxylate metabolism | 8673_0.path2_m.4855 | 11 | Ribulose bisphosphate carboxylase small chain, chloroplastic |
| sind00630 Glyoxylate and dicarboxylate metabolism | 8673_0.path2_m.4855 | 107 | Ribulose bisphosphate carboxylase small chain, chloroplastic |
| sind00630 Glyoxylate and dicarboxylate metabolism | 6440_0.path0_m.2112 | 312 | Glutamine synthetase cytosolic isozyme |
| sind00630 Glyoxylate and dicarboxylate metabolism | 6440_0.path0_m.2112 | 289 | Glutamine synthetase cytosolic isozyme |
| sind00630 Glyoxylate and dicarboxylate metabolism | 6440_0.path0_m.2112 | 322 | Glutamine synthetase cytosolic isozyme |
| sind00630 Glyoxylate and dicarboxylate metabolism | 10683_0.path0_m.953 | 261 | Formate dehydrogenase, mitochondrial |
| sind00630 Glyoxylate and dicarboxylate metabolism | 5827_0.path3_m.1058 | 151 | Peroxisomal (S)-2-hydroxy-acid oxidase GLO1 |
| sind00630 Glyoxylate and dicarboxylate metabolism | i1_LQ_c77746_f1p0_1530_m.131 | 223 | Probable acetyl-CoA acetyltransferase, cytosolic 2 |
| sind00630 Glyoxylate and dicarboxylate metabolism | 5592_0.path0_m.933 | 306 | Dihydrolipoyl dehydrogenase 1, mitochondrial |
| sind00630 Glyoxylate and dicarboxylate metabolism | 5592_0.path0_m.933 | 181 | Dihydrolipoyl dehydrogenase 1, mitochondrial |
| sind00630 Glyoxylate and dicarboxylate metabolism | 5592_0.path0_m.933 | 207 | Dihydrolipoyl dehydrogenase 1, mitochondrial |
| sind00630 Glyoxylate and dicarboxylate metabolism | 5592_0.path0_m.933 | 97 | Dihydrolipoyl dehydrogenase 1, mitochondrial |
| sind00630 Glyoxylate and dicarboxylate metabolism | 5592_0.path0_m.933 | 218 | Dihydrolipoyl dehydrogenase 1, mitochondrial |
| sind00630 Glyoxylate and dicarboxylate metabolism | 5592_0.path0_m.933 | 176 | Dihydrolipoyl dehydrogenase 1, mitochondrial |
| sind00630 Glyoxylate and dicarboxylate metabolism | 5592_0.path0_m.933 | 448 | Dihydrolipoyl dehydrogenase 1, mitochondrial |
| sind00630 Glyoxylate and dicarboxylate metabolism | 5592_0.path0_m.933 | 205 | Dihydrolipoyl dehydrogenase 1, mitochondrial |
| sind00630 Glyoxylate and dicarboxylate metabolism | 5592_0.path0_m.933 | 166 | Dihydrolipoyl dehydrogenase 1, mitochondrial |
| sind00630 Glyoxylate and dicarboxylate metabolism | 5592_0.path0_m.933 | 173 | Dihydrolipoyl dehydrogenase 1, mitochondrial |
| sind00630 Glyoxylate and dicarboxylate metabolism | 5592_0.path0_m.933 | 354 | Dihydrolipoyl dehydrogenase 1, mitochondrial |
| sind00630 Glyoxylate and dicarboxylate metabolism | 5592_0.path0_m.933 | 133 | Dihydrolipoyl dehydrogenase 1, mitochondrial |
| sind00630 Glyoxylate and dicarboxylate metabolism | i0_LQ_c35465_f1p1_640_m.6645 | 115 | Aminomethyltransferase, mitochondrial |
| sind00630 Glyoxylate and dicarboxylate metabolism | i1_HQ_c41079_f2p0_1865_m.5127 | 54 | Glutamate--glyoxylate aminotransferase 2 |
| sind00630 Glyoxylate and dicarboxylate metabolism | 1589_0.path0_m.3099 | 128 | Aconitate hydratase 2, mitochondrial |
| sind00630 Glyoxylate and dicarboxylate metabolism | 1589_0.path0_m.3099 | 122 | Aconitate hydratase 2, mitochondrial |
| sind00630 Glyoxylate and dicarboxylate metabolism | 1589_0.path0_m.3099 | 558 | Aconitate hydratase 2, mitochondrial |
| sind00630 Glyoxylate and dicarboxylate metabolism | 1589_0.path0_m.3099 | 381 | Aconitate hydratase 2, mitochondrial |
| sind00630 Glyoxylate and dicarboxylate metabolism | 1589_0.path0_m.3099 | 114 | Aconitate hydratase 2, mitochondrial |
| sind00630 Glyoxylate and dicarboxylate metabolism | 1589_0.path0_m.3099 | 166 | Aconitate hydratase 2, mitochondrial |
| sind00630 Glyoxylate and dicarboxylate metabolism | 1589_0.path0_m.3099 | 844 | Aconitate hydratase 2, mitochondrial |
| sind00630 Glyoxylate and dicarboxylate metabolism | 1589_0.path0_m.3099 | 855 | Aconitate hydratase 2, mitochondrial |
| sind00630 Glyoxylate and dicarboxylate metabolism | 1589_0.path0_m.3099 | 508 | Aconitate hydratase 2, mitochondrial |
| sind00630 Glyoxylate and dicarboxylate metabolism | 1589_0.path0_m.3099 | 574 | Aconitate hydratase 2, mitochondrial |
| sind00630 Glyoxylate and dicarboxylate metabolism | 5638_0.path0_m.1009 | 190 | Ribulose bisphosphate carboxylase large chain |
| sind00630 Glyoxylate and dicarboxylate metabolism | 5638_0.path0_m.1009 | 182 | Ribulose bisphosphate carboxylase large chain |
| sind00630 Glyoxylate and dicarboxylate metabolism | 5638_0.path0_m.1009 | 39 | Ribulose bisphosphate carboxylase large chain |
| sind00630 Glyoxylate and dicarboxylate metabolism | 5638_0.path0_m.1009 | 341 | Ribulose bisphosphate carboxylase large chain |
| sind00630 Glyoxylate and dicarboxylate metabolism | 5638_0.path0_m.1009 | 153 | Ribulose bisphosphate carboxylase large chain |
| sind00630 Glyoxylate and dicarboxylate metabolism | 9685_0.path0_m.6107 | 401 | Serine hydroxymethyltransferase, mitochondrial |
| sind00630 Glyoxylate and dicarboxylate metabolism | 9685_0.path0_m.6107 | 412 | Serine hydroxymethyltransferase, mitochondrial |
| sind00630 Glyoxylate and dicarboxylate metabolism | 9685_0.path0_m.6107 | 185 | Serine hydroxymethyltransferase, mitochondrial |
| sind00630 Glyoxylate and dicarboxylate metabolism | 9685_0.path0_m.6107 | 471 | Serine hydroxymethyltransferase, mitochondrial |
| sind00630 Glyoxylate and dicarboxylate metabolism | 9685_0.path0_m.6107 | 507 | Serine hydroxymethyltransferase, mitochondrial |
| sind00630 Glyoxylate and dicarboxylate metabolism | 9538_0.path0_m.5901 | 354 | Glutamine synthetase, chloroplastic |
| sind00630 Glyoxylate and dicarboxylate metabolism | 9538_0.path0_m.5901 | 101 | Glutamine synthetase, chloroplastic |
| sind00630 Glyoxylate and dicarboxylate metabolism | 9538_0.path0_m.5901 | 168 | Glutamine synthetase, chloroplastic |
| sind00630 Glyoxylate and dicarboxylate metabolism | 11093_0.path0_m.1258 | 154 | Glycine cleavage system H protein 3, mitochondrial |
| sind00630 Glyoxylate and dicarboxylate metabolism | 11093_0.path0_m.1258 | 140 | Glycine cleavage system H protein 3, mitochondrial |
| sind00630 Glyoxylate and dicarboxylate metabolism | 11093_0.path0_m.1258 | 47 | Glycine cleavage system H protein 3, mitochondrial |
| sind00630 Glyoxylate and dicarboxylate metabolism | 9257_0.path1_m.5500 | 401 | Citrate synthase, mitochondrial |
| sind00630 Glyoxylate and dicarboxylate metabolism | 9257_0.path1_m.5500 | 346 | Citrate synthase, mitochondrial |
| sind00480 Glutathione metabolism | 7028_0.path1_m.2813 | 265 | Glutathione S-transferase DHAR3, chloroplastic |
| sind00480 Glutathione metabolism | i1_LQ_c36007_f1p0_1069_m.13 | 191 | Protein IN2-1 homolog B |
| sind00480 Glutathione metabolism | i1_LQ_c36007_f1p0_1069_m.13 | 204 | Protein IN2-1 homolog B |
| sind00480 Glutathione metabolism | i1_LQ_c36007_f1p0_1069_m.13 | 70 | Protein IN2-1 homolog B |
| sind00480 Glutathione metabolism | i1_LQ_c36007_f1p0_1069_m.13 | 230 | Protein IN2-1 homolog B |
| sind00480 Glutathione metabolism | i2_LQ_c96775_f1p0_2002_m.4473 | 55 | Glutathione S-transferase F11 |
| sind00480 Glutathione metabolism | 8750_0.path0_m.4965 | 267 | L-ascorbate peroxidase 3, peroxisomal |
| sind00480 Glutathione metabolism | 7428_0.path0_m.3131 | 208 | Glutathione S-transferase T1 |
| sind00480 Glutathione metabolism | 3549_0.path0_m.6407 | 251 | Leucine aminopeptidase 2, chloroplastic |
| sind00480 Glutathione metabolism | 5772_0.path0_m.1247 | 365 | L-ascorbate peroxidase T, chloroplastic |
| sind00480 Glutathione metabolism | 5772_0.path0_m.1247 | 345 | L-ascorbate peroxidase T, chloroplastic |
| sind00480 Glutathione metabolism | 5772_0.path0_m.1247 | 103 | L-ascorbate peroxidase T, chloroplastic |
| sind00480 Glutathione metabolism | 5772_0.path0_m.1247 | 144 | L-ascorbate peroxidase T, chloroplastic |
| sind00480 Glutathione metabolism | 4520_0.path2_m.7505 | 185 | Glutathione reductase, chloroplastic (Fragment) |
| sind00051 Fructose and mannose metabolism | 10152_0.path0_m.229 | 369 | Fructose-bisphosphate aldolase 2, chloroplastic |
| sind00051 Fructose and mannose metabolism | 10152_0.path0_m.229 | 61 | Fructose-bisphosphate aldolase 2, chloroplastic |
| sind00051 Fructose and mannose metabolism | 11884_0.path0_m.2391 | 40 | Fructose-bisphosphate aldolase 6, cytosolic |
| sind00051 Fructose and mannose metabolism | 11884_0.path0_m.2391 | 357 | Fructose-bisphosphate aldolase 6, cytosolic |
| sind01230 Biosynthesis of amino acids | i1_HQ_c41079_f2p0_1865_m.5127 | 54 | Glutamate--glyoxylate aminotransferase 2 |
| sind01230 Biosynthesis of amino acids | 4658_0.path0_m.7768 | 148 | Phosphoglycerate kinase, chloroplastic |
| sind01230 Biosynthesis of amino acids | 9209_0.path0_m.5429 | 228 | Citrate synthase, glyoxysomal |
| sind01230 Biosynthesis of amino acids | i1_HQ_c14606_f2p0_1675_m.3241 | 179 | Aspartate aminotransferase, mitochondrial |
| sind01230 Biosynthesis of amino acids | i1_HQ_c14606_f2p0_1675_m.3241 | 374 | Aspartate aminotransferase, mitochondrial |
| sind01230 Biosynthesis of amino acids | 1589_0.path0_m.3099 | 128 | Aconitate hydratase 2, mitochondrial |
| sind01230 Biosynthesis of amino acids | 1589_0.path0_m.3099 | 122 | Aconitate hydratase 2, mitochondrial |
| sind01230 Biosynthesis of amino acids | 1589_0.path0_m.3099 | 558 | Aconitate hydratase 2, mitochondrial |
| sind01230 Biosynthesis of amino acids | 1589_0.path0_m.3099 | 381 | Aconitate hydratase 2, mitochondrial |
| sind01230 Biosynthesis of amino acids | 1589_0.path0_m.3099 | 114 | Aconitate hydratase 2, mitochondrial |
| sind01230 Biosynthesis of amino acids | 1589_0.path0_m.3099 | 166 | Aconitate hydratase 2, mitochondrial |
| sind01230 Biosynthesis of amino acids | 1589_0.path0_m.3099 | 844 | Aconitate hydratase 2, mitochondrial |
| sind01230 Biosynthesis of amino acids | 1589_0.path0_m.3099 | 855 | Aconitate hydratase 2, mitochondrial |
| sind01230 Biosynthesis of amino acids | 1589_0.path0_m.3099 | 508 | Aconitate hydratase 2, mitochondrial |
| sind01230 Biosynthesis of amino acids | 1589_0.path0_m.3099 | 574 | Aconitate hydratase 2, mitochondrial |
| sind01230 Biosynthesis of amino acids | i1_HQ_c60950_f14p0_1160_m.1131 | 220 | Cysteine synthase |
| sind01230 Biosynthesis of amino acids | 8601_0.path0_m.4749 | 205 | Isocitrate dehydrogenase [NAD] catalytic subunit 5, mitochondrial |
| sind01230 Biosynthesis of amino acids | 8601_0.path0_m.4749 | 332 | Isocitrate dehydrogenase [NAD] catalytic subunit 5, mitochondrial |
| sind01230 Biosynthesis of amino acids | 8601_0.path0_m.4749 | 168 | Isocitrate dehydrogenase [NAD] catalytic subunit 5, mitochondrial |
| sind01230 Biosynthesis of amino acids | 11907_0.path0_m.2413 | 311 | Glutamine synthetase cytosolic isozyme |
| sind01230 Biosynthesis of amino acids | 2789_0.path4_m.5221 | 83 | Aconitate hydratase 2, mitochondrial |
| sind01230 Biosynthesis of amino acids | 9685_0.path0_m.6107 | 401 | Serine hydroxymethyltransferase, mitochondrial |
| sind01230 Biosynthesis of amino acids | 9685_0.path0_m.6107 | 412 | Serine hydroxymethyltransferase, mitochondrial |
| sind01230 Biosynthesis of amino acids | 9685_0.path0_m.6107 | 185 | Serine hydroxymethyltransferase, mitochondrial |
| sind01230 Biosynthesis of amino acids | 9685_0.path0_m.6107 | 471 | Serine hydroxymethyltransferase, mitochondrial |
| sind01230 Biosynthesis of amino acids | 9685_0.path0_m.6107 | 507 | Serine hydroxymethyltransferase, mitochondrial |
| sind01230 Biosynthesis of amino acids | 9538_0.path0_m.5901 | 354 | Glutamine synthetase, chloroplastic |
| sind01230 Biosynthesis of amino acids | 9538_0.path0_m.5901 | 101 | Glutamine synthetase, chloroplastic |
| sind01230 Biosynthesis of amino acids | 9538_0.path0_m.5901 | 168 | Glutamine synthetase, chloroplastic |
| sind01230 Biosynthesis of amino acids | 6440_0.path0_m.2112 | 312 | Glutamine synthetase cytosolic isozyme |
| sind01230 Biosynthesis of amino acids | 6440_0.path0_m.2112 | 289 | Glutamine synthetase cytosolic isozyme |
| sind01230 Biosynthesis of amino acids | 6440_0.path0_m.2112 | 322 | Glutamine synthetase cytosolic isozyme |
| sind01230 Biosynthesis of amino acids | 10152_0.path0_m.229 | 369 | Fructose-bisphosphate aldolase 2, chloroplastic |
| sind01230 Biosynthesis of amino acids | 10152_0.path0_m.229 | 61 | Fructose-bisphosphate aldolase 2, chloroplastic |
| sind01230 Biosynthesis of amino acids | 9257_0.path1_m.5500 | 401 | Citrate synthase, mitochondrial |
| sind01230 Biosynthesis of amino acids | 9257_0.path1_m.5500 | 346 | Citrate synthase, mitochondrial |
| sind01230 Biosynthesis of amino acids | i1_LQ_c67740_f1p0_1601_m.6729 | 74 | Phosphoglycerate kinase, cytosolic |
| sind01230 Biosynthesis of amino acids | 11884_0.path0_m.2391 | 40 | Fructose-bisphosphate aldolase 6, cytosolic |
| sind01230 Biosynthesis of amino acids | 11884_0.path0_m.2391 | 357 | Fructose-bisphosphate aldolase 6, cytosolic |
| sind00592 alpha-Linolenic acid metabolism | 6551_0.path0_m.2298 | 59 | Fatty acid hydroperoxide lyase, chloroplastic |
| sind00592 alpha-Linolenic acid metabolism | 5250_0.path0_m.317 | 709 | Glyoxysomal fatty acid beta-oxidation multifunctional protein MFP-a |
| sind00592 alpha-Linolenic acid metabolism | 5250_0.path0_m.317 | 374 | Glyoxysomal fatty acid beta-oxidation multifunctional protein MFP-a |
| sind00592 alpha-Linolenic acid metabolism | 5250_0.path0_m.317 | 355 | Glyoxysomal fatty acid beta-oxidation multifunctional protein MFP-a |
| sind00640 Propanoate metabolism | i1_HQ_c32623_f2p0_1256_m.6619 | 79 | Succinate--CoA ligase [ADP-forming] subunit alpha-2, mitochondrial |
| sind00640 Propanoate metabolism | 5856_0.path0_m.1092 | 163 | Succinate--CoA ligase [ADP-forming] subunit beta, mitochondrial |
| sind00640 Propanoate metabolism | 5856_0.path0_m.1092 | 119 | Succinate--CoA ligase [ADP-forming] subunit beta, mitochondrial |
| sind00640 Propanoate metabolism | 5856_0.path0_m.1092 | 205 | Succinate--CoA ligase [ADP-forming] subunit beta, mitochondrial |
| sind00640 Propanoate metabolism | 5856_0.path0_m.1092 | 174 | Succinate--CoA ligase [ADP-forming] subunit beta, mitochondrial |
| sind00640 Propanoate metabolism | 5856_0.path0_m.1092 | 89 | Succinate--CoA ligase [ADP-forming] subunit beta, mitochondrial |
| sind00640 Propanoate metabolism | 6655_0.path0_m.2208 | 77 | Succinate--CoA ligase [ADP-forming] subunit alpha-2, mitochondrial |
| sind00640 Propanoate metabolism | i1_LQ_c14245_f1p3_1457_m.1128 | 279 | 3-hydroxyisobutyryl-CoA hydrolase-like protein 3, mitochondrial |
| sind00640 Propanoate metabolism | i1_LQ_c77746_f1p0_1530_m.131 | 223 | Probable acetyl-CoA acetyltransferase, cytosolic 2 |
| sind00640 Propanoate metabolism | 5592_0.path0_m.933 | 306 | Dihydrolipoyl dehydrogenase 1, mitochondrial |
| sind00640 Propanoate metabolism | 5592_0.path0_m.933 | 181 | Dihydrolipoyl dehydrogenase 1, mitochondrial |
| sind00640 Propanoate metabolism | 5592_0.path0_m.933 | 207 | Dihydrolipoyl dehydrogenase 1, mitochondrial |
| sind00640 Propanoate metabolism | 5592_0.path0_m.933 | 97 | Dihydrolipoyl dehydrogenase 1, mitochondrial |
| sind00640 Propanoate metabolism | 5592_0.path0_m.933 | 218 | Dihydrolipoyl dehydrogenase 1, mitochondrial |
| sind00640 Propanoate metabolism | 5592_0.path0_m.933 | 176 | Dihydrolipoyl dehydrogenase 1, mitochondrial |
| sind00640 Propanoate metabolism | 5592_0.path0_m.933 | 448 | Dihydrolipoyl dehydrogenase 1, mitochondrial |
| sind00640 Propanoate metabolism | 5592_0.path0_m.933 | 205 | Dihydrolipoyl dehydrogenase 1, mitochondrial |
| sind00640 Propanoate metabolism | 5592_0.path0_m.933 | 166 | Dihydrolipoyl dehydrogenase 1, mitochondrial |
| sind00640 Propanoate metabolism | 5592_0.path0_m.933 | 173 | Dihydrolipoyl dehydrogenase 1, mitochondrial |
| sind00640 Propanoate metabolism | 5592_0.path0_m.933 | 354 | Dihydrolipoyl dehydrogenase 1, mitochondrial |
| sind00640 Propanoate metabolism | 5592_0.path0_m.933 | 133 | Dihydrolipoyl dehydrogenase 1, mitochondrial |
| sind00020 Citrate cycle (TCA cycle) | 5856_0.path0_m.1092 | 163 | Succinate--CoA ligase [ADP-forming] subunit beta, mitochondrial |
| sind00020 Citrate cycle (TCA cycle) | 5856_0.path0_m.1092 | 119 | Succinate--CoA ligase [ADP-forming] subunit beta, mitochondrial |
| sind00020 Citrate cycle (TCA cycle) | 5856_0.path0_m.1092 | 205 | Succinate--CoA ligase [ADP-forming] subunit beta, mitochondrial |
| sind00020 Citrate cycle (TCA cycle) | 5856_0.path0_m.1092 | 174 | Succinate--CoA ligase [ADP-forming] subunit beta, mitochondrial |
| sind00020 Citrate cycle (TCA cycle) | 5856_0.path0_m.1092 | 89 | Succinate--CoA ligase [ADP-forming] subunit beta, mitochondrial |
| sind00020 Citrate cycle (TCA cycle) | 4897_0.path0_m.8229 | 164 | Succinate dehydrogenase [ubiquinone] flavoprotein subunit 1, mitochondrial |
| sind00020 Citrate cycle (TCA cycle) | 4897_0.path0_m.8229 | 318 | Succinate dehydrogenase [ubiquinone] flavoprotein subunit 1, mitochondrial |
| sind00020 Citrate cycle (TCA cycle) | 9209_0.path0_m.5429 | 228 | Citrate synthase, glyoxysomal |
| sind00020 Citrate cycle (TCA cycle) | 1931_0.path2_m.3930 | 373 | 2-oxoglutarate dehydrogenase, mitochondrial |
| sind00020 Citrate cycle (TCA cycle) | 1931_0.path2_m.3930 | 437 | 2-oxoglutarate dehydrogenase, mitochondrial |
| sind00020 Citrate cycle (TCA cycle) | 1931_0.path2_m.3930 | 446 | 2-oxoglutarate dehydrogenase, mitochondrial |
| sind00020 Citrate cycle (TCA cycle) | 1931_0.path2_m.3930 | 218 | 2-oxoglutarate dehydrogenase, mitochondrial |
| sind00020 Citrate cycle (TCA cycle) | 1931_0.path2_m.3930 | 392 | 2-oxoglutarate dehydrogenase, mitochondrial |
| sind00020 Citrate cycle (TCA cycle) | 1931_0.path2_m.3930 | 362 | 2-oxoglutarate dehydrogenase, mitochondrial |
| sind00020 Citrate cycle (TCA cycle) | 1931_0.path2_m.3930 | 459 | 2-oxoglutarate dehydrogenase, mitochondrial |
| sind00020 Citrate cycle (TCA cycle) | 1931_0.path2_m.3930 | 843 | 2-oxoglutarate dehydrogenase, mitochondrial |
| sind00020 Citrate cycle (TCA cycle) | 8601_0.path0_m.4749 | 205 | Isocitrate dehydrogenase [NAD] catalytic subunit 5, mitochondrial |
| sind00020 Citrate cycle (TCA cycle) | 8601_0.path0_m.4749 | 332 | Isocitrate dehydrogenase [NAD] catalytic subunit 5, mitochondrial |
| sind00020 Citrate cycle (TCA cycle) | 8601_0.path0_m.4749 | 168 | Isocitrate dehydrogenase [NAD] catalytic subunit 5, mitochondrial |
| sind00020 Citrate cycle (TCA cycle) | 4752_1.path0_m.7937 | 124 | Dihydrolipoyllysine-residue succinyltransferase component of 2-oxoglutarate dehydrogenase complex 2, mitochondrial |
| sind00020 Citrate cycle (TCA cycle) | 4752_1.path0_m.7937 | 322 | Dihydrolipoyllysine-residue succinyltransferase component of 2-oxoglutarate dehydrogenase complex 2, mitochondrial |
| sind00020 Citrate cycle (TCA cycle) | 4752_1.path0_m.7937 | 249 | Dihydrolipoyllysine-residue succinyltransferase component of 2-oxoglutarate dehydrogenase complex 2, mitochondrial |
| sind00020 Citrate cycle (TCA cycle) | 2789_0.path4_m.5221 | 83 | Aconitate hydratase 2, mitochondrial |
| sind00020 Citrate cycle (TCA cycle) | 9666_0.path0_m.6084 | 157 | Dihydrolipoyllysine-residue acetyltransferase component 3 of pyruvate dehydrogenase complex, mitochondrial |
| sind00020 Citrate cycle (TCA cycle) | 9666_0.path0_m.6084 | 150 | Dihydrolipoyllysine-residue acetyltransferase component 3 of pyruvate dehydrogenase complex, mitochondrial |
| sind00020 Citrate cycle (TCA cycle) | i1_HQ_c32623_f2p0_1256_m.6619 | 79 | Succinate--CoA ligase [ADP-forming] subunit alpha-2, mitochondrial |
| sind00020 Citrate cycle (TCA cycle) | 1242_0.path10_m.2975 | 613 | 2-oxoglutarate dehydrogenase, mitochondrial |
| sind00020 Citrate cycle (TCA cycle) | 1242_0.path10_m.2975 | 603 | 2-oxoglutarate dehydrogenase, mitochondrial |
| sind00020 Citrate cycle (TCA cycle) | 6944_0.path0_m.2670 | 16 | ATP-citrate synthase alpha chain protein 2 |
| sind00020 Citrate cycle (TCA cycle) | 6944_0.path0_m.2670 | 58 | ATP-citrate synthase alpha chain protein 2 |
| sind00020 Citrate cycle (TCA cycle) | 2221_0.path0_m.4534 | 604 | 2-oxoglutarate dehydrogenase, mitochondrial |
| sind00020 Citrate cycle (TCA cycle) | 5592_0.path0_m.933 | 306 | Dihydrolipoyl dehydrogenase 1, mitochondrial |
| sind00020 Citrate cycle (TCA cycle) | 5592_0.path0_m.933 | 181 | Dihydrolipoyl dehydrogenase 1, mitochondrial |
| sind00020 Citrate cycle (TCA cycle) | 5592_0.path0_m.933 | 207 | Dihydrolipoyl dehydrogenase 1, mitochondrial |
| sind00020 Citrate cycle (TCA cycle) | 5592_0.path0_m.933 | 97 | Dihydrolipoyl dehydrogenase 1, mitochondrial |
| sind00020 Citrate cycle (TCA cycle) | 5592_0.path0_m.933 | 218 | Dihydrolipoyl dehydrogenase 1, mitochondrial |
| sind00020 Citrate cycle (TCA cycle) | 5592_0.path0_m.933 | 176 | Dihydrolipoyl dehydrogenase 1, mitochondrial |
| sind00020 Citrate cycle (TCA cycle) | 5592_0.path0_m.933 | 448 | Dihydrolipoyl dehydrogenase 1, mitochondrial |
| sind00020 Citrate cycle (TCA cycle) | 5592_0.path0_m.933 | 205 | Dihydrolipoyl dehydrogenase 1, mitochondrial |
| sind00020 Citrate cycle (TCA cycle) | 5592_0.path0_m.933 | 166 | Dihydrolipoyl dehydrogenase 1, mitochondrial |
| sind00020 Citrate cycle (TCA cycle) | 5592_0.path0_m.933 | 173 | Dihydrolipoyl dehydrogenase 1, mitochondrial |
| sind00020 Citrate cycle (TCA cycle) | 5592_0.path0_m.933 | 354 | Dihydrolipoyl dehydrogenase 1, mitochondrial |
| sind00020 Citrate cycle (TCA cycle) | 5592_0.path0_m.933 | 133 | Dihydrolipoyl dehydrogenase 1, mitochondrial |
| sind00020 Citrate cycle (TCA cycle) | 6655_0.path0_m.2208 | 77 | Succinate--CoA ligase [ADP-forming] subunit alpha-2, mitochondrial |
| sind00020 Citrate cycle (TCA cycle) | 1589_0.path0_m.3099 | 128 | Aconitate hydratase 2, mitochondrial |
| sind00020 Citrate cycle (TCA cycle) | 1589_0.path0_m.3099 | 122 | Aconitate hydratase 2, mitochondrial |
| sind00020 Citrate cycle (TCA cycle) | 1589_0.path0_m.3099 | 558 | Aconitate hydratase 2, mitochondrial |
| sind00020 Citrate cycle (TCA cycle) | 1589_0.path0_m.3099 | 381 | Aconitate hydratase 2, mitochondrial |
| sind00020 Citrate cycle (TCA cycle) | 1589_0.path0_m.3099 | 114 | Aconitate hydratase 2, mitochondrial |
| sind00020 Citrate cycle (TCA cycle) | 1589_0.path0_m.3099 | 166 | Aconitate hydratase 2, mitochondrial |
| sind00020 Citrate cycle (TCA cycle) | 1589_0.path0_m.3099 | 844 | Aconitate hydratase 2, mitochondrial |
| sind00020 Citrate cycle (TCA cycle) | 1589_0.path0_m.3099 | 855 | Aconitate hydratase 2, mitochondrial |
| sind00020 Citrate cycle (TCA cycle) | 1589_0.path0_m.3099 | 508 | Aconitate hydratase 2, mitochondrial |
| sind00020 Citrate cycle (TCA cycle) | 1589_0.path0_m.3099 | 574 | Aconitate hydratase 2, mitochondrial |
| sind00020 Citrate cycle (TCA cycle) | 9636_0.path0_m.6039 | 76 | Pyruvate dehydrogenase E1 component subunit beta-1, mitochondrial |
| sind00020 Citrate cycle (TCA cycle) | 7112_0.path0_m.2939 | 371 | Pyruvate dehydrogenase E1 component subunit alpha, mitochondrial |
| sind00020 Citrate cycle (TCA cycle) | 7112_0.path0_m.2939 | 333 | Pyruvate dehydrogenase E1 component subunit alpha, mitochondrial |
| sind00020 Citrate cycle (TCA cycle) | 7112_0.path0_m.2939 | 354 | Pyruvate dehydrogenase E1 component subunit alpha, mitochondrial |
| sind00020 Citrate cycle (TCA cycle) | 7112_0.path0_m.2939 | 239 | Pyruvate dehydrogenase E1 component subunit alpha, mitochondrial |
| sind00020 Citrate cycle (TCA cycle) | 7112_0.path0_m.2939 | 322 | Pyruvate dehydrogenase E1 component subunit alpha, mitochondrial |
| sind00020 Citrate cycle (TCA cycle) | 9257_0.path1_m.5500 | 401 | Citrate synthase, mitochondrial |
| sind00020 Citrate cycle (TCA cycle) | 9257_0.path1_m.5500 | 346 | Citrate synthase, mitochondrial |
| sind00020 Citrate cycle (TCA cycle) | 5891_0.path0_m.1161 | 266 | ATP-citrate synthase beta chain protein 2 |
| sind00020 Citrate cycle (TCA cycle) | i1_LQ_c26180_f2p0_1342_m.6236 | 203 | Dihydrolipoyllysine-residue acetyltransferase component 1 of pyruvate dehydrogenase complex, mitochondrial |
| sind00380 Tryptophan metabolism | 1242_0.path10_m.2975 | 613 | 2-oxoglutarate dehydrogenase, mitochondrial |
| sind00380 Tryptophan metabolism | 1242_0.path10_m.2975 | 603 | 2-oxoglutarate dehydrogenase, mitochondrial |
| sind00380 Tryptophan metabolism | 2221_0.path0_m.4534 | 604 | 2-oxoglutarate dehydrogenase, mitochondrial |
| sind00380 Tryptophan metabolism | 1931_0.path2_m.3930 | 373 | 2-oxoglutarate dehydrogenase, mitochondrial |
| sind00380 Tryptophan metabolism | 1931_0.path2_m.3930 | 437 | 2-oxoglutarate dehydrogenase, mitochondrial |
| sind00380 Tryptophan metabolism | 1931_0.path2_m.3930 | 446 | 2-oxoglutarate dehydrogenase, mitochondrial |
| sind00380 Tryptophan metabolism | 1931_0.path2_m.3930 | 218 | 2-oxoglutarate dehydrogenase, mitochondrial |
| sind00380 Tryptophan metabolism | 1931_0.path2_m.3930 | 392 | 2-oxoglutarate dehydrogenase, mitochondrial |
| sind00380 Tryptophan metabolism | 1931_0.path2_m.3930 | 362 | 2-oxoglutarate dehydrogenase, mitochondrial |
| sind00380 Tryptophan metabolism | 1931_0.path2_m.3930 | 459 | 2-oxoglutarate dehydrogenase, mitochondrial |
| sind00380 Tryptophan metabolism | 1931_0.path2_m.3930 | 843 | 2-oxoglutarate dehydrogenase, mitochondrial |
| sind00380 Tryptophan metabolism | i1_LQ_c77746_f1p0_1530_m.131 | 223 | Probable acetyl-CoA acetyltransferase, cytosolic 2 |
| sind00670 One carbon pool by folate | i0_LQ_c35465_f1p1_640_m.6645 | 115 | Aminomethyltransferase, mitochondrial |
| sind00670 One carbon pool by folate | 9685_0.path0_m.6107 | 401 | Serine hydroxymethyltransferase, mitochondrial |
| sind00670 One carbon pool by folate | 9685_0.path0_m.6107 | 412 | Serine hydroxymethyltransferase, mitochondrial |
| sind00670 One carbon pool by folate | 9685_0.path0_m.6107 | 185 | Serine hydroxymethyltransferase, mitochondrial |
| sind00670 One carbon pool by folate | 9685_0.path0_m.6107 | 471 | Serine hydroxymethyltransferase, mitochondrial |
| sind00670 One carbon pool by folate | 9685_0.path0_m.6107 | 507 | Serine hydroxymethyltransferase, mitochondrial |
| sind00310 Lysine degradation | 1242_0.path10_m.2975 | 613 | 2-oxoglutarate dehydrogenase, mitochondrial |
| sind00310 Lysine degradation | 1242_0.path10_m.2975 | 603 | 2-oxoglutarate dehydrogenase, mitochondrial |
| sind00310 Lysine degradation | 2221_0.path0_m.4534 | 604 | 2-oxoglutarate dehydrogenase, mitochondrial |
| sind00310 Lysine degradation | 1931_0.path2_m.3930 | 373 | 2-oxoglutarate dehydrogenase, mitochondrial |
| sind00310 Lysine degradation | 1931_0.path2_m.3930 | 437 | 2-oxoglutarate dehydrogenase, mitochondrial |
| sind00310 Lysine degradation | 1931_0.path2_m.3930 | 446 | 2-oxoglutarate dehydrogenase, mitochondrial |
| sind00310 Lysine degradation | 1931_0.path2_m.3930 | 218 | 2-oxoglutarate dehydrogenase, mitochondrial |
| sind00310 Lysine degradation | 1931_0.path2_m.3930 | 392 | 2-oxoglutarate dehydrogenase, mitochondrial |
| sind00310 Lysine degradation | 1931_0.path2_m.3930 | 362 | 2-oxoglutarate dehydrogenase, mitochondrial |
| sind00310 Lysine degradation | 1931_0.path2_m.3930 | 459 | 2-oxoglutarate dehydrogenase, mitochondrial |
| sind00310 Lysine degradation | 1931_0.path2_m.3930 | 843 | 2-oxoglutarate dehydrogenase, mitochondrial |
| sind00310 Lysine degradation | i1_LQ_c77746_f1p0_1530_m.131 | 223 | Probable acetyl-CoA acetyltransferase, cytosolic 2 |
| sind00310 Lysine degradation | 4752_1.path0_m.7937 | 124 | Dihydrolipoyllysine-residue succinyltransferase component of 2-oxoglutarate dehydrogenase complex 2, mitochondrial |
| sind00310 Lysine degradation | 4752_1.path0_m.7937 | 322 | Dihydrolipoyllysine-residue succinyltransferase component of 2-oxoglutarate dehydrogenase complex 2, mitochondrial |
| sind00310 Lysine degradation | 4752_1.path0_m.7937 | 249 | Dihydrolipoyllysine-residue succinyltransferase component of 2-oxoglutarate dehydrogenase complex 2, mitochondrial |
| sind00010 Glycolysis / Gluconeogenesis | 9666_0.path0_m.6084 | 157 | Dihydrolipoyllysine-residue acetyltransferase component 3 of pyruvate dehydrogenase complex, mitochondrial |
| sind00010 Glycolysis / Gluconeogenesis | 9666_0.path0_m.6084 | 150 | Dihydrolipoyllysine-residue acetyltransferase component 3 of pyruvate dehydrogenase complex, mitochondrial |
| sind00010 Glycolysis / Gluconeogenesis | 4658_0.path0_m.7768 | 148 | Phosphoglycerate kinase, chloroplastic |
| sind00010 Glycolysis / Gluconeogenesis | 7112_0.path0_m.2939 | 371 | Pyruvate dehydrogenase E1 component subunit alpha, mitochondrial |
| sind00010 Glycolysis / Gluconeogenesis | 7112_0.path0_m.2939 | 333 | Pyruvate dehydrogenase E1 component subunit alpha, mitochondrial |
| sind00010 Glycolysis / Gluconeogenesis | 7112_0.path0_m.2939 | 354 | Pyruvate dehydrogenase E1 component subunit alpha, mitochondrial |
| sind00010 Glycolysis / Gluconeogenesis | 7112_0.path0_m.2939 | 239 | Pyruvate dehydrogenase E1 component subunit alpha, mitochondrial |
| sind00010 Glycolysis / Gluconeogenesis | 7112_0.path0_m.2939 | 322 | Pyruvate dehydrogenase E1 component subunit alpha, mitochondrial |
| sind00010 Glycolysis / Gluconeogenesis | 10152_0.path0_m.229 | 369 | Fructose-bisphosphate aldolase 2, chloroplastic |
| sind00010 Glycolysis / Gluconeogenesis | 10152_0.path0_m.229 | 61 | Fructose-bisphosphate aldolase 2, chloroplastic |
| sind00010 Glycolysis / Gluconeogenesis | i1_LQ_c67740_f1p0_1601_m.6729 | 74 | Phosphoglycerate kinase, cytosolic |
| sind00010 Glycolysis / Gluconeogenesis | 11884_0.path0_m.2391 | 40 | Fructose-bisphosphate aldolase 6, cytosolic |
| sind00010 Glycolysis / Gluconeogenesis | 11884_0.path0_m.2391 | 357 | Fructose-bisphosphate aldolase 6, cytosolic |
| sind00010 Glycolysis / Gluconeogenesis | i1_LQ_c26180_f2p0_1342_m.6236 | 203 | Dihydrolipoyllysine-residue acetyltransferase component 1 of pyruvate dehydrogenase complex, mitochondrial |
| sind00010 Glycolysis / Gluconeogenesis | 9636_0.path0_m.6039 | 76 | Pyruvate dehydrogenase E1 component subunit beta-1, mitochondrial |
| sind00010 Glycolysis / Gluconeogenesis | 5592_0.path0_m.933 | 306 | Dihydrolipoyl dehydrogenase 1, mitochondrial |
| sind00010 Glycolysis / Gluconeogenesis | 5592_0.path0_m.933 | 181 | Dihydrolipoyl dehydrogenase 1, mitochondrial |
| sind00010 Glycolysis / Gluconeogenesis | 5592_0.path0_m.933 | 207 | Dihydrolipoyl dehydrogenase 1, mitochondrial |
| sind00010 Glycolysis / Gluconeogenesis | 5592_0.path0_m.933 | 97 | Dihydrolipoyl dehydrogenase 1, mitochondrial |
| sind00010 Glycolysis / Gluconeogenesis | 5592_0.path0_m.933 | 218 | Dihydrolipoyl dehydrogenase 1, mitochondrial |
| sind00010 Glycolysis / Gluconeogenesis | 5592_0.path0_m.933 | 176 | Dihydrolipoyl dehydrogenase 1, mitochondrial |
| sind00010 Glycolysis / Gluconeogenesis | 5592_0.path0_m.933 | 448 | Dihydrolipoyl dehydrogenase 1, mitochondrial |
| sind00010 Glycolysis / Gluconeogenesis | 5592_0.path0_m.933 | 205 | Dihydrolipoyl dehydrogenase 1, mitochondrial |
| sind00010 Glycolysis / Gluconeogenesis | 5592_0.path0_m.933 | 166 | Dihydrolipoyl dehydrogenase 1, mitochondrial |
| sind00010 Glycolysis / Gluconeogenesis | 5592_0.path0_m.933 | 173 | Dihydrolipoyl dehydrogenase 1, mitochondrial |
| sind00010 Glycolysis / Gluconeogenesis | 5592_0.path0_m.933 | 354 | Dihydrolipoyl dehydrogenase 1, mitochondrial |
| sind00010 Glycolysis / Gluconeogenesis | 5592_0.path0_m.933 | 133 | Dihydrolipoyl dehydrogenase 1, mitochondrial |
| sind00196 Photosynthesis - antenna proteins | 7467_0.path0_m.3184 | 238 | Chlorophyll a-b binding protein 8, chloroplastic |
| sind00196 Photosynthesis - antenna proteins | 7383_0.path0_m.3334 | 53 | Chlorophyll a-b binding protein CP24 10A, chloroplastic |
| sind00196 Photosynthesis - antenna proteins | 10025_0.path0_m.37 | 7 | Chlorophyll a-b binding protein of LHCII type I, chloroplastic (Fragment) |
| sind00196 Photosynthesis - antenna proteins | i0_HQ_c151837_f2p8_620_m.7479 | 49 | Chlorophyll a-b binding protein 6, chloroplastic |
| sind00196 Photosynthesis - antenna proteins | i0_HQ_c151837_f2p8_620_m.7479 | 62 | Chlorophyll a-b binding protein 6, chloroplastic |
| sind00196 Photosynthesis - antenna proteins | 8146_0.path0_m.4182 | 42 | Chlorophyll a-b binding protein 36, chloroplastic |
| sind00196 Photosynthesis - antenna proteins | 8146_0.path0_m.4182 | 124 | Chlorophyll a-b binding protein 36, chloroplastic |
| sind00196 Photosynthesis - antenna proteins | 7598_0.path21_m.3382 | 41 | Chlorophyll a-b binding protein of LHCII type I, chloroplastic (Fragment) |
| sind00196 Photosynthesis - antenna proteins | 8352_0.path0_m.4346 | 113 | Chlorophyll a-b binding protein CP26, chloroplastic |
| sind00910 Nitrogen metabolism | 9538_0.path0_m.5901 | 354 | Glutamine synthetase, chloroplastic |
| sind00910 Nitrogen metabolism | 9538_0.path0_m.5901 | 101 | Glutamine synthetase, chloroplastic |
| sind00910 Nitrogen metabolism | 9538_0.path0_m.5901 | 168 | Glutamine synthetase, chloroplastic |
| sind00910 Nitrogen metabolism | 6440_0.path0_m.2112 | 312 | Glutamine synthetase cytosolic isozyme |
| sind00910 Nitrogen metabolism | 6440_0.path0_m.2112 | 289 | Glutamine synthetase cytosolic isozyme |
| sind00910 Nitrogen metabolism | 6440_0.path0_m.2112 | 322 | Glutamine synthetase cytosolic isozyme |
| sind00910 Nitrogen metabolism | 11907_0.path0_m.2413 | 311 | Glutamine synthetase cytosolic isozyme |
| sind00280 Valine, leucine and isoleucine degradation | i1_LQ_c46534_f1p0_1143_m.1204 | 131 | Probable enoyl-CoA hydratase 2, mitochondrial |
| sind00280 Valine, leucine and isoleucine degradation | i1_LQ_c14245_f1p3_1457_m.1128 | 279 | 3-hydroxyisobutyryl-CoA hydrolase-like protein 3, mitochondrial |
| sind00280 Valine, leucine and isoleucine degradation | i1_LQ_c77746_f1p0_1530_m.131 | 223 | Probable acetyl-CoA acetyltransferase, cytosolic 2 |
| sind00280 Valine, leucine and isoleucine degradation | 10612_0.path0_m.864 | 64 | Probable enoyl-CoA hydratase 2, mitochondrial |
| sind00280 Valine, leucine and isoleucine degradation | 10612_0.path0_m.864 | 111 | Probable enoyl-CoA hydratase 2, mitochondrial |
| sind00280 Valine, leucine and isoleucine degradation | 5592_0.path0_m.933 | 306 | Dihydrolipoyl dehydrogenase 1, mitochondrial |
| sind00280 Valine, leucine and isoleucine degradation | 5592_0.path0_m.933 | 181 | Dihydrolipoyl dehydrogenase 1, mitochondrial |
| sind00280 Valine, leucine and isoleucine degradation | 5592_0.path0_m.933 | 207 | Dihydrolipoyl dehydrogenase 1, mitochondrial |
| sind00280 Valine, leucine and isoleucine degradation | 5592_0.path0_m.933 | 97 | Dihydrolipoyl dehydrogenase 1, mitochondrial |
| sind00280 Valine, leucine and isoleucine degradation | 5592_0.path0_m.933 | 218 | Dihydrolipoyl dehydrogenase 1, mitochondrial |
| sind00280 Valine, leucine and isoleucine degradation | 5592_0.path0_m.933 | 176 | Dihydrolipoyl dehydrogenase 1, mitochondrial |
| sind00280 Valine, leucine and isoleucine degradation | 5592_0.path0_m.933 | 448 | Dihydrolipoyl dehydrogenase 1, mitochondrial |
| sind00280 Valine, leucine and isoleucine degradation | 5592_0.path0_m.933 | 205 | Dihydrolipoyl dehydrogenase 1, mitochondrial |
| sind00280 Valine, leucine and isoleucine degradation | 5592_0.path0_m.933 | 166 | Dihydrolipoyl dehydrogenase 1, mitochondrial |
| sind00280 Valine, leucine and isoleucine degradation | 5592_0.path0_m.933 | 173 | Dihydrolipoyl dehydrogenase 1, mitochondrial |
| sind00280 Valine, leucine and isoleucine degradation | 5592_0.path0_m.933 | 354 | Dihydrolipoyl dehydrogenase 1, mitochondrial |
| sind00280 Valine, leucine and isoleucine degradation | 5592_0.path0_m.933 | 133 | Dihydrolipoyl dehydrogenase 1, mitochondrial |
| sind00195 Photosynthesis | 2769_0.path0_m.5164 | 466 | ATP synthase subunit alpha, chloroplastic |
| sind00195 Photosynthesis | 2769_0.path0_m.5164 | 114 | ATP synthase subunit alpha, chloroplastic |
| sind00195 Photosynthesis | 9127_0.path0_m.5312 | 99 | Photosystem I reaction center subunit IV, chloroplastic |
| sind00195 Photosynthesis | 7619_0.path0_m.3408 | 231 | Oxygen-evolving enhancer protein 2-1, chloroplastic |
| sind00195 Photosynthesis | 7619_0.path0_m.3408 | 112 | Oxygen-evolving enhancer protein 2-1, chloroplastic |
| sind00195 Photosynthesis | 7809_0.path0_m.3670 | 147 | Cytochrome b6-f complex iron-sulfur subunit, chloroplastic |
| sind00195 Photosynthesis | 7809_0.path0_m.3670 | 133 | Cytochrome b6-f complex iron-sulfur subunit, chloroplastic |
| sind00195 Photosynthesis | 8810_0.path5_m.5063 | 147 | Photosystem I reaction center subunit II, chloroplastic |
| sind00195 Photosynthesis | 8810_0.path5_m.5063 | 95 | Photosystem I reaction center subunit II, chloroplastic |
| sind00195 Photosynthesis | 8810_0.path5_m.5063 | 139 | Photosystem I reaction center subunit II, chloroplastic |
| sind00195 Photosynthesis | 8472_0.path0_m.4524 | 137 | Photosystem I reaction center subunit VI, chloroplastic |
| sind00195 Photosynthesis | 8472_0.path0_m.4524 | 98 | Photosystem I reaction center subunit VI, chloroplastic |
| sind00195 Photosynthesis | 7914_0.path0_m.3834 | 127 | Photosystem I reaction center subunit III, chloroplastic |
| sind00195 Photosynthesis | 7914_0.path0_m.3834 | 117 | Photosystem I reaction center subunit III, chloroplastic |
| sind00195 Photosynthesis | 7914_0.path0_m.3834 | 96 | Photosystem I reaction center subunit III, chloroplastic |
| sind00195 Photosynthesis | i0_LQ_c128861_f1p0_857_m.7234 | 155 | Oxygen-evolving enhancer protein 3-1, chloroplastic |
| sind00195 Photosynthesis | i0_LQ_c128861_f1p0_857_m.7234 | 129 | Oxygen-evolving enhancer protein 3-1, chloroplastic |
| sind00195 Photosynthesis | i0_LQ_c128861_f1p0_857_m.7234 | 115 | Oxygen-evolving enhancer protein 3-1, chloroplastic |
| sind00195 Photosynthesis | i0_LQ_c128861_f1p0_857_m.7234 | 166 | Oxygen-evolving enhancer protein 3-1, chloroplastic |
| sind00195 Photosynthesis | i0_LQ_c128861_f1p0_857_m.7234 | 132 | Oxygen-evolving enhancer protein 3-1, chloroplastic |
| sind00195 Photosynthesis | i0_LQ_c128861_f1p0_857_m.7234 | 76 | Oxygen-evolving enhancer protein 3-1, chloroplastic |
| sind00195 Photosynthesis | i0_LQ_c128861_f1p0_857_m.7234 | 117 | Oxygen-evolving enhancer protein 3-1, chloroplastic |
| sind00195 Photosynthesis | i0_LQ_c128861_f1p0_857_m.7234 | 82 | Oxygen-evolving enhancer protein 3-1, chloroplastic |
| sind00195 Photosynthesis | 6706_0.path0_m.2288 | 180 | Cytochrome f |
| sind00195 Photosynthesis | 6706_0.path0_m.2288 | 174 | Cytochrome f |
| sind00195 Photosynthesis | 6706_0.path0_m.2288 | 216 | Cytochrome f |
| sind00195 Photosynthesis | 5136_0.path0_m.111 | 308 | Photosystem II CP47 reaction center protein |
| sind00195 Photosynthesis | 5136_0.path0_m.111 | 304 | Photosystem II CP47 reaction center protein |
| sind00195 Photosynthesis | 5136_0.path0_m.111 | 438 | Photosystem II CP47 reaction center protein |
| sind00195 Photosynthesis | 5071_0.path1_m.2 | 68 | Photosystem I P700 chlorophyll a apoprotein A2 |
| sind00195 Photosynthesis | 6598_0.path0_m.2118 | 181 | Oxygen-evolving enhancer protein 1, chloroplastic |
| sind00195 Photosynthesis | 6598_0.path0_m.2118 | 136 | Oxygen-evolving enhancer protein 1, chloroplastic |
| sind00195 Photosynthesis | 6598_0.path0_m.2118 | 90 | Oxygen-evolving enhancer protein 1, chloroplastic |
| sind00195 Photosynthesis | 6598_0.path0_m.2118 | 120 | Oxygen-evolving enhancer protein 1, chloroplastic |
| sind00195 Photosynthesis | 6598_0.path0_m.2118 | 125 | Oxygen-evolving enhancer protein 1, chloroplastic |
| sind00195 Photosynthesis | 6598_0.path0_m.2118 | 213 | Oxygen-evolving enhancer protein 1, chloroplastic |
| sind00195 Photosynthesis | 6598_0.path0_m.2118 | 113 | Oxygen-evolving enhancer protein 1, chloroplastic |
| sind00195 Photosynthesis | i0_HQ_c28425_f2p22_869_m.4307 | 97 | Plastocyanin, chloroplastic |
| sind00195 Photosynthesis | 9654_0.path0_m.6072 | 111 | Photosystem I reaction center subunit V, chloroplastic |
| sind00195 Photosynthesis | i0_LQ_c102321_f1p1_510_m.5175 | 51 | Photosystem II 10 kDa polypeptide, chloroplastic |
| sind00940 Phenylpropanoid biosynthesis | 8418_0.path0_m.4439 | 115 | Peroxidase 4 |
| sind00940 Phenylpropanoid biosynthesis | 5201_0.path1_m.207 | 386 | Raucaffricine-O-beta-D-glucosidase |
| sind00940 Phenylpropanoid biosynthesis | 6115_0.path0_m.1574 | 266 | Beta-glucosidase 12 |
| sind00940 Phenylpropanoid biosynthesis | 6115_0.path0_m.1574 | 113 | Beta-glucosidase 12 |
| sind01110 Biosynthesis of secondary metabolites | 5856_0.path0_m.1092 | 163 | Succinate--CoA ligase [ADP-forming] subunit beta, mitochondrial |
| sind01110 Biosynthesis of secondary metabolites | 5856_0.path0_m.1092 | 119 | Succinate--CoA ligase [ADP-forming] subunit beta, mitochondrial |
| sind01110 Biosynthesis of secondary metabolites | 5856_0.path0_m.1092 | 205 | Succinate--CoA ligase [ADP-forming] subunit beta, mitochondrial |
| sind01110 Biosynthesis of secondary metabolites | 5856_0.path0_m.1092 | 174 | Succinate--CoA ligase [ADP-forming] subunit beta, mitochondrial |
| sind01110 Biosynthesis of secondary metabolites | 5856_0.path0_m.1092 | 89 | Succinate--CoA ligase [ADP-forming] subunit beta, mitochondrial |
| sind01110 Biosynthesis of secondary metabolites | 7493_0.path1_m.3233 | 197 | Probable NAD(P)H dehydrogenase (quinone) FQR1-like 1 |
| sind01110 Biosynthesis of secondary metabolites | i1_HQ_c14606_f2p0_1675_m.3241 | 179 | Aspartate aminotransferase, mitochondrial |
| sind01110 Biosynthesis of secondary metabolites | i1_HQ_c14606_f2p0_1675_m.3241 | 374 | Aspartate aminotransferase, mitochondrial |
| sind01110 Biosynthesis of secondary metabolites | 1931_0.path2_m.3930 | 373 | 2-oxoglutarate dehydrogenase, mitochondrial |
| sind01110 Biosynthesis of secondary metabolites | 1931_0.path2_m.3930 | 437 | 2-oxoglutarate dehydrogenase, mitochondrial |
| sind01110 Biosynthesis of secondary metabolites | 1931_0.path2_m.3930 | 446 | 2-oxoglutarate dehydrogenase, mitochondrial |
| sind01110 Biosynthesis of secondary metabolites | 1931_0.path2_m.3930 | 218 | 2-oxoglutarate dehydrogenase, mitochondrial |
| sind01110 Biosynthesis of secondary metabolites | 1931_0.path2_m.3930 | 392 | 2-oxoglutarate dehydrogenase, mitochondrial |
| sind01110 Biosynthesis of secondary metabolites | 1931_0.path2_m.3930 | 362 | 2-oxoglutarate dehydrogenase, mitochondrial |
| sind01110 Biosynthesis of secondary metabolites | 1931_0.path2_m.3930 | 459 | 2-oxoglutarate dehydrogenase, mitochondrial |
| sind01110 Biosynthesis of secondary metabolites | 1931_0.path2_m.3930 | 843 | 2-oxoglutarate dehydrogenase, mitochondrial |
| sind01110 Biosynthesis of secondary metabolites | 5201_0.path1_m.207 | 386 | Raucaffricine-O-beta-D-glucosidase |
| sind01110 Biosynthesis of secondary metabolites | i1_LQ_c7379_f1p0_1442_m.2478 | 292 | 1-aminocyclopropane-1-carboxylate oxidase 3 |
| sind01110 Biosynthesis of secondary metabolites | 2789_0.path4_m.5221 | 83 | Aconitate hydratase 2, mitochondrial |
| sind01110 Biosynthesis of secondary metabolites | 5737_0.path0_m.1203 | 149 | Polyphenol oxidase I, chloroplastic |
| sind01110 Biosynthesis of secondary metabolites | 5737_0.path0_m.1203 | 572 | Polyphenol oxidase I, chloroplastic |
| sind01110 Biosynthesis of secondary metabolites | 5737_0.path0_m.1203 | 562 | Polyphenol oxidase I, chloroplastic |
| sind01110 Biosynthesis of secondary metabolites | 5737_0.path0_m.1203 | 524 | Polyphenol oxidase I, chloroplastic |
| sind01110 Biosynthesis of secondary metabolites | 5737_0.path0_m.1203 | 134 | Polyphenol oxidase I, chloroplastic |
| sind01110 Biosynthesis of secondary metabolites | 5737_0.path0_m.1203 | 484 | Polyphenol oxidase I, chloroplastic |
| sind01110 Biosynthesis of secondary metabolites | 5737_0.path0_m.1203 | 305 | Polyphenol oxidase I, chloroplastic |
| sind01110 Biosynthesis of secondary metabolites | 5737_0.path0_m.1203 | 511 | Polyphenol oxidase I, chloroplastic |
| sind01110 Biosynthesis of secondary metabolites | 5737_0.path0_m.1203 | 491 | Polyphenol oxidase I, chloroplastic |
| sind01110 Biosynthesis of secondary metabolites | 5737_0.path0_m.1203 | 126 | Polyphenol oxidase I, chloroplastic |
| sind01110 Biosynthesis of secondary metabolites | 5532_0.path0_m.831 | 22 | Polyphenol oxidase I, chloroplastic |
| sind01110 Biosynthesis of secondary metabolites | 9666_0.path0_m.6084 | 157 | Dihydrolipoyllysine-residue acetyltransferase component 3 of pyruvate dehydrogenase complex, mitochondrial |
| sind01110 Biosynthesis of secondary metabolites | 9666_0.path0_m.6084 | 150 | Dihydrolipoyllysine-residue acetyltransferase component 3 of pyruvate dehydrogenase complex, mitochondrial |
| sind01110 Biosynthesis of secondary metabolites | i1_HQ_c32623_f2p0_1256_m.6619 | 79 | Succinate--CoA ligase [ADP-forming] subunit alpha-2, mitochondrial |
| sind01110 Biosynthesis of secondary metabolites | 2221_0.path0_m.4534 | 604 | 2-oxoglutarate dehydrogenase, mitochondrial |
| sind01110 Biosynthesis of secondary metabolites | 5592_0.path0_m.933 | 306 | Dihydrolipoyl dehydrogenase 1, mitochondrial |
| sind01110 Biosynthesis of secondary metabolites | 5592_0.path0_m.933 | 181 | Dihydrolipoyl dehydrogenase 1, mitochondrial |
| sind01110 Biosynthesis of secondary metabolites | 5592_0.path0_m.933 | 207 | Dihydrolipoyl dehydrogenase 1, mitochondrial |
| sind01110 Biosynthesis of secondary metabolites | 5592_0.path0_m.933 | 97 | Dihydrolipoyl dehydrogenase 1, mitochondrial |
| sind01110 Biosynthesis of secondary metabolites | 5592_0.path0_m.933 | 218 | Dihydrolipoyl dehydrogenase 1, mitochondrial |
| sind01110 Biosynthesis of secondary metabolites | 5592_0.path0_m.933 | 176 | Dihydrolipoyl dehydrogenase 1, mitochondrial |
| sind01110 Biosynthesis of secondary metabolites | 5592_0.path0_m.933 | 448 | Dihydrolipoyl dehydrogenase 1, mitochondrial |
| sind01110 Biosynthesis of secondary metabolites | 5592_0.path0_m.933 | 205 | Dihydrolipoyl dehydrogenase 1, mitochondrial |
| sind01110 Biosynthesis of secondary metabolites | 5592_0.path0_m.933 | 166 | Dihydrolipoyl dehydrogenase 1, mitochondrial |
| sind01110 Biosynthesis of secondary metabolites | 5592_0.path0_m.933 | 173 | Dihydrolipoyl dehydrogenase 1, mitochondrial |
| sind01110 Biosynthesis of secondary metabolites | 5592_0.path0_m.933 | 354 | Dihydrolipoyl dehydrogenase 1, mitochondrial |
| sind01110 Biosynthesis of secondary metabolites | 5592_0.path0_m.933 | 133 | Dihydrolipoyl dehydrogenase 1, mitochondrial |
| sind01110 Biosynthesis of secondary metabolites | i0_LQ_c35465_f1p1_640_m.6645 | 115 | Aminomethyltransferase, mitochondrial |
| sind01110 Biosynthesis of secondary metabolites | 4933_0.path0_m.8308 | 249 | Polyphenol oxidase II, chloroplastic |
| sind01110 Biosynthesis of secondary metabolites | 4933_0.path0_m.8308 | 262 | Polyphenol oxidase II, chloroplastic |
| sind01110 Biosynthesis of secondary metabolites | 4933_0.path0_m.8308 | 460 | Polyphenol oxidase II, chloroplastic |
| sind01110 Biosynthesis of secondary metabolites | 4933_0.path0_m.8308 | 137 | Polyphenol oxidase II, chloroplastic |
| sind01110 Biosynthesis of secondary metabolites | 4933_0.path0_m.8308 | 447 | Polyphenol oxidase II, chloroplastic |
| sind01110 Biosynthesis of secondary metabolites | 4933_0.path0_m.8308 | 427 | Polyphenol oxidase II, chloroplastic |
| sind01110 Biosynthesis of secondary metabolites | 4933_0.path0_m.8308 | 453 | Polyphenol oxidase II, chloroplastic |
| sind01110 Biosynthesis of secondary metabolites | 4933_0.path0_m.8308 | 145 | Polyphenol oxidase II, chloroplastic |
| sind01110 Biosynthesis of secondary metabolites | 4933_0.path0_m.8308 | 419 | Polyphenol oxidase II, chloroplastic |
| sind01110 Biosynthesis of secondary metabolites | 4933_0.path0_m.8308 | 151 | Polyphenol oxidase II, chloroplastic |
| sind01110 Biosynthesis of secondary metabolites | 6655_0.path0_m.2208 | 77 | Succinate--CoA ligase [ADP-forming] subunit alpha-2, mitochondrial |
| sind01110 Biosynthesis of secondary metabolites | 4202_0.path1_m.7266 | 309 | Polyphenol oxidase II, chloroplastic |
| sind01110 Biosynthesis of secondary metabolites | 4202_0.path1_m.7266 | 528 | Polyphenol oxidase II, chloroplastic |
| sind01110 Biosynthesis of secondary metabolites | 4202_0.path1_m.7266 | 583 | Polyphenol oxidase II, chloroplastic |
| sind01110 Biosynthesis of secondary metabolites | 4202_0.path1_m.7266 | 442 | Polyphenol oxidase II, chloroplastic |
| sind01110 Biosynthesis of secondary metabolites | 4202_0.path1_m.7266 | 140 | Polyphenol oxidase II, chloroplastic |
| sind01110 Biosynthesis of secondary metabolites | 4202_0.path1_m.7266 | 576 | Polyphenol oxidase II, chloroplastic |
| sind01110 Biosynthesis of secondary metabolites | 6817_0.path0_m.2451 | 207 | Adenylate kinase 4 |
| sind01110 Biosynthesis of secondary metabolites | 6817_0.path0_m.2451 | 72 | Adenylate kinase 4 |
| sind01110 Biosynthesis of secondary metabolites | i1_LQ_c50855_f1p1_1111_m.2233 | 50 | Chlorophyllase-1 |
| sind01110 Biosynthesis of secondary metabolites | 7090_0.path1_m.2911 | 178 | Nucleoside diphosphate kinase IV, chloroplastic_mitochondrial |
| sind01110 Biosynthesis of secondary metabolites | 6115_0.path0_m.1574 | 266 | Beta-glucosidase 12 |
| sind01110 Biosynthesis of secondary metabolites | 6115_0.path0_m.1574 | 113 | Beta-glucosidase 12 |
| sind01110 Biosynthesis of secondary metabolites | 9636_0.path0_m.6039 | 76 | Pyruvate dehydrogenase E1 component subunit beta-1, mitochondrial |
| sind01110 Biosynthesis of secondary metabolites | 9685_0.path0_m.6107 | 401 | Serine hydroxymethyltransferase, mitochondrial |
| sind01110 Biosynthesis of secondary metabolites | 9685_0.path0_m.6107 | 412 | Serine hydroxymethyltransferase, mitochondrial |
| sind01110 Biosynthesis of secondary metabolites | 9685_0.path0_m.6107 | 185 | Serine hydroxymethyltransferase, mitochondrial |
| sind01110 Biosynthesis of secondary metabolites | 9685_0.path0_m.6107 | 471 | Serine hydroxymethyltransferase, mitochondrial |
| sind01110 Biosynthesis of secondary metabolites | 9685_0.path0_m.6107 | 507 | Serine hydroxymethyltransferase, mitochondrial |
| sind01110 Biosynthesis of secondary metabolites | 8418_0.path0_m.4439 | 115 | Peroxidase 4 |
| sind01110 Biosynthesis of secondary metabolites | 9257_0.path1_m.5500 | 401 | Citrate synthase, mitochondrial |
| sind01110 Biosynthesis of secondary metabolites | 9257_0.path1_m.5500 | 346 | Citrate synthase, mitochondrial |
| sind01110 Biosynthesis of secondary metabolites | 5891_0.path0_m.1161 | 266 | ATP-citrate synthase beta chain protein 2 |
| sind01110 Biosynthesis of secondary metabolites | 5250_0.path0_m.317 | 709 | Glyoxysomal fatty acid beta-oxidation multifunctional protein MFP-a |
| sind01110 Biosynthesis of secondary metabolites | 5250_0.path0_m.317 | 374 | Glyoxysomal fatty acid beta-oxidation multifunctional protein MFP-a |
| sind01110 Biosynthesis of secondary metabolites | 5250_0.path0_m.317 | 355 | Glyoxysomal fatty acid beta-oxidation multifunctional protein MFP-a |
| sind01110 Biosynthesis of secondary metabolites | i1_LQ_c26180_f2p0_1342_m.6236 | 203 | Dihydrolipoyllysine-residue acetyltransferase component 1 of pyruvate dehydrogenase complex, mitochondrial |
| sind01110 Biosynthesis of secondary metabolites | 4658_0.path0_m.7768 | 148 | Phosphoglycerate kinase, chloroplastic |
| sind01110 Biosynthesis of secondary metabolites | 4897_0.path0_m.8229 | 164 | Succinate dehydrogenase [ubiquinone] flavoprotein subunit 1, mitochondrial |
| sind01110 Biosynthesis of secondary metabolites | 4897_0.path0_m.8229 | 318 | Succinate dehydrogenase [ubiquinone] flavoprotein subunit 1, mitochondrial |
| sind01110 Biosynthesis of secondary metabolites | 9209_0.path0_m.5429 | 228 | Citrate synthase, glyoxysomal |
| sind01110 Biosynthesis of secondary metabolites | 3539_0.path0_m.6388 | 867 | Glycine dehydrogenase (decarboxylating), mitochondrial |
| sind01110 Biosynthesis of secondary metabolites | 3539_0.path0_m.6388 | 700 | Glycine dehydrogenase (decarboxylating), mitochondrial |
| sind01110 Biosynthesis of secondary metabolites | i0_LQ_c104330_f1p1_837_m.4398 | 221 | Peroxisomal (S)-2-hydroxy-acid oxidase GLO2 |
| sind01110 Biosynthesis of secondary metabolites | 4752_1.path0_m.7937 | 124 | Dihydrolipoyllysine-residue succinyltransferase component of 2-oxoglutarate dehydrogenase complex 2, mitochondrial |
| sind01110 Biosynthesis of secondary metabolites | 4752_1.path0_m.7937 | 322 | Dihydrolipoyllysine-residue succinyltransferase component of 2-oxoglutarate dehydrogenase complex 2, mitochondrial |
| sind01110 Biosynthesis of secondary metabolites | 4752_1.path0_m.7937 | 249 | Dihydrolipoyllysine-residue succinyltransferase component of 2-oxoglutarate dehydrogenase complex 2, mitochondrial |
| sind01110 Biosynthesis of secondary metabolites | 8601_0.path0_m.4749 | 205 | Isocitrate dehydrogenase [NAD] catalytic subunit 5, mitochondrial |
| sind01110 Biosynthesis of secondary metabolites | 8601_0.path0_m.4749 | 332 | Isocitrate dehydrogenase [NAD] catalytic subunit 5, mitochondrial |
| sind01110 Biosynthesis of secondary metabolites | 8601_0.path0_m.4749 | 168 | Isocitrate dehydrogenase [NAD] catalytic subunit 5, mitochondrial |
| sind01110 Biosynthesis of secondary metabolites | 6944_0.path0_m.2670 | 16 | ATP-citrate synthase alpha chain protein 2 |
| sind01110 Biosynthesis of secondary metabolites | 6944_0.path0_m.2670 | 58 | ATP-citrate synthase alpha chain protein 2 |
| sind01110 Biosynthesis of secondary metabolites | 1242_0.path10_m.2975 | 613 | 2-oxoglutarate dehydrogenase, mitochondrial |
| sind01110 Biosynthesis of secondary metabolites | 1242_0.path10_m.2975 | 603 | 2-oxoglutarate dehydrogenase, mitochondrial |
| sind01110 Biosynthesis of secondary metabolites | i1_LQ_c90359_f1p0_1008_m.5193 | 216 | Probable NAD(P)H dehydrogenase (quinone) FQR1-like 1 |
| sind01110 Biosynthesis of secondary metabolites | i1_LQ_c90359_f1p0_1008_m.5193 | 223 | Probable NAD(P)H dehydrogenase (quinone) FQR1-like 1 |
| sind01110 Biosynthesis of secondary metabolites | 5827_0.path3_m.1058 | 151 | Peroxisomal (S)-2-hydroxy-acid oxidase GLO1 |
| sind01110 Biosynthesis of secondary metabolites | i1_LQ_c77746_f1p0_1530_m.131 | 223 | Probable acetyl-CoA acetyltransferase, cytosolic 2 |
| sind01110 Biosynthesis of secondary metabolites | i1_HQ_c41079_f2p0_1865_m.5127 | 54 | Glutamate--glyoxylate aminotransferase 2 |
| sind01110 Biosynthesis of secondary metabolites | 1589_0.path0_m.3099 | 128 | Aconitate hydratase 2, mitochondrial |
| sind01110 Biosynthesis of secondary metabolites | 1589_0.path0_m.3099 | 122 | Aconitate hydratase 2, mitochondrial |
| sind01110 Biosynthesis of secondary metabolites | 1589_0.path0_m.3099 | 558 | Aconitate hydratase 2, mitochondrial |
| sind01110 Biosynthesis of secondary metabolites | 1589_0.path0_m.3099 | 381 | Aconitate hydratase 2, mitochondrial |
| sind01110 Biosynthesis of secondary metabolites | 1589_0.path0_m.3099 | 114 | Aconitate hydratase 2, mitochondrial |
| sind01110 Biosynthesis of secondary metabolites | 1589_0.path0_m.3099 | 166 | Aconitate hydratase 2, mitochondrial |
| sind01110 Biosynthesis of secondary metabolites | 1589_0.path0_m.3099 | 844 | Aconitate hydratase 2, mitochondrial |
| sind01110 Biosynthesis of secondary metabolites | 1589_0.path0_m.3099 | 855 | Aconitate hydratase 2, mitochondrial |
| sind01110 Biosynthesis of secondary metabolites | 1589_0.path0_m.3099 | 508 | Aconitate hydratase 2, mitochondrial |
| sind01110 Biosynthesis of secondary metabolites | 1589_0.path0_m.3099 | 574 | Aconitate hydratase 2, mitochondrial |
| sind01110 Biosynthesis of secondary metabolites | i1_HQ_c60950_f14p0_1160_m.1131 | 220 | Cysteine synthase |
| sind01110 Biosynthesis of secondary metabolites | 11093_0.path0_m.1258 | 154 | Glycine cleavage system H protein 3, mitochondrial |
| sind01110 Biosynthesis of secondary metabolites | 11093_0.path0_m.1258 | 140 | Glycine cleavage system H protein 3, mitochondrial |
| sind01110 Biosynthesis of secondary metabolites | 11093_0.path0_m.1258 | 47 | Glycine cleavage system H protein 3, mitochondrial |
| sind01110 Biosynthesis of secondary metabolites | 6551_0.path0_m.2298 | 59 | Fatty acid hydroperoxide lyase, chloroplastic |
| sind01110 Biosynthesis of secondary metabolites | 7112_0.path0_m.2939 | 371 | Pyruvate dehydrogenase E1 component subunit alpha, mitochondrial |
| sind01110 Biosynthesis of secondary metabolites | 7112_0.path0_m.2939 | 333 | Pyruvate dehydrogenase E1 component subunit alpha, mitochondrial |
| sind01110 Biosynthesis of secondary metabolites | 7112_0.path0_m.2939 | 354 | Pyruvate dehydrogenase E1 component subunit alpha, mitochondrial |
| sind01110 Biosynthesis of secondary metabolites | 7112_0.path0_m.2939 | 239 | Pyruvate dehydrogenase E1 component subunit alpha, mitochondrial |
| sind01110 Biosynthesis of secondary metabolites | 7112_0.path0_m.2939 | 322 | Pyruvate dehydrogenase E1 component subunit alpha, mitochondrial |
| sind01110 Biosynthesis of secondary metabolites | 10152_0.path0_m.229 | 369 | Fructose-bisphosphate aldolase 2, chloroplastic |
| sind01110 Biosynthesis of secondary metabolites | 10152_0.path0_m.229 | 61 | Fructose-bisphosphate aldolase 2, chloroplastic |
| sind01110 Biosynthesis of secondary metabolites | i1_LQ_c67740_f1p0_1601_m.6729 | 74 | Phosphoglycerate kinase, cytosolic |
| sind01110 Biosynthesis of secondary metabolites | 11884_0.path0_m.2391 | 40 | Fructose-bisphosphate aldolase 6, cytosolic |
| sind01110 Biosynthesis of secondary metabolites | 11884_0.path0_m.2391 | 357 | Fructose-bisphosphate aldolase 6, cytosolic |
| sind00220 Arginine biosynthesis | i1_HQ_c41079_f2p0_1865_m.5127 | 54 | Glutamate--glyoxylate aminotransferase 2 |
| sind00220 Arginine biosynthesis | 9538_0.path0_m.5901 | 354 | Glutamine synthetase, chloroplastic |
| sind00220 Arginine biosynthesis | 9538_0.path0_m.5901 | 101 | Glutamine synthetase, chloroplastic |
| sind00220 Arginine biosynthesis | 9538_0.path0_m.5901 | 168 | Glutamine synthetase, chloroplastic |
| sind00220 Arginine biosynthesis | 6440_0.path0_m.2112 | 312 | Glutamine synthetase cytosolic isozyme |
| sind00220 Arginine biosynthesis | 6440_0.path0_m.2112 | 289 | Glutamine synthetase cytosolic isozyme |
| sind00220 Arginine biosynthesis | 6440_0.path0_m.2112 | 322 | Glutamine synthetase cytosolic isozyme |
| sind00220 Arginine biosynthesis | i1_HQ_c14606_f2p0_1675_m.3241 | 179 | Aspartate aminotransferase, mitochondrial |
| sind00220 Arginine biosynthesis | i1_HQ_c14606_f2p0_1675_m.3241 | 374 | Aspartate aminotransferase, mitochondrial |
| sind00220 Arginine biosynthesis | 11907_0.path0_m.2413 | 311 | Glutamine synthetase cytosolic isozyme |
| sind01200 Carbon metabolism | 5856_0.path0_m.1092 | 163 | Succinate--CoA ligase [ADP-forming] subunit beta, mitochondrial |
| sind01200 Carbon metabolism | 5856_0.path0_m.1092 | 119 | Succinate--CoA ligase [ADP-forming] subunit beta, mitochondrial |
| sind01200 Carbon metabolism | 5856_0.path0_m.1092 | 205 | Succinate--CoA ligase [ADP-forming] subunit beta, mitochondrial |
| sind01200 Carbon metabolism | 5856_0.path0_m.1092 | 174 | Succinate--CoA ligase [ADP-forming] subunit beta, mitochondrial |
| sind01200 Carbon metabolism | 5856_0.path0_m.1092 | 89 | Succinate--CoA ligase [ADP-forming] subunit beta, mitochondrial |
| sind01200 Carbon metabolism | i1_HQ_c14606_f2p0_1675_m.3241 | 179 | Aspartate aminotransferase, mitochondrial |
| sind01200 Carbon metabolism | i1_HQ_c14606_f2p0_1675_m.3241 | 374 | Aspartate aminotransferase, mitochondrial |
| sind01200 Carbon metabolism | 1931_0.path2_m.3930 | 373 | 2-oxoglutarate dehydrogenase, mitochondrial |
| sind01200 Carbon metabolism | 1931_0.path2_m.3930 | 437 | 2-oxoglutarate dehydrogenase, mitochondrial |
| sind01200 Carbon metabolism | 1931_0.path2_m.3930 | 446 | 2-oxoglutarate dehydrogenase, mitochondrial |
| sind01200 Carbon metabolism | 1931_0.path2_m.3930 | 218 | 2-oxoglutarate dehydrogenase, mitochondrial |
| sind01200 Carbon metabolism | 1931_0.path2_m.3930 | 392 | 2-oxoglutarate dehydrogenase, mitochondrial |
| sind01200 Carbon metabolism | 1931_0.path2_m.3930 | 362 | 2-oxoglutarate dehydrogenase, mitochondrial |
| sind01200 Carbon metabolism | 1931_0.path2_m.3930 | 459 | 2-oxoglutarate dehydrogenase, mitochondrial |
| sind01200 Carbon metabolism | 1931_0.path2_m.3930 | 843 | 2-oxoglutarate dehydrogenase, mitochondrial |
| sind01200 Carbon metabolism | 2789_0.path4_m.5221 | 83 | Aconitate hydratase 2, mitochondrial |
| sind01200 Carbon metabolism | 9666_0.path0_m.6084 | 157 | Dihydrolipoyllysine-residue acetyltransferase component 3 of pyruvate dehydrogenase complex, mitochondrial |
| sind01200 Carbon metabolism | 9666_0.path0_m.6084 | 150 | Dihydrolipoyllysine-residue acetyltransferase component 3 of pyruvate dehydrogenase complex, mitochondrial |
| sind01200 Carbon metabolism | i1_HQ_c32623_f2p0_1256_m.6619 | 79 | Succinate--CoA ligase [ADP-forming] subunit alpha-2, mitochondrial |
| sind01200 Carbon metabolism | 8673_0.path2_m.4855 | 11 | Ribulose bisphosphate carboxylase small chain, chloroplastic |
| sind01200 Carbon metabolism | 8673_0.path2_m.4855 | 107 | Ribulose bisphosphate carboxylase small chain, chloroplastic |
| sind01200 Carbon metabolism | 2221_0.path0_m.4534 | 604 | 2-oxoglutarate dehydrogenase, mitochondrial |
| sind01200 Carbon metabolism | 5592_0.path0_m.933 | 306 | Dihydrolipoyl dehydrogenase 1, mitochondrial |
| sind01200 Carbon metabolism | 5592_0.path0_m.933 | 181 | Dihydrolipoyl dehydrogenase 1, mitochondrial |
| sind01200 Carbon metabolism | 5592_0.path0_m.933 | 207 | Dihydrolipoyl dehydrogenase 1, mitochondrial |
| sind01200 Carbon metabolism | 5592_0.path0_m.933 | 97 | Dihydrolipoyl dehydrogenase 1, mitochondrial |
| sind01200 Carbon metabolism | 5592_0.path0_m.933 | 218 | Dihydrolipoyl dehydrogenase 1, mitochondrial |
| sind01200 Carbon metabolism | 5592_0.path0_m.933 | 176 | Dihydrolipoyl dehydrogenase 1, mitochondrial |
| sind01200 Carbon metabolism | 5592_0.path0_m.933 | 448 | Dihydrolipoyl dehydrogenase 1, mitochondrial |
| sind01200 Carbon metabolism | 5592_0.path0_m.933 | 205 | Dihydrolipoyl dehydrogenase 1, mitochondrial |
| sind01200 Carbon metabolism | 5592_0.path0_m.933 | 166 | Dihydrolipoyl dehydrogenase 1, mitochondrial |
| sind01200 Carbon metabolism | 5592_0.path0_m.933 | 173 | Dihydrolipoyl dehydrogenase 1, mitochondrial |
| sind01200 Carbon metabolism | 5592_0.path0_m.933 | 354 | Dihydrolipoyl dehydrogenase 1, mitochondrial |
| sind01200 Carbon metabolism | 5592_0.path0_m.933 | 133 | Dihydrolipoyl dehydrogenase 1, mitochondrial |
| sind01200 Carbon metabolism | i0_LQ_c35465_f1p1_640_m.6645 | 115 | Aminomethyltransferase, mitochondrial |
| sind01200 Carbon metabolism | 6655_0.path0_m.2208 | 77 | Succinate--CoA ligase [ADP-forming] subunit alpha-2, mitochondrial |
| sind01200 Carbon metabolism | 5638_0.path0_m.1009 | 190 | Ribulose bisphosphate carboxylase large chain |
| sind01200 Carbon metabolism | 5638_0.path0_m.1009 | 182 | Ribulose bisphosphate carboxylase large chain |
| sind01200 Carbon metabolism | 5638_0.path0_m.1009 | 39 | Ribulose bisphosphate carboxylase large chain |
| sind01200 Carbon metabolism | 5638_0.path0_m.1009 | 341 | Ribulose bisphosphate carboxylase large chain |
| sind01200 Carbon metabolism | 5638_0.path0_m.1009 | 153 | Ribulose bisphosphate carboxylase large chain |
| sind01200 Carbon metabolism | 9636_0.path0_m.6039 | 76 | Pyruvate dehydrogenase E1 component subunit beta-1, mitochondrial |
| sind01200 Carbon metabolism | 9685_0.path0_m.6107 | 401 | Serine hydroxymethyltransferase, mitochondrial |
| sind01200 Carbon metabolism | 9685_0.path0_m.6107 | 412 | Serine hydroxymethyltransferase, mitochondrial |
| sind01200 Carbon metabolism | 9685_0.path0_m.6107 | 185 | Serine hydroxymethyltransferase, mitochondrial |
| sind01200 Carbon metabolism | 9685_0.path0_m.6107 | 471 | Serine hydroxymethyltransferase, mitochondrial |
| sind01200 Carbon metabolism | 9685_0.path0_m.6107 | 507 | Serine hydroxymethyltransferase, mitochondrial |
| sind01200 Carbon metabolism | 9257_0.path1_m.5500 | 401 | Citrate synthase, mitochondrial |
| sind01200 Carbon metabolism | 9257_0.path1_m.5500 | 346 | Citrate synthase, mitochondrial |
| sind01200 Carbon metabolism | i1_LQ_c26180_f2p0_1342_m.6236 | 203 | Dihydrolipoyllysine-residue acetyltransferase component 1 of pyruvate dehydrogenase complex, mitochondrial |
| sind01200 Carbon metabolism | 4658_0.path0_m.7768 | 148 | Phosphoglycerate kinase, chloroplastic |
| sind01200 Carbon metabolism | 4897_0.path0_m.8229 | 164 | Succinate dehydrogenase [ubiquinone] flavoprotein subunit 1, mitochondrial |
| sind01200 Carbon metabolism | 4897_0.path0_m.8229 | 318 | Succinate dehydrogenase [ubiquinone] flavoprotein subunit 1, mitochondrial |
| sind01200 Carbon metabolism | 9209_0.path0_m.5429 | 228 | Citrate synthase, glyoxysomal |
| sind01200 Carbon metabolism | 3539_0.path0_m.6388 | 867 | Glycine dehydrogenase (decarboxylating), mitochondrial |
| sind01200 Carbon metabolism | 3539_0.path0_m.6388 | 700 | Glycine dehydrogenase (decarboxylating), mitochondrial |
| sind01200 Carbon metabolism | i0_LQ_c104330_f1p1_837_m.4398 | 221 | Peroxisomal (S)-2-hydroxy-acid oxidase GLO2 |
| sind01200 Carbon metabolism | 8394_0.path0_m.4400 | 119 | Glyceraldehyde-3-phosphate dehydrogenase B, chloroplastic |
| sind01200 Carbon metabolism | 8394_0.path0_m.4400 | 271 | Glyceraldehyde-3-phosphate dehydrogenase B, chloroplastic |
| sind01200 Carbon metabolism | 8394_0.path0_m.4400 | 91 | Glyceraldehyde-3-phosphate dehydrogenase B, chloroplastic |
| sind01200 Carbon metabolism | 4752_1.path0_m.7937 | 124 | Dihydrolipoyllysine-residue succinyltransferase component of 2-oxoglutarate dehydrogenase complex 2, mitochondrial |
| sind01200 Carbon metabolism | 4752_1.path0_m.7937 | 322 | Dihydrolipoyllysine-residue succinyltransferase component of 2-oxoglutarate dehydrogenase complex 2, mitochondrial |
| sind01200 Carbon metabolism | 4752_1.path0_m.7937 | 249 | Dihydrolipoyllysine-residue succinyltransferase component of 2-oxoglutarate dehydrogenase complex 2, mitochondrial |
| sind01200 Carbon metabolism | 8601_0.path0_m.4749 | 205 | Isocitrate dehydrogenase [NAD] catalytic subunit 5, mitochondrial |
| sind01200 Carbon metabolism | 8601_0.path0_m.4749 | 332 | Isocitrate dehydrogenase [NAD] catalytic subunit 5, mitochondrial |
| sind01200 Carbon metabolism | 8601_0.path0_m.4749 | 168 | Isocitrate dehydrogenase [NAD] catalytic subunit 5, mitochondrial |
| sind01200 Carbon metabolism | 11384_0.path0_m.1588 | 308 | Sedoheptulose-1,7-bisphosphatase, chloroplastic |
| sind01200 Carbon metabolism | i1_LQ_c14245_f1p3_1457_m.1128 | 279 | 3-hydroxyisobutyryl-CoA hydrolase-like protein 3, mitochondrial |
| sind01200 Carbon metabolism | 1242_0.path10_m.2975 | 613 | 2-oxoglutarate dehydrogenase, mitochondrial |
| sind01200 Carbon metabolism | 1242_0.path10_m.2975 | 603 | 2-oxoglutarate dehydrogenase, mitochondrial |
| sind01200 Carbon metabolism | 10683_0.path0_m.953 | 261 | Formate dehydrogenase, mitochondrial |
| sind01200 Carbon metabolism | 5827_0.path3_m.1058 | 151 | Peroxisomal (S)-2-hydroxy-acid oxidase GLO1 |
| sind01200 Carbon metabolism | i1_LQ_c77746_f1p0_1530_m.131 | 223 | Probable acetyl-CoA acetyltransferase, cytosolic 2 |
| sind01200 Carbon metabolism | i1_HQ_c41079_f2p0_1865_m.5127 | 54 | Glutamate--glyoxylate aminotransferase 2 |
| sind01200 Carbon metabolism | 3773_0.path1_m.6408 | 229 | NAD-dependent malic enzyme 59 kDa isoform, mitochondrial |
| sind01200 Carbon metabolism | 1589_0.path0_m.3099 | 128 | Aconitate hydratase 2, mitochondrial |
| sind01200 Carbon metabolism | 1589_0.path0_m.3099 | 122 | Aconitate hydratase 2, mitochondrial |
| sind01200 Carbon metabolism | 1589_0.path0_m.3099 | 558 | Aconitate hydratase 2, mitochondrial |
| sind01200 Carbon metabolism | 1589_0.path0_m.3099 | 381 | Aconitate hydratase 2, mitochondrial |
| sind01200 Carbon metabolism | 1589_0.path0_m.3099 | 114 | Aconitate hydratase 2, mitochondrial |
| sind01200 Carbon metabolism | 1589_0.path0_m.3099 | 166 | Aconitate hydratase 2, mitochondrial |
| sind01200 Carbon metabolism | 1589_0.path0_m.3099 | 844 | Aconitate hydratase 2, mitochondrial |
| sind01200 Carbon metabolism | 1589_0.path0_m.3099 | 855 | Aconitate hydratase 2, mitochondrial |
| sind01200 Carbon metabolism | 1589_0.path0_m.3099 | 508 | Aconitate hydratase 2, mitochondrial |
| sind01200 Carbon metabolism | 1589_0.path0_m.3099 | 574 | Aconitate hydratase 2, mitochondrial |
| sind01200 Carbon metabolism | i1_HQ_c60950_f14p0_1160_m.1131 | 220 | Cysteine synthase |
| sind01200 Carbon metabolism | 3879_0.path0_m.6640 | 99 | NAD-dependent malic enzyme 62 kDa isoform, mitochondrial |
| sind01200 Carbon metabolism | 3879_0.path0_m.6640 | 299 | NAD-dependent malic enzyme 62 kDa isoform, mitochondrial |
| sind01200 Carbon metabolism | 11093_0.path0_m.1258 | 154 | Glycine cleavage system H protein 3, mitochondrial |
| sind01200 Carbon metabolism | 11093_0.path0_m.1258 | 140 | Glycine cleavage system H protein 3, mitochondrial |
| sind01200 Carbon metabolism | 11093_0.path0_m.1258 | 47 | Glycine cleavage system H protein 3, mitochondrial |
| sind01200 Carbon metabolism | 7112_0.path0_m.2939 | 371 | Pyruvate dehydrogenase E1 component subunit alpha, mitochondrial |
| sind01200 Carbon metabolism | 7112_0.path0_m.2939 | 333 | Pyruvate dehydrogenase E1 component subunit alpha, mitochondrial |
| sind01200 Carbon metabolism | 7112_0.path0_m.2939 | 354 | Pyruvate dehydrogenase E1 component subunit alpha, mitochondrial |
| sind01200 Carbon metabolism | 7112_0.path0_m.2939 | 239 | Pyruvate dehydrogenase E1 component subunit alpha, mitochondrial |
| sind01200 Carbon metabolism | 7112_0.path0_m.2939 | 322 | Pyruvate dehydrogenase E1 component subunit alpha, mitochondrial |
| sind01200 Carbon metabolism | 4476_0.path1_m.7434 | 411 | NADP-dependent malic enzyme |
| sind01200 Carbon metabolism | 10152_0.path0_m.229 | 369 | Fructose-bisphosphate aldolase 2, chloroplastic |
| sind01200 Carbon metabolism | 10152_0.path0_m.229 | 61 | Fructose-bisphosphate aldolase 2, chloroplastic |
| sind01200 Carbon metabolism | i1_LQ_c67740_f1p0_1601_m.6729 | 74 | Phosphoglycerate kinase, cytosolic |
| sind01200 Carbon metabolism | 11884_0.path0_m.2391 | 40 | Fructose-bisphosphate aldolase 6, cytosolic |
| sind01200 Carbon metabolism | 11884_0.path0_m.2391 | 357 | Fructose-bisphosphate aldolase 6, cytosolic |
| sind00710 Carbon fixation in photosynthetic organisms | 3773_0.path1_m.6408 | 229 | NAD-dependent malic enzyme 59 kDa isoform, mitochondrial |
| sind00710 Carbon fixation in photosynthetic organisms | i1_HQ_c41079_f2p0_1865_m.5127 | 54 | Glutamate--glyoxylate aminotransferase 2 |
| sind00710 Carbon fixation in photosynthetic organisms | 4658_0.path0_m.7768 | 148 | Phosphoglycerate kinase, chloroplastic |
| sind00710 Carbon fixation in photosynthetic organisms | i1_HQ_c14606_f2p0_1675_m.3241 | 179 | Aspartate aminotransferase, mitochondrial |
| sind00710 Carbon fixation in photosynthetic organisms | i1_HQ_c14606_f2p0_1675_m.3241 | 374 | Aspartate aminotransferase, mitochondrial |
| sind00710 Carbon fixation in photosynthetic organisms | 3879_0.path0_m.6640 | 99 | NAD-dependent malic enzyme 62 kDa isoform, mitochondrial |
| sind00710 Carbon fixation in photosynthetic organisms | 3879_0.path0_m.6640 | 299 | NAD-dependent malic enzyme 62 kDa isoform, mitochondrial |
| sind00710 Carbon fixation in photosynthetic organisms | 8394_0.path0_m.4400 | 119 | Glyceraldehyde-3-phosphate dehydrogenase B, chloroplastic |
| sind00710 Carbon fixation in photosynthetic organisms | 8394_0.path0_m.4400 | 271 | Glyceraldehyde-3-phosphate dehydrogenase B, chloroplastic |
| sind00710 Carbon fixation in photosynthetic organisms | 8394_0.path0_m.4400 | 91 | Glyceraldehyde-3-phosphate dehydrogenase B, chloroplastic |
| sind00710 Carbon fixation in photosynthetic organisms | 5638_0.path0_m.1009 | 190 | Ribulose bisphosphate carboxylase large chain |
| sind00710 Carbon fixation in photosynthetic organisms | 5638_0.path0_m.1009 | 182 | Ribulose bisphosphate carboxylase large chain |
| sind00710 Carbon fixation in photosynthetic organisms | 5638_0.path0_m.1009 | 39 | Ribulose bisphosphate carboxylase large chain |
| sind00710 Carbon fixation in photosynthetic organisms | 5638_0.path0_m.1009 | 341 | Ribulose bisphosphate carboxylase large chain |
| sind00710 Carbon fixation in photosynthetic organisms | 5638_0.path0_m.1009 | 153 | Ribulose bisphosphate carboxylase large chain |
| sind00710 Carbon fixation in photosynthetic organisms | 8673_0.path2_m.4855 | 11 | Ribulose bisphosphate carboxylase small chain, chloroplastic |
| sind00710 Carbon fixation in photosynthetic organisms | 8673_0.path2_m.4855 | 107 | Ribulose bisphosphate carboxylase small chain, chloroplastic |
| sind00710 Carbon fixation in photosynthetic organisms | 11384_0.path0_m.1588 | 308 | Sedoheptulose-1,7-bisphosphatase, chloroplastic |
| sind00710 Carbon fixation in photosynthetic organisms | 10152_0.path0_m.229 | 369 | Fructose-bisphosphate aldolase 2, chloroplastic |
| sind00710 Carbon fixation in photosynthetic organisms | 10152_0.path0_m.229 | 61 | Fructose-bisphosphate aldolase 2, chloroplastic |
| sind00710 Carbon fixation in photosynthetic organisms | 4476_0.path1_m.7434 | 411 | NADP-dependent malic enzyme |
| sind00710 Carbon fixation in photosynthetic organisms | i1_LQ_c67740_f1p0_1601_m.6729 | 74 | Phosphoglycerate kinase, cytosolic |
| sind00710 Carbon fixation in photosynthetic organisms | 11884_0.path0_m.2391 | 40 | Fructose-bisphosphate aldolase 6, cytosolic |
| sind00710 Carbon fixation in photosynthetic organisms | 11884_0.path0_m.2391 | 357 | Fructose-bisphosphate aldolase 6, cytosolic |
| sind00250 Alanine, aspartate and glutamate metabolism | i1_HQ_c41079_f2p0_1865_m.5127 | 54 | Glutamate--glyoxylate aminotransferase 2 |
| sind00250 Alanine, aspartate and glutamate metabolism | 9538_0.path0_m.5901 | 354 | Glutamine synthetase, chloroplastic |
| sind00250 Alanine, aspartate and glutamate metabolism | 9538_0.path0_m.5901 | 101 | Glutamine synthetase, chloroplastic |
| sind00250 Alanine, aspartate and glutamate metabolism | 9538_0.path0_m.5901 | 168 | Glutamine synthetase, chloroplastic |
| sind00250 Alanine, aspartate and glutamate metabolism | 6440_0.path0_m.2112 | 312 | Glutamine synthetase cytosolic isozyme |
| sind00250 Alanine, aspartate and glutamate metabolism | 6440_0.path0_m.2112 | 289 | Glutamine synthetase cytosolic isozyme |
| sind00250 Alanine, aspartate and glutamate metabolism | 6440_0.path0_m.2112 | 322 | Glutamine synthetase cytosolic isozyme |
| sind00250 Alanine, aspartate and glutamate metabolism | i1_HQ_c14606_f2p0_1675_m.3241 | 179 | Aspartate aminotransferase, mitochondrial |
| sind00250 Alanine, aspartate and glutamate metabolism | i1_HQ_c14606_f2p0_1675_m.3241 | 374 | Aspartate aminotransferase, mitochondrial |
| sind00250 Alanine, aspartate and glutamate metabolism | 11907_0.path0_m.2413 | 311 | Glutamine synthetase cytosolic isozyme |
| sind01210 2-Oxocarboxylic acid metabolism | i1_HQ_c41079_f2p0_1865_m.5127 | 54 | Glutamate--glyoxylate aminotransferase 2 |
| sind01210 2-Oxocarboxylic acid metabolism | 9209_0.path0_m.5429 | 228 | Citrate synthase, glyoxysomal |
| sind01210 2-Oxocarboxylic acid metabolism | i1_HQ_c14606_f2p0_1675_m.3241 | 179 | Aspartate aminotransferase, mitochondrial |
| sind01210 2-Oxocarboxylic acid metabolism | i1_HQ_c14606_f2p0_1675_m.3241 | 374 | Aspartate aminotransferase, mitochondrial |
| sind01210 2-Oxocarboxylic acid metabolism | 1589_0.path0_m.3099 | 128 | Aconitate hydratase 2, mitochondrial |
| sind01210 2-Oxocarboxylic acid metabolism | 1589_0.path0_m.3099 | 122 | Aconitate hydratase 2, mitochondrial |
| sind01210 2-Oxocarboxylic acid metabolism | 1589_0.path0_m.3099 | 558 | Aconitate hydratase 2, mitochondrial |
| sind01210 2-Oxocarboxylic acid metabolism | 1589_0.path0_m.3099 | 381 | Aconitate hydratase 2, mitochondrial |
| sind01210 2-Oxocarboxylic acid metabolism | 1589_0.path0_m.3099 | 114 | Aconitate hydratase 2, mitochondrial |
| sind01210 2-Oxocarboxylic acid metabolism | 1589_0.path0_m.3099 | 166 | Aconitate hydratase 2, mitochondrial |
| sind01210 2-Oxocarboxylic acid metabolism | 1589_0.path0_m.3099 | 844 | Aconitate hydratase 2, mitochondrial |
| sind01210 2-Oxocarboxylic acid metabolism | 1589_0.path0_m.3099 | 855 | Aconitate hydratase 2, mitochondrial |
| sind01210 2-Oxocarboxylic acid metabolism | 1589_0.path0_m.3099 | 508 | Aconitate hydratase 2, mitochondrial |
| sind01210 2-Oxocarboxylic acid metabolism | 1589_0.path0_m.3099 | 574 | Aconitate hydratase 2, mitochondrial |
| sind01210 2-Oxocarboxylic acid metabolism | 9257_0.path1_m.5500 | 401 | Citrate synthase, mitochondrial |
| sind01210 2-Oxocarboxylic acid metabolism | 9257_0.path1_m.5500 | 346 | Citrate synthase, mitochondrial |
| sind01210 2-Oxocarboxylic acid metabolism | 8601_0.path0_m.4749 | 205 | Isocitrate dehydrogenase [NAD] catalytic subunit 5, mitochondrial |
| sind01210 2-Oxocarboxylic acid metabolism | 8601_0.path0_m.4749 | 332 | Isocitrate dehydrogenase [NAD] catalytic subunit 5, mitochondrial |
| sind01210 2-Oxocarboxylic acid metabolism | 8601_0.path0_m.4749 | 168 | Isocitrate dehydrogenase [NAD] catalytic subunit 5, mitochondrial |
| sind01210 2-Oxocarboxylic acid metabolism | 2789_0.path4_m.5221 | 83 | Aconitate hydratase 2, mitochondrial |
| sind00620 Pyruvate metabolism | 3773_0.path1_m.6408 | 229 | NAD-dependent malic enzyme 59 kDa isoform, mitochondrial |
| sind00620 Pyruvate metabolism | 3879_0.path0_m.6640 | 99 | NAD-dependent malic enzyme 62 kDa isoform, mitochondrial |
| sind00620 Pyruvate metabolism | 3879_0.path0_m.6640 | 299 | NAD-dependent malic enzyme 62 kDa isoform, mitochondrial |
| sind00620 Pyruvate metabolism | 9636_0.path0_m.6039 | 76 | Pyruvate dehydrogenase E1 component subunit beta-1, mitochondrial |
| sind00620 Pyruvate metabolism | 9666_0.path0_m.6084 | 157 | Dihydrolipoyllysine-residue acetyltransferase component 3 of pyruvate dehydrogenase complex, mitochondrial |
| sind00620 Pyruvate metabolism | 9666_0.path0_m.6084 | 150 | Dihydrolipoyllysine-residue acetyltransferase component 3 of pyruvate dehydrogenase complex, mitochondrial |
| sind00620 Pyruvate metabolism | 7112_0.path0_m.2939 | 371 | Pyruvate dehydrogenase E1 component subunit alpha, mitochondrial |
| sind00620 Pyruvate metabolism | 7112_0.path0_m.2939 | 333 | Pyruvate dehydrogenase E1 component subunit alpha, mitochondrial |
| sind00620 Pyruvate metabolism | 7112_0.path0_m.2939 | 354 | Pyruvate dehydrogenase E1 component subunit alpha, mitochondrial |
| sind00620 Pyruvate metabolism | 7112_0.path0_m.2939 | 239 | Pyruvate dehydrogenase E1 component subunit alpha, mitochondrial |
| sind00620 Pyruvate metabolism | 7112_0.path0_m.2939 | 322 | Pyruvate dehydrogenase E1 component subunit alpha, mitochondrial |
| sind00620 Pyruvate metabolism | 4476_0.path1_m.7434 | 411 | NADP-dependent malic enzyme |
| sind00620 Pyruvate metabolism | i1_LQ_c77746_f1p0_1530_m.131 | 223 | Probable acetyl-CoA acetyltransferase, cytosolic 2 |
| sind00620 Pyruvate metabolism | 5592_0.path0_m.933 | 306 | Dihydrolipoyl dehydrogenase 1, mitochondrial |
| sind00620 Pyruvate metabolism | 5592_0.path0_m.933 | 181 | Dihydrolipoyl dehydrogenase 1, mitochondrial |
| sind00620 Pyruvate metabolism | 5592_0.path0_m.933 | 207 | Dihydrolipoyl dehydrogenase 1, mitochondrial |
| sind00620 Pyruvate metabolism | 5592_0.path0_m.933 | 97 | Dihydrolipoyl dehydrogenase 1, mitochondrial |
| sind00620 Pyruvate metabolism | 5592_0.path0_m.933 | 218 | Dihydrolipoyl dehydrogenase 1, mitochondrial |
| sind00620 Pyruvate metabolism | 5592_0.path0_m.933 | 176 | Dihydrolipoyl dehydrogenase 1, mitochondrial |
| sind00620 Pyruvate metabolism | 5592_0.path0_m.933 | 448 | Dihydrolipoyl dehydrogenase 1, mitochondrial |
| sind00620 Pyruvate metabolism | 5592_0.path0_m.933 | 205 | Dihydrolipoyl dehydrogenase 1, mitochondrial |
| sind00620 Pyruvate metabolism | 5592_0.path0_m.933 | 166 | Dihydrolipoyl dehydrogenase 1, mitochondrial |
| sind00620 Pyruvate metabolism | 5592_0.path0_m.933 | 173 | Dihydrolipoyl dehydrogenase 1, mitochondrial |
| sind00620 Pyruvate metabolism | 5592_0.path0_m.933 | 354 | Dihydrolipoyl dehydrogenase 1, mitochondrial |
| sind00620 Pyruvate metabolism | 5592_0.path0_m.933 | 133 | Dihydrolipoyl dehydrogenase 1, mitochondrial |
| sind00620 Pyruvate metabolism | i1_LQ_c26180_f2p0_1342_m.6236 | 203 | Dihydrolipoyllysine-residue acetyltransferase component 1 of pyruvate dehydrogenase complex, mitochondrial |
| sind00130 Ubiquinone and other terpenoid-quinone biosynthesis | 7493_0.path1_m.3233 | 197 | Probable NAD(P)H dehydrogenase (quinone) FQR1-like 1 |
| sind00130 Ubiquinone and other terpenoid-quinone biosynthesis | i1_LQ_c90359_f1p0_1008_m.5193 | 216 | Probable NAD(P)H dehydrogenase (quinone) FQR1-like 1 |
| sind00130 Ubiquinone and other terpenoid-quinone biosynthesis | i1_LQ_c90359_f1p0_1008_m.5193 | 223 | Probable NAD(P)H dehydrogenase (quinone) FQR1-like 1 |
| sind00460 Cyanoamino acid metabolism | 9685_0.path0_m.6107 | 401 | Serine hydroxymethyltransferase, mitochondrial |
| sind00460 Cyanoamino acid metabolism | 9685_0.path0_m.6107 | 412 | Serine hydroxymethyltransferase, mitochondrial |
| sind00460 Cyanoamino acid metabolism | 9685_0.path0_m.6107 | 185 | Serine hydroxymethyltransferase, mitochondrial |
| sind00460 Cyanoamino acid metabolism | 9685_0.path0_m.6107 | 471 | Serine hydroxymethyltransferase, mitochondrial |
| sind00460 Cyanoamino acid metabolism | 9685_0.path0_m.6107 | 507 | Serine hydroxymethyltransferase, mitochondrial |
| sind00460 Cyanoamino acid metabolism | 5201_0.path1_m.207 | 386 | Raucaffricine-O-beta-D-glucosidase |
| sind00460 Cyanoamino acid metabolism | 6115_0.path0_m.1574 | 266 | Beta-glucosidase 12 |
| sind00460 Cyanoamino acid metabolism | 6115_0.path0_m.1574 | 113 | Beta-glucosidase 12 |
| sind00190 Oxidative phosphorylation | 2769_0.path0_m.5164 | 466 | ATP synthase subunit alpha, chloroplastic |
| sind00190 Oxidative phosphorylation | 2769_0.path0_m.5164 | 114 | ATP synthase subunit alpha, chloroplastic |
| sind00190 Oxidative phosphorylation | 4897_0.path0_m.8229 | 164 | Succinate dehydrogenase [ubiquinone] flavoprotein subunit 1, mitochondrial |
| sind00190 Oxidative phosphorylation | 4897_0.path0_m.8229 | 318 | Succinate dehydrogenase [ubiquinone] flavoprotein subunit 1, mitochondrial |
| sind00190 Oxidative phosphorylation | i1_LQ_c51375_f1p0_1803_m.7249 | 94 | ATP synthase subunit beta, mitochondrial |
| sind00190 Oxidative phosphorylation | i1_LQ_c51375_f1p0_1803_m.7249 | 86 | ATP synthase subunit beta, mitochondrial |
| sind00190 Oxidative phosphorylation | 4269_0.path0_m.7401 | 438 | V-type proton ATPase subunit B2 |
| sind00190 Oxidative phosphorylation | 6328_0.path0_m.1926 | 291 | Cytochrome c1 2, heme protein, mitochondrial |
| sind00190 Oxidative phosphorylation | i0_LQ_c59477_f1p0_991_m.4245 | 149 | ATP synthase subunit d, mitochondrial |
| sind00190 Oxidative phosphorylation | i0_LQ_c59477_f1p0_991_m.4245 | 87 | ATP synthase subunit d, mitochondrial |
| sind00190 Oxidative phosphorylation | i0_LQ_c59477_f1p0_991_m.4245 | 7 | ATP synthase subunit d, mitochondrial |
| sind00190 Oxidative phosphorylation | i0_LQ_c59477_f1p0_991_m.4245 | 80 | ATP synthase subunit d, mitochondrial |
| sind00190 Oxidative phosphorylation | i0_LQ_c59477_f1p0_991_m.4245 | 17 | ATP synthase subunit d, mitochondrial |
| sind00190 Oxidative phosphorylation | i0_LQ_c59477_f1p0_991_m.4245 | 100 | ATP synthase subunit d, mitochondrial |
| sind00190 Oxidative phosphorylation | i0_LQ_c59477_f1p0_991_m.4245 | 14 | ATP synthase subunit d, mitochondrial |
| sind00190 Oxidative phosphorylation | i1_LQ_c70037_f1p0_1177_m.2262 | 94 | Cytochrome c oxidase subunit 5b-1, mitochondrial |
| sind00190 Oxidative phosphorylation | 3962_0.path0_m.6781 | 92 | ATP synthase subunit beta, mitochondrial |
| sind00190 Oxidative phosphorylation | 3962_0.path0_m.6781 | 100 | ATP synthase subunit beta, mitochondrial |
| sind00190 Oxidative phosphorylation | 4175_0.path0_m.7181 | 384 | ATP synthase subunit alpha, mitochondrial |
| sind00190 Oxidative phosphorylation | 4175_0.path0_m.7181 | 189 | ATP synthase subunit alpha, mitochondrial |
| sind00190 Oxidative phosphorylation | 4175_0.path0_m.7181 | 476 | ATP synthase subunit alpha, mitochondrial |
| sind00190 Oxidative phosphorylation | 4175_0.path0_m.7181 | 387 | ATP synthase subunit alpha, mitochondrial |
| sind00190 Oxidative phosphorylation | 4175_0.path0_m.7181 | 142 | ATP synthase subunit alpha, mitochondrial |
| sind00190 Oxidative phosphorylation | 4133_0.path3_m.7108 | 70 | ATP synthase subunit beta, mitochondrial |
| sind00190 Oxidative phosphorylation | 4133_0.path3_m.7108 | 78 | ATP synthase subunit beta, mitochondrial |
| sind00190 Oxidative phosphorylation | 2708_0.path6_m.5056 | 575 | V-type proton ATPase catalytic subunit A |
| sind00190 Oxidative phosphorylation | 4700_0.path0_m.7851 | 97 | NADH dehydrogenase [ubiquinone] flavoprotein 1, mitochondrial |
| sind00190 Oxidative phosphorylation | 10112_0.path0_m.184 | 169 | ATP synthase subunit O, mitochondrial |
| sind00260 Glycine, serine and threonine metabolism | i1_HQ_c41079_f2p0_1865_m.5127 | 54 | Glutamate--glyoxylate aminotransferase 2 |
| sind00260 Glycine, serine and threonine metabolism | i0_LQ_c35465_f1p1_640_m.6645 | 115 | Aminomethyltransferase, mitochondrial |
| sind00260 Glycine, serine and threonine metabolism | 9685_0.path0_m.6107 | 401 | Serine hydroxymethyltransferase, mitochondrial |
| sind00260 Glycine, serine and threonine metabolism | 9685_0.path0_m.6107 | 412 | Serine hydroxymethyltransferase, mitochondrial |
| sind00260 Glycine, serine and threonine metabolism | 9685_0.path0_m.6107 | 185 | Serine hydroxymethyltransferase, mitochondrial |
| sind00260 Glycine, serine and threonine metabolism | 9685_0.path0_m.6107 | 471 | Serine hydroxymethyltransferase, mitochondrial |
| sind00260 Glycine, serine and threonine metabolism | 9685_0.path0_m.6107 | 507 | Serine hydroxymethyltransferase, mitochondrial |
| sind00260 Glycine, serine and threonine metabolism | 11093_0.path0_m.1258 | 154 | Glycine cleavage system H protein 3, mitochondrial |
| sind00260 Glycine, serine and threonine metabolism | 11093_0.path0_m.1258 | 140 | Glycine cleavage system H protein 3, mitochondrial |
| sind00260 Glycine, serine and threonine metabolism | 11093_0.path0_m.1258 | 47 | Glycine cleavage system H protein 3, mitochondrial |
| sind00260 Glycine, serine and threonine metabolism | 3539_0.path0_m.6388 | 867 | Glycine dehydrogenase (decarboxylating), mitochondrial |
| sind00260 Glycine, serine and threonine metabolism | 3539_0.path0_m.6388 | 700 | Glycine dehydrogenase (decarboxylating), mitochondrial |
| sind00260 Glycine, serine and threonine metabolism | 5592_0.path0_m.933 | 306 | Dihydrolipoyl dehydrogenase 1, mitochondrial |
| sind00260 Glycine, serine and threonine metabolism | 5592_0.path0_m.933 | 181 | Dihydrolipoyl dehydrogenase 1, mitochondrial |
| sind00260 Glycine, serine and threonine metabolism | 5592_0.path0_m.933 | 207 | Dihydrolipoyl dehydrogenase 1, mitochondrial |
| sind00260 Glycine, serine and threonine metabolism | 5592_0.path0_m.933 | 97 | Dihydrolipoyl dehydrogenase 1, mitochondrial |
| sind00260 Glycine, serine and threonine metabolism | 5592_0.path0_m.933 | 218 | Dihydrolipoyl dehydrogenase 1, mitochondrial |
| sind00260 Glycine, serine and threonine metabolism | 5592_0.path0_m.933 | 176 | Dihydrolipoyl dehydrogenase 1, mitochondrial |
| sind00260 Glycine, serine and threonine metabolism | 5592_0.path0_m.933 | 448 | Dihydrolipoyl dehydrogenase 1, mitochondrial |
| sind00260 Glycine, serine and threonine metabolism | 5592_0.path0_m.933 | 205 | Dihydrolipoyl dehydrogenase 1, mitochondrial |
| sind00260 Glycine, serine and threonine metabolism | 5592_0.path0_m.933 | 166 | Dihydrolipoyl dehydrogenase 1, mitochondrial |
| sind00260 Glycine, serine and threonine metabolism | 5592_0.path0_m.933 | 173 | Dihydrolipoyl dehydrogenase 1, mitochondrial |
| sind00260 Glycine, serine and threonine metabolism | 5592_0.path0_m.933 | 354 | Dihydrolipoyl dehydrogenase 1, mitochondrial |
| sind00260 Glycine, serine and threonine metabolism | 5592_0.path0_m.933 | 133 | Dihydrolipoyl dehydrogenase 1, mitochondrial |

**Table S4** The detailed information of the succinylated proteins in the protein-protein interaction network.

| **Protein accession** | **Gene ID** | **Protein description** | **Degree** | **Ksuc sites number** |
| --- | --- | --- | --- | --- |
| 7809_0.path0_m.3670 | m.3670 | Cytochrome b6-f complex iron-sulfur subunit, chloroplastic | 80 | 2 |
| 8394_0.path0_m.4400 | m.4400 | Glyceraldehyde-3-phosphate dehydrogenase B, chloroplastic | 60 | 3 |
| 5891_0.path0_m.1161 | m.1161 | ATP-citrate synthase beta chain protein 2 | 56 | 1 |
| 5592_0.path0_m.933 | m.933 | Dihydrolipoyl dehydrogenase 1, mitochondrial | 52 | 12 |
| i0_LQ_c128861_f1p0_857_m.7234 | m.7234 | Oxygen-evolving enhancer protein 3-1, chloroplastic | 52 | 8 |
| 7619_0.path0_m.3408 | m.3408 | Oxygen-evolving enhancer protein 2-1, chloroplastic | 52 | 2 |
| 7914_0.path0_m.3834 | m.3834 | Photosystem I reaction center subunit III, chloroplastic | 50 | 3 |
| 4520_0.path2_m.7505 | m.7505 | Glutathione reductase, chloroplastic (Fragment) | 50 | 1 |
| 6598_0.path0_m.2118 | m.2118 | Oxygen-evolving enhancer protein 1, chloroplastic | 48 | 7 |
| 8472_0.path0_m.4524 | m.4524 | Photosystem I reaction center subunit VI, chloroplastic | 48 | 2 |
| 8352_0.path0_m.4346 | m.4346 | Chlorophyll a-b binding protein CP26, chloroplastic | 48 | 1 |
| i1_HQ_c2074_f3p0_1549_m.7677 | m.7677 | Ribulose bisphosphate carboxylase_oxygenase activase 2, chloroplastic | 46 | 2 |
| 10152_0.path0_m.229 | m.229 | Fructose-bisphosphate aldolase 2, chloroplastic | 44 | 2 |
| 9257_0.path1_m.5500 | m.5500 | Citrate synthase, mitochondrial | 44 | 2 |
| i0_HQ_c151837_f2p8_620_m.7479 | m.7479 | Chlorophyll a-b binding protein 6, chloroplastic | 44 | 2 |
| 4897_0.path0_m.8229 | m.8229 | Succinate dehydrogenase [ubiquinone] flavoprotein subunit 1, mitochondrial | 44 | 2 |
| 9654_0.path0_m.6072 | m.6072 | Photosystem I reaction center subunit V, chloroplastic | 44 | 1 |
| 7467_0.path0_m.3184 | m.3184 | Chlorophyll a-b binding protein 8, chloroplastic | 42 | 1 |
| 9685_0.path0_m.6107 | m.6107 | Serine hydroxymethyltransferase, mitochondrial | 40 | 5 |
| 11093_0.path0_m.1258 | m.1258 | Glycine cleavage system H protein 3, mitochondrial | 40 | 3 |
| 4752_1.path0_m.7937 | m.7937 | Dihydrolipoyllysine-residue succinyltransferase component of 2-oxoglutarate dehydrogenase complex 2, mitochondrial | 38 | 3 |
| i1_LQ_c51375_f1p0_1803_m.7249 | m.7249 | ATP synthase subunit beta, mitochondrial | 38 | 2 |
| 9209_0.path0_m.5429 | m.5429 | Citrate synthase, glyoxysomal | 38 | 1 |
| 7383_0.path0_m.3334 | m.3334 | Chlorophyll a-b binding protein CP24 10A, chloroplastic | 38 | 1 |
| 6944_0.path0_m.2670 | m.2670 | ATP-citrate synthase alpha chain protein 2 | 36 | 2 |
| 9666_0.path0_m.6084 | m.6084 | Dihydrolipoyllysine-residue acetyltransferase component 3 of pyruvate dehydrogenase complex, mitochondrial | 36 | 2 |
| 7997_0.path0_m.3961 | m.3961 | Chloroplast stem-loop binding protein of 41 kDa b, chloroplastic | 36 | 2 |
| i1_LQ_c26180_f2p0_1342_m.6236 | m.6236 | Dihydrolipoyllysine-residue acetyltransferase component 1 of pyruvate dehydrogenase complex, mitochondrial | 36 | 1 |
| 11384_0.path0_m.1588 | m.1588 | Sedoheptulose-1,7-bisphosphatase, chloroplastic | 36 | 1 |
| i0_HQ_c28425_f2p22_869_m.4307 | m.4307 | Plastocyanin, chloroplastic | 36 | 1 |
| 9127_0.path0_m.5312 | m.5312 | Photosystem I reaction center subunit IV, chloroplastic | 36 | 1 |
| i0_LQ_c59477_f1p0_991_m.4245 | m.4245 | ATP synthase subunit d, mitochondrial | 34 | 7 |
| 5856_0.path0_m.1092 | m.1092 | Succinate--CoA ligase [ADP-forming] subunit beta, mitochondrial | 34 | 5 |
| 8810_0.path5_m.5063 | m.5063 | Photosystem I reaction center subunit II, chloroplastic | 34 | 3 |
| 2221_0.path0_m.4534 | m.4534 | 2-oxoglutarate dehydrogenase, mitochondrial | 34 | 1 |
| 6763_0.path0_m.2378 | m.2378 | Probable mitochondrial-processing peptidase subunit beta, mitochondrial | 34 | 1 |
| 6328_0.path0_m.1926 | m.1926 | Cytochrome c1 2, heme protein, mitochondrial | 32 | 1 |
| i0_LQ_c35465_f1p1_640_m.6645 | m.6645 | Aminomethyltransferase, mitochondrial | 32 | 1 |
| i0_LQ_c391587_f1p0_639_m.4102 | m.4102 | Peroxiredoxin Q, chloroplastic | 30 | 2 |
| 10025_0.path0_m.37 | m.37 | Chlorophyll a-b binding protein of LHCII type I, chloroplastic (Fragment) | 30 | 1 |
| i1_HQ_c32623_f2p0_1256_m.6619 | m.6619 | Succinate--CoA ligase [ADP-forming] subunit alpha-2, mitochondrial | 30 | 1 |
| 2769_0.path0_m.5164 | m.5164 | ATP synthase subunit alpha, chloroplastic | 28 | 2 |
| i1_HQ_c41079_f2p0_1865_m.5127 | m.5127 | Glutamate--glyoxylate aminotransferase 2 | 28 | 1 |
| i0_LQ_c102321_f1p1_510_m.5175 | m.5175 | Photosystem II 10 kDa polypeptide, chloroplastic | 28 | 1 |
| 10229_0.path0_m.317 | m.317 | Photosystem II 5 kDa protein, chloroplastic | 28 | 1 |
| 4175_0.path0_m.7181 | m.7181 | ATP synthase subunit alpha, mitochondrial | 26 | 5 |
| 7112_0.path0_m.2939 | m.2939 | Pyruvate dehydrogenase E1 component subunit alpha, mitochondrial | 26 | 5 |
| 3879_0.path0_m.6640 | m.6640 | NAD-dependent malic enzyme 62 kDa isoform, mitochondrial | 26 | 2 |
| 8673_0.path2_m.4855 | m.4855 | Ribulose bisphosphate carboxylase small chain, chloroplastic | 26 | 2 |
| 10112_0.path0_m.184 | m.184 | ATP synthase subunit O, mitochondrial | 26 | 1 |
| 3773_0.path1_m.6408 | m.6408 | NAD-dependent malic enzyme 59 kDa isoform, mitochondrial | 26 | 1 |
| 9636_0.path0_m.6039 | m.6039 | Pyruvate dehydrogenase E1 component subunit beta-1, mitochondrial | 26 | 1 |
| 5071_0.path1_m.2 | m.2 | Photosystem I P700 chlorophyll a apoprotein A2 | 26 | 1 |
| 5250_0.path0_m.317 | m.317 | Glyoxysomal fatty acid beta-oxidation multifunctional protein MFP-a | 24 | 3 |
| 4476_0.path1_m.7434 | m.7434 | NADP-dependent malic enzyme | 24 | 1 |
| 8601_0.path0_m.4749 | m.4749 | Isocitrate dehydrogenase [NAD] catalytic subunit 5, mitochondrial | 22 | 3 |
| 3539_0.path0_m.6388 | m.6388 | Glycine dehydrogenase (decarboxylating), mitochondrial | 22 | 2 |
| i1_LQ_c77746_f1p0_1530_m.131 | m.131 | Probable acetyl-CoA acetyltransferase, cytosolic 2 | 22 | 1 |
| i1_LQ_c67740_f1p0_1601_m.6729 | m.6729 | Phosphoglycerate kinase, cytosolic | 22 | 1 |
| 9538_0.path0_m.5901 | m.5901 | Glutamine synthetase, chloroplastic | 20 | 3 |
| 6706_0.path0_m.2288 | m.2288 | Cytochrome f | 20 | 3 |
| 4700_0.path0_m.7851 | m.7851 | NADH dehydrogenase [ubiquinone] flavoprotein 1, mitochondrial | 20 | 1 |
| i1_LQ_c48019_f1p0_1783_m.5563 | m.5563 | Elongation factor Tu, chloroplastic | 20 | 1 |
| i1_HQ_c14606_f2p0_1675_m.3241 | m.3241 | Aspartate aminotransferase, mitochondrial | 18 | 2 |
| 8752_0.path0_m.4971 | m.4971 | Mitochondrial phosphate carrier protein 3, mitochondrial | 18 | 2 |
| 7598_0.path21_m.3382 | m.3382 | Chlorophyll a-b binding protein of LHCII type I, chloroplastic (Fragment) | 18 | 1 |
| 9425_0.path0_m.5723 | m.5723 | Elongation factor Tu, mitochondrial | 18 | 1 |
| 5638_0.path0_m.1009 | m.1009 | Ribulose bisphosphate carboxylase large chain | 16 | 5 |
| 5136_0.path0_m.111 | m.111 | Photosystem II CP47 reaction center protein | 16 | 3 |
| 2789_0.path4_m.5221 | m.5221 | Aconitate hydratase 2, mitochondrial | 16 | 1 |
| 5772_0.path0_m.1247 | m.1247 | L-ascorbate peroxidase T, chloroplastic | 14 | 4 |
| 6817_0.path0_m.2451 | m.2451 | Adenylate kinase 4 | 14 | 2 |
| 8576_0.path0_m.4715 | m.4715 | 2-Cys peroxiredoxin BAS1-like, chloroplastic | 14 | 2 |
| 6440_0.path0_m.2112 | m.2112 | Glutamine synthetase cytosolic isozyme | 12 | 3 |
| i1_LQ_c70037_f1p0_1177_m.2262 | m.2262 | Cytochrome c oxidase subunit 5b-1, mitochondrial | 12 | 1 |
| 11450_0.path0_m.1663 | m.1663 | Prohibitin-3, mitochondrial | 12 | 1 |
| 7579_0.path0_m.3351 | m.3351 | ADP,ATP carrier protein 1, mitochondrial | 10 | 10 |
| i2_LQ_c49747_f1p2_2285_m.3131 | m.3131 | RuBisCO large subunit-binding protein subunit beta, chloroplastic | 10 | 4 |
| i0_LQ_c3565_f1p2_807_m.6245 | m.6245 | Superoxide dismutase [Mn], mitochondrial | 10 | 4 |
| 4485_0.path0_m.7451 | m.7451 | RuBisCO large subunit-binding protein subunit alpha, chloroplastic | 10 | 1 |
| 11884_0.path0_m.2391 | m.2391 | Fructose-bisphosphate aldolase 6, cytosolic | 8 | 2 |
| i1_LQ_c32654_f1p0_1326_m.4263 | m.4263 | Uncharacterized protein At2g37660, chloroplastic | 8 | 2 |
| 8750_0.path0_m.4965 | m.4965 | L-ascorbate peroxidase 3, peroxisomal | 8 | 1 |
| 9658_0.path1_m.6076 | m.6076 | Monodehydroascorbate reductase | 8 | 1 |
| 8051_0.path0_m.4044 | m.4044 | Monodehydroascorbate reductase | 8 | 1 |
| 6522_0.path0_m.2251 | m.2251 | 2-methylene-furan-3-one reductase | 8 | 1 |
| 7333_0.path7_m.3258 | m.3258 | ADP,ATP carrier protein | 8 | 1 |
| i1_HQ_c9639_f2p1_1104_m.1587 | m.1587 | Mitochondrial outer membrane protein porin of 34 kDa | 8 | 1 |
| 7440_0.path0_m.3146 | m.3146 | Gamma carbonic anhydrase 1, mitochondrial | 8 | 1 |
| i1_LQ_c14179_f1p0_1554_m.5223 | m.5223 | Uncharacterized protein At2g27730, mitochondrial | 8 | 1 |
| 8789_0.path0_m.5031 | m.5031 | Probable ATP synthase 24 kDa subunit, mitochondrial | 6 | 3 |
| 3549_0.path0_m.6407 | m.6407 | Leucine aminopeptidase 2, chloroplastic | 6 | 1 |
| 10683_0.path0_m.953 | m.953 | Formate dehydrogenase, mitochondrial | 6 | 1 |
| 7428_0.path0_m.3131 | m.3131 | Glutathione S-transferase T1 | 6 | 1 |
| i2_LQ_c96775_f1p0_2002_m.4473 | m.4473 | Glutathione S-transferase F11 | 6 | 1 |
| 4658_0.path0_m.7768 | m.7768 | Phosphoglycerate kinase, chloroplastic | 6 | 1 |
| 8824_0.path0_m.5082 | m.5082 | Peroxiredoxin-2B | 6 | 1 |
| 2708_0.path6_m.5056 | m.5056 | V-type proton ATPase catalytic subunit A | 4 | 1 |
| i1_LQ_c36007_f1p0_1069_m.13 | m.13 | Protein IN2-1 homolog B | 2 | 4 |
| 8146_0.path0_m.4182 | m.4182 | Chlorophyll a-b binding protein 36, chloroplastic | 2 | 2 |
| 9679_0.path0_m.6099 | m.6099 | NADH-cytochrome b5 reductase-like protein | 2 | 1 |
| i1_LQ_c36180_f1p0_1059_m.2215 | m.2215 | Acylpyruvase FAHD1, mitochondrial | 2 | 1 |
| i0_LQ_c104330_f1p1_837_m.4398 | m.4398 | Peroxisomal (S)-2-hydroxy-acid oxidase GLO2 | 2 | 1 |
| 8076_0.path0_m.4073 | m.4073 | CBS domain-containing protein CBSX3, mitochondrial | 2 | 1 |
| i0_HQ_c174165_f5p1_410_m.84 | m.84 | Succinate dehydrogenase subunit 7B, mitochondrial | 2 | 1 |
